# Supplementary material for: A burden of proof study on alcohol consumption and ischemic heart disease
Source: Nat Commun. 2024 May 14;15:4082. doi: 10.1038/s41467-024-47632-7 (PMC11094064; doi:10.1038/s41467-024-47632-7)
Supplement: Supplementary file 1 — Supplementary Information [file 41467_2024_47632_MOESM1_ESM.pdf]

Supplementary Information for "A burden of proof study on alcohol consumption and ischemic heart disease"

Table of Contents

Section 1: PRISMA flow diagram & checklists 2
Section 2: GATHER checklist 9
Section 3: Data source extraction 11
Section 4: Study characteristics 16
Section 5: Study quality and risk of bias assessment 32
Section 6: Results from individual studies 41
Section 7: Risk curve details 57
Section 8. Details on statistical methods 61
Section 9: Subanalyses 63
Section 10: Sensitivity analyses 66
References 75

Section 1: PRISMA flow diagram & checklists

Figure S1. PRISMA 2020 flow diagram

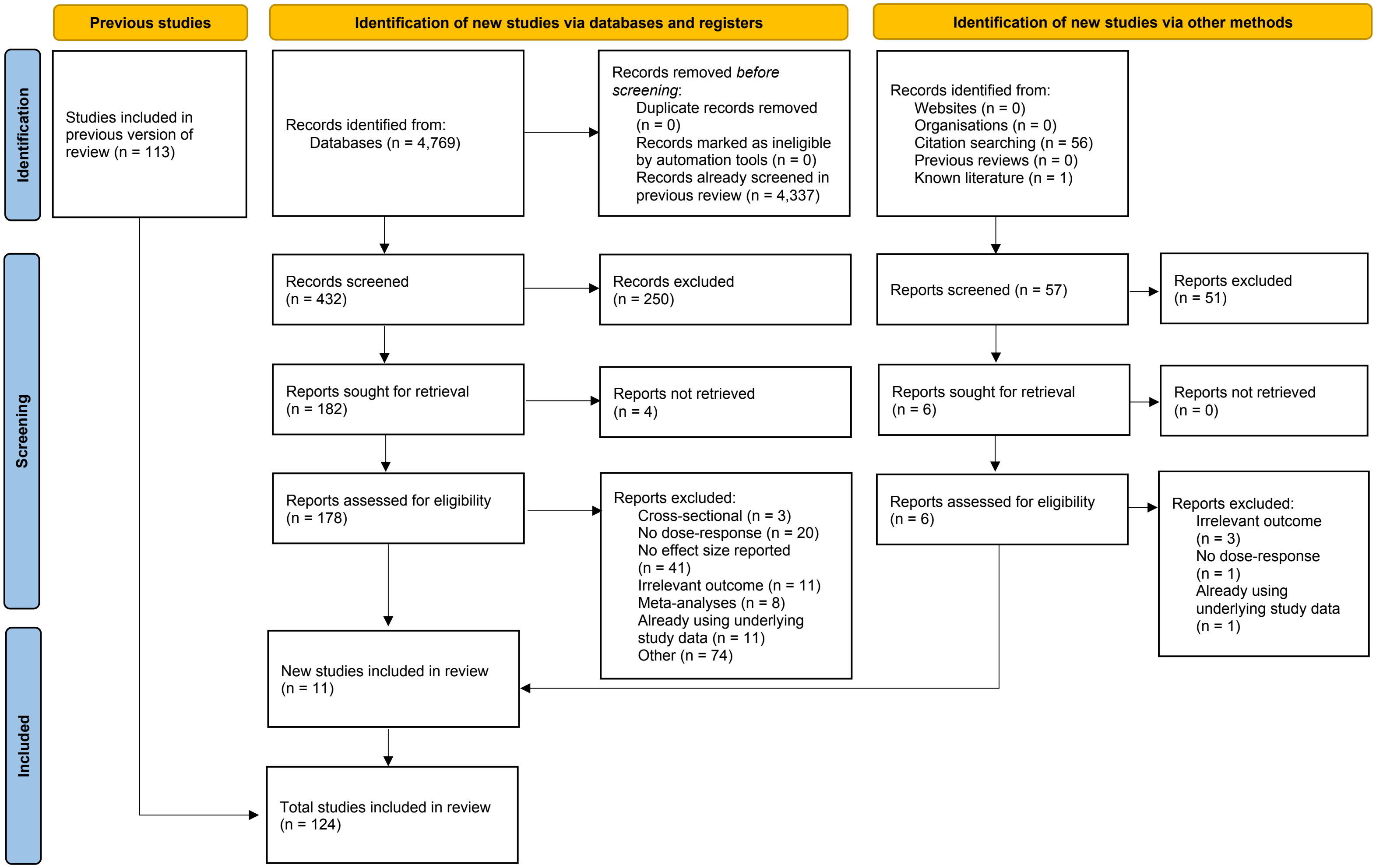

Note. In total, 95 cohort studies, 27 case-control studies, and five Mendelian randomization (MR) studies were included. All MR studies reported effect size estimates obtained using conventional methods from cohort data. As the exact underlying data were not used in any of the included cohort studies, we included data from three MR studies from our main analyses as cohort studies.

Table S1. PRISMA 2020 checklist

Section and Topic	Item #	Checklist item	Location where item is reported
**TITLE**			
Title	1	Identify the report as a systematic review.	Systematic review is not mentioned in the title, but in the abstract.
**ABSTRACT**			
Abstract	2	See the PRISMA 2020 for Abstracts checklist.	See PRISMA 2020 for Abstracts Checklist below (Table S2)
**INTRODUCTION**			
Rationale	3	Describe the rationale for the review in the context of existing knowledge.	"Introduction" paragraphs 1–5
Objectives	4	Provide an explicit statement of the objective(s) or question(s) the review addresses.	"Introduction" paragraph 6, 8
**METHODS**			
Eligibility criteria	5	Specify the inclusion and exclusion criteria for the review and how studies were grouped for the syntheses.	Inclusion and exclusion criteria are listed in Methods section "Conducting the systematic review" paragraph 1; reasons for exclusion and number of studies excluded also provided in PRISMA flow diagram (Supplementary Information Figure S1)
Information sources	6	Specify all databases, registers, websites, organisations, reference lists and other sources searched or consulted to identify studies. Specify the date when each source was last searched or consulted.	Methods section "Conducting the systematic review" paragraph 1
Search strategy	7	Present the full search strategies for all databases, registers and websites, including any filters and limits used.	Methods section "Conducting the systematic review" paragraph 1
Selection process	8	Specify the methods used to decide whether a study met the inclusion criteria of the review, including how many reviewers screened each record and each report retrieved, whether they worked independently, and if applicable, details of automation tools used in the process.	Methods section "Conducting the systematic review" paragraph 1
Data collection process	9	Specify the methods used to collect data from reports, including how many reviewers collected data from each report, whether they worked independently, any processes for obtaining or confirming data from study investigators, and if applicable, details of automation tools used in the process.	Methods section "Conducting the systematic review" paragraph 1
Data items	10a	List and define all outcomes for which data were sought. Specify whether all results that were compatible with each outcome domain in each study were sought (e.g. for all measures, time points, analyses), and if not, the methods used to decide which results to collect.	Methods sections "Overview" paragraphs 1 and 2 and "Conducting the systematic the review" paragraph 1
	10b	List and define all other variables for which data were sought (e.g. participant and intervention characteristics, funding sources). Describe any assumptions made about	Methods section "Conducting the systematic review";

Table S1. PRISMA 2020 checklist

Section and Topic	Item #	Checklist item	Location where item is reported
		any missing or unclear information.	study characteristics for each included study are also listed in Supplementary Information Table S5 and S6
Study risk of bias assessment	11	Specify the methods used to assess risk of bias in the included studies, including details of the tool(s) used, how many reviewers assessed each study and whether they worked independently, and if applicable, details of automation tools used in the process.	Overview of methods for testing for bias in main text methods section "Testing and adjusting for biases across study designs and characteristics"; information on quantified bias covariates is provided in Supplementary Information section 5
Effect measures	12	Specify for each outcome the effect measure(s) (e.g. risk ratio, mean difference) used in the synthesis or presentation of results.	"Introduction" paragraph 7; methods "Overview", "Estimating the shape of the risk-outcome relationship", and "Estimating the burden of proof risk function" sections
Synthesis methods	13a	Describe the processes used to decide which studies were eligible for each synthesis (e.g. tabulating the study intervention characteristics and comparing against the planned groups for each synthesis (item #5)).	Methods section "Conducting the systematic review"; outliering strategy described in methods section "Overview" and "Estimating the shape of the risk-outcome relationship"
	13b	Describe any methods required to prepare the data for presentation or synthesis, such as handling of missing summary statistics, or data conversions.	Methods section "Overview" paragraph 2 and 3, and "Conducting the systematic review" paragraph 2
	13c	Describe any methods used to tabulate or visually display results of individual studies and syntheses.	Methods sections "Evaluating potential for publication or reporting bias" and "Estimating the burden of proof risk function"
	13d	Describe any methods used to synthesize results and provide a rationale for the choice(s). If meta-analysis was performed, describe the model(s), method(s) to identify the presence and extent of statistical heterogeneity, and software package(s) used.	Methods sections "Estimating the shape of the risk-outcome relationship", "Quantifying between-study heterogeneity, accounting for heterogeneity, uncertainty, and small numbers of studies", and "Estimating the burden

Table S1. PRISMA 2020 checklist

Section and Topic	Item #	Checklist item	Location where item is reported
			of proof risk function”
	13e	Describe any methods used to explore possible causes of heterogeneity among study results (e.g. subgroup analysis, meta-regression).	Methods sections “Overview” paragraphs 2 and 3, “Testing and adjusting for biases across study designs and characteristics”, and “Quantifying between-study heterogeneity, accounting for heterogeneity, uncertainty, and small numbers of studies”
	13f	Describe any sensitivity analyses conducted to assess robustness of the synthesized results.	Methods sections “Overview” paragraphs 2 and 3 and “Estimating the shape of the risk-outcome relationship”; Supplementary Information section 10
Reporting bias assessment	14	Describe any methods used to assess risk of bias due to missing results in a synthesis (arising from reporting biases).	Methods section “Evaluating potential for publication or reporting bias”
Certainty assessment	15	Describe any methods used to assess certainty (or confidence) in the body of evidence for an outcome.	Methods section “Quantifying between-study heterogeneity, accounting for heterogeneity, uncertainty, and small numbers of studies”
**RESULTS**			
Study selection	16a	Describe the results of the search and selection process, from the number of records identified in the search to the number of studies included in the review, ideally using a flow diagram.	PRISMA flow diagram (Supplementary Information Figure S1)
	16b	Cite studies that might appear to meet the inclusion criteria, but which were excluded, and explain why they were excluded.	N/A
Study characteristics	17	Cite each included study and present its characteristics.	Supplementary Information section 4 and references, and the GHDx website (https://ghdx.healthdata.org/record/ihme-data/gbd-alcohol-ihd-bop-risk-outcome-scores)
Risk of bias in studies	18	Present assessments of risk of bias for each included study.	Supplementary Information section 5
Results of individual studies	19	For all outcomes, present, for each study: (a) summary statistics for each group (where appropriate) and (b) an effect estimate and its precision (e.g. confidence/credible interval), ideally using structured tables or plots.	Supplementary Information section 6 Table S9
Results of syntheses	20a	For each synthesis, briefly summarise the characteristics and risk of bias among contributing studies.	First paragraph of each results section
	20b	Present results of all statistical syntheses conducted. If meta-analysis was done, present for each the summary estimate and its precision (e.g. confidence/credible interval) and	Second paragraph of each results section;

Table S1. PRISMA 2020 checklist

Section and Topic	Item #	Checklist item	Location where item is reported
		measures of statistical heterogeneity. If comparing groups, describe the direction of the effect.	Figures 1–4; Table 2; Supplementary Information section 7, 9, and 10
	20c	Present results of all investigations of possible causes of heterogeneity among study results.	All 95% uncertainty intervals presented in the main text and Supplementary Information incorporate between-study heterogeneity (unless specified otherwise); burden of proof risk functions, risk-outcome scores, and star ratings for each relative risk curve were calculated using uncertainty that incorporates between-study heterogeneity
	20d	Present results of all sensitivity analyses conducted to assess the robustness of the synthesized results.	Supplementary Information section 10
Reporting biases	21	Present assessments of risk of bias due to missing results (arising from reporting biases) for each synthesis assessed.	Second paragraph of each results section; funnel plots (Figures 1–4, S2–8); Tables 2, S13, and S14
Certainty of evidence	22	Present assessments of certainty (or confidence) in the body of evidence for each outcome assessed.	95% uncertainty intervals are given alongside all mean relative risk estimates in the Results section, Table 2, and Supplementary Information Tables S10, S13, and S14; all relative risk curve figures (Figures 1–4, Supplementary Information Figures S2–8) include shading to depict uncertainty intervals (both with and without between-study heterogeneity)
**DISCUSSION**			
Discussion	23a	Provide a general interpretation of the results in the context of other evidence.	Discussion paragraphs 2, 3, and 7
	23b	Discuss any limitations of the evidence included in the review.	Discussion paragraphs 3 and 6
	23c	Discuss any limitations of the review processes used.	Discussion paragraph 6
	23d	Discuss implications of the results for practice, policy, and future research.	Discussion paragraphs 4, 5, and 7; Table 1 (Research summary)
**OTHER INFORMATION**			

Table S1. PRISMA 2020 checklist

Section and Topic	Item #	Checklist item	Location where item is reported
Registration and protocol	24a	Provide registration information for the review, including register name and registration number, or state that the review was not registered.	The entirety of the Global Burden of Diseases, Injuries, and Risk Factors Study has been registered and approved through the UW IRB. The systematic review was not registered on its own.
	24b	Indicate where the review protocol can be accessed, or state that a protocol was not prepared.	A protocol was not prepared.
	24c	Describe and explain any amendments to information provided at registration or in the protocol.	N/A
Support	25	Describe sources of financial or non-financial support for the review, and the role of the funders or sponsors in the review.	"Acknowledgments" section of the manuscript
Competing interests	26	Declare any competing interests of review authors.	"Competing Interests Statement" section of the manuscript
Availability of data, code and other materials	27	Report which of the following are publicly available and where they can be found: template data collection forms; data extracted from included studies; data used for all analyses; analytic code; any other materials used in the review.	Data collection form template: Supplementary Information Table S4; Data extracted from included studies: Supplementary Information Tables S5–7 and S9, and "Data availability" section of the manuscript; Data used for all analyses: Source Data files; Analytic code: "Code availability" section of the manuscript

Table S2. PRISMA 2020 abstract checklist

Section and Topic	Item #	Checklist item	Reported (Yes/No)
**TITLE**			
Title	1	Identify the report as a systematic review.	Systematic review is not mentioned in the title, but in the abstract.
**BACKGROUND**			
Objectives	2	Provide an explicit statement of the main objective(s) or question(s) the review addresses.	Yes
**METHODS**			
Eligibility criteria	3	Specify the inclusion and exclusion criteria for the review.	Not in abstract, but in main text and Supplementary Information (due to word count limitations by the journal)
Information sources	4	Specify the information sources (e.g. databases, registers) used to identify studies and the date when each was last searched.	Not in abstract, but in main text and Supplementary Information (due to word count limitations by the journal)
Risk of bias	5	Specify the methods used to assess risk of bias in the included studies.	Not in abstract, but in main text and Supplementary Information (due to word count limitations by the journal)
Synthesis of results	6	Specify the methods used to present and synthesise results.	Yes
**RESULTS**			
Included studies	7	Give the total number of included studies and participants and summarise relevant characteristics of studies.	Not in abstract, but in main text and Supplementary Information (due to word count limitations by the journal)
Synthesis of results	8	Present results for main outcomes, preferably indicating the number of included studies and participants for each. If meta-analysis was done, report the summary estimate and confidence/credible interval. If comparing groups, indicate the direction of the effect (i.e. which group is favoured).	Yes, although due to word count limitations, the number of included studies and participants and estimated effect sizes are only reported in the main text and Supplementary Information
**DISCUSSION**			
Limitations of evidence	9	Provide a brief summary of the limitations of the evidence included in the review (e.g. study risk of bias, inconsistency and imprecision).	Not in abstract, just in main text
Interpretation	10	Provide a general interpretation of the results and important implications.	Yes
**OTHER**			
Funding	11	Specify the primary source of funding for the review.	Not in abstract, just main text
Registration	12	Provide the register name and registration number.	No

Section 2: GATHER checklist

Table S3. GATHER checklist

Item #	Checklist item	Reported on page #
Objectives and funding		
1	Define the indicator(s), populations (including age, sex, and geographic entities), and time period(s) for which estimates were made.	"Introduction" section paragraph 8; methods "Overview" section paragraphs 2 and 3
2	List the funding sources for the work.	"Acknowledgments" section
Data Inputs		
For all data inputs from multiple sources that are synthesized as part of the study:		
3	Describe how the data were identified and how the data were accessed.	Methods section "Conducting the systematic review"
4	Specify the inclusion and exclusion criteria. Identify all ad-hoc exclusions.	Inclusion and exclusion criteria listed in Methods section "Conducting the systematic review" paragraph 1; reasons for exclusion and number of studies excluded also provided in PRISMA flow diagram (Supplementary Information Figure S1)
5	Provide information on all included data sources and their main characteristics. For each data source used, report reference information or contact name/institution, population represented, data collection method, year(s) of data collection, sex and age range, diagnostic criteria or measurement method, and sample size, as relevant.	Supplementary Information section 4 and the GHDx website (https://ghdx.healthdata.org/record/ihme-data/gbd-alcohol-ihd-bop-risk-outcome-scores)
6	Identify and describe any categories of input data that have potentially important biases (e.g., based on characteristics listed in item 5).	Supplementary Information section 5
For data inputs that contribute to the analysis but were not synthesized as part of the study:		
7	Describe and give sources for any other data inputs.	N/A
For all data inputs:		
8	Provide all data inputs in a file format from which data can be efficiently extracted (e.g., a spreadsheet rather than a PDF), including all relevant meta-data listed in item 5. For any data inputs that cannot be shared because of ethical or legal reasons, such as third-party ownership, provide a contact name or the name of the institution that retains the right to the data.	Data inputs can be found on the GHDx website (https://ghdx.healthdata.org/record/ihme-data/gbd-alcohol-ihd-bop-risk-outcome-scores) and the Source Data files
Data analysis		
9	Provide a conceptual overview of the data analysis method. A diagram may be helpful.	"Introduction" section paragraph 7 and Methods section "Overview"
10	Provide a detailed description of all steps of the analysis, including mathematical formulae. This description should cover, as relevant, data cleaning, data pre-processing, data adjustments and weighting of data sources, and mathematical or statistical model(s).	Methods (all sections)
11	Describe how candidate models were evaluated and how the final model(s) were selected.	Methods section "Estimating the shape of the risk-outcome relationship"; Supplementary Information section 10 Figure S5a–l and Table S13
12	Provide the results of an evaluation of model performance, if done, as well as the results of any relevant sensitivity analysis.	Supplementary Information section 10

Table S3. GATHER checklist

Item #	Checklist item	Reported on page #
13	Describe methods for calculating uncertainty of the estimates. State which sources of uncertainty were, and were not, accounted for in the uncertainty analysis.	Methods sections "Estimating the shape of the risk-outcome relationship" and "Quantifying between-study heterogeneity, accounting for heterogeneity, uncertainty, and small numbers of studies"
14	State how analytic or statistical source code used to generate estimates can be accessed.	All code used for these analyses is publicly available online (https://github.com/ihmeuw-msca/burden-of-proof)
Results and Discussion		
15	Provide published estimates in a file format from which data can be efficiently extracted.	Published estimates are provided as Source Data files
16	Report a quantitative measure of the uncertainty of the estimates (e.g., uncertainty intervals).	95% uncertainty intervals are given for all findings, including in the text, figures, and tables in the main text and Supplementary Information
17	Interpret results in light of existing evidence. If updating a previous set of estimates, describe the reasons for changes in estimates.	"Discussion" section paragraphs 2, 3, and 7
18	Discuss limitations of the estimates. Include a discussion of any modelling assumptions or data limitations that affect interpretation of the estimates.	"Discussion" section paragraph 6

Section 3: Data source extraction

Table S4. Causal criteria extraction template

Category	Variable	Definition
Source	seq	
	underlying_nid	Underlying NID: Enter the underlying NID of the study (if applicable). Always talk to a data indexer if you don't know if an underlying NID is needed. They may be used for meta-analyses, certain database sources, and in some other specific cases.
	nid	Found in GHDx, created through the epi form, or created by Data Indexer
	field_citation_value	IHME Zotero format or if source has NID, citation info from GHDx
	file_path	Optional; full file path of article; Only needed if source doesn't have NID, to facilitate NID creation.
	meta_analysis_nid	Optional; if the study was found through a meta-analysis, enter the NID.
	source_type	Identifies the underlying mode of data collection.
Location	location_name	location name (from locations tab). Do a fast double-click in this field to get the drop-down menu, then start typing the location_name. For location_names with special characters, you may need to use the scroll bar.
	location_id	autopopulated from location_name
	smaller_site_unit	Were the study partcipants selected from a geography corresponding to a GBD location? 1=yes, 0=no
	site_memo	Open-text site field. Copy and paste verbatim from the source all the information regarding the location of data collection. Please include facility names (hospitals, clinics, etc) and street addresses, GPS coordinates, etc, if provided. Comprehensive detail is important here. See the Epi template documentation page in the HUB for more information.
	representativeness	Were the study participants representative of the population of the location in which the study was conducted? 1=yes, 0=no
Study Population	year_start	year the study was started. If not specified, leave blank
	year_end	year the study was finished (including most recent follow up). If not specified, leave blank
	year_issue	0 = no issue flagged; 1 = issue flagged for modeler; always include explanatory notes the note_SR column
	age_start	ages from 1 and above must be entered as an integer. Ages <1 can be entered as decimal values, e.g., 3 days = 3/365.
	age_end	ages from 1 and above must be entered as an integer. Ages <1 can be entered as decimal values, e.g., 3 days = 3/365.
	age_mean	Mean age
	age_sd	SD of age
	age_issue	0 = no issue flagged; 1 = issue flagged for modeler; always include explanatory notes the note_SR column
	sex	Sex identifier: Male, Female, Both
	percent_male	what percent of the population is male (0-1), if pop is all female, then it would be 0
	sex_issue	sex_issue
Study Design	design	Study design: Specify the design of the study
	study_name	Study Name: Enter the name of the study (e.g., Nurses' Health Study), if provided. Do not enter the title of the article.
Follow up	response_rate	For the family of cohort studies, specify the retention rate (%) at the end of the study. For the family of case-control studies, specify the response rate (%).
	response_rate_assess	Specify how the retention/response rate was defined in the study, including any calculations performed to get a value.
	duration_fup_measure	Type of follow up measure (i.e. mean, median, max, min)
	duration_fup_units	Units of follow up duration
	value_of_duration_fup	Enter the length of participant follow-up.
Risk	risk	Risk: Select the risk factor
	rei	Auto-populated from risk
	risk_def	Risk definition: Provide a brief description of the risk as reported in the study
	exp_method_1	Please specify the method of exposure assessment. If there are more than 1, please add in the next columns labeled "exp_method_2".

Table S4. Causal criteria extraction template

Category	Variable	Definition
Exposure	exp_method_2	Please specify the method of exposure assessment. If there are more than 2, please add in the next columns labeled "exp_method_3".
	exp_method_3	Please specify the method of exposure assessment.
	exp_instrument	Exposure assessment instrument: Specify the name of the exposure assessment instrument. For self-reported exposures, please specify the name of the questionnaire, eg, International Physical Activity Questionnaire (IPAQ). If more than one instrument was used, specify all. Do not enter a generic description of the instrument.
	exp_recall_period	This field describes the unit of exposure recall used in data collection ONLY for self-report. Select the correct option from the drop-down menu. If the unit is days, weeks, months, or years, please enter the number in exp_recall_period_value (next column). If the unit is 'lifetime', nothing needs to be entered in exp_recall_period_value. For example, if the study said the recall period was 4 weeks, enter 4 in exp_recall_period_value, and 'weeks' in the field exp_recall_period. If 'other' is selected, please describe in exp_recall_period_other
	exp_recall_period_value	If you entered days, weeks, months, or years in the field 'exp_recall_period', please enter the corresponding integer in this field. For example, if the study said the recall period was 4 weeks, enter 4 in exp_recall_period_value, and 'weeks' in the field exp_recall_period.
	exp_recall_period_other	If 'other' was selected in exp_recall_period, please describe the exposure recall period that the study specified (e.g., recall of exposure from 12 to 18 years).
	exp_type	Which form of the exposure was included in relative risk estimation analysis?
	exp_assess_level	Level of exposure assessment
	exp_assess_period	What is the frequency of exposure assessment?
	exp_assess_num	If multiple, specify the number of times that exposure was assessed (excluding baseline)
Exposure Measurements	alcohol_type	Select the type of alcohol associated to the effect size and exposure level in this row
	exp_group_def	Provide a brief description of the exposed group
	exp_level_value	If a point value is provided for the level of exposure (eg, average amount of cigarettes/day), enter the value here
	exp_level_value_type	If a value is entered in 'exp_level_value', select the type of measure of the value. For example, if the point value is average cigarettes/day, select 'mean'.
	exp_level_lower_sign	Can use > or >= in association the reported lower bound of an exposure range in the exp_level_lower field
	exp_level_lower	If a point exposure level is not reported in the exp_level_value field, use this column to enter the reported lower bound of an exposure range
	exp_level_upper_sign	Can use < or <= in association with the reported upper bound of an exposure range in the exp_level_upper field
	exp_level_upper	If a point exposure level is not reported in exp_level_value, use this column to enter the reported upper bound of an exposure range
	male_exp_level_value_type	If a value is entered in 'male_exp_level_value', select the type of measure of the value. For example, if the point value is average cigarettes/day, select 'mean'.
	male_exp_level_lower_sign	Can use > or >= in association the reported lower bound of an exposure range in the male_exp_level_lower field
	male_exp_level_lower	If a point exposure level is not reported in the male_exp_level_value field, use this column to enter the reported lower bound of an exposure range
	male_exp_level_upper_sign	Can use < or <= in association with the reported upper bound of an exposure range in the male_exp_level_upper field
	male_exp_level_upper	If a point exposure level is not reported in male_exp_level_value, use this column to enter the reported upper bound of an exposure range
	female_exp_level_value	If a point value is provided for the level of exposure (eg, average amount of cigarettes/day), enter the value here
	female_exp_level_value_type	If a value is entered in 'female_exp_level_value', select the type of measure of the value. For example, if the point value is average cigarettes/day, select 'mean'.
	female_exp_level_lower_sign	Can use > or >= in association the reported lower bound of an exposure range in the female_exp_level_lower field
	female_exp_level_lower	If a point exposure level is not reported in the female_exp_level_value field, use this column to enter the reported lower bound of an exposure range
	female_exp_level_upper_sign	Can use < or <= in association with the reported upper bound of an exposure range in the female_exp_level_upper field
	female_exp_level_upper	If a point exposure level is not reported in female_exp_level_value, use this column to enter the reported upper bound of an exposure range
	exp_freq_lower	If provided, the lower bound of alcohol consumption frequency for the exposed group
	exp_freq_upper	If provided, the upper bound of alcohol consumption frequency for the exposed group

Table S4. Causal criteria extraction template

Category	Variable	Definition
	exp_freq_unit	If provided, the unit of alcohol consumption frequency for the exposed group
	exp_unit	Specify the unit of exposure (eg, grams/day)
	exp_unit_def	Free text field to record an article's conversion factor for alcohol consumption units of the exposed group
Unexposed Measurements	unexp_reference_group_def	Provide a brief description of the unexposed/reference group
	unexp_level_value	If a point value is provided for the level of exposure (eg, average amount of cigarettes/day), enter the value here
	unexp_level_value_type	If a value is entered in 'unexp_level_value', select the type of measure of the value. For example, if the point value is average cigarettes/day, select 'mean'.
	unexp_level_lower_sign	Can use > or >= in association the reported lower bound of an exposure range in the unexp_level_lower field
	unexp_level_lower	If a point exposure level is not reported in the unexp_level_value field, use this column to enter the reported lower bound of an exposure range
	unexp_level_upper_sign	Can use < or <= in association with the reported upper bound of an exposure range in the unexp_level_upper field
	unexp_level_upper	If a point exposure level is not reported in unexp_level_value, use this column to enter the reported upper bound of an exposure range
	male_unexp_level_value	If a point value is provided for the level of exposure (eg, average amount of cigarettes/day), enter the value here
	male_unexp_level_value_type	If a value is entered in 'male_unexp_level_value', select the type of measure of the value. For example, if the point value is average cigarettes/day, select 'mean'.
	male_unexp_level_lower_sign	Can use > or >= in association the reported lower bound of an exposure range in the male_unexp_level_lower field
	male_unexp_level_lower	If a point exposure level is not reported in the male_unexp_level_value field, use this column to enter the reported lower bound of an exposure range
	male_unexp_level_upper_sign	Can use < or <= in association with the reported upper bound of an exposure range in the male_unexp_level_upper field
	male_unexp_level_upper	If a point exposure level is not reported in male_unexp_level_value, use this column to enter the reported upper bound of an exposure range
	female_unexp_level_value	If a point value is provided for the level of exposure (eg, average amount of cigarettes/day), enter the value here
	female_unexp_level_value_type	If a value is entered in 'female_unexp_level_value', select the type of measure of the value. For example, if the point value is average cigarettes/day, select 'mean'.
	female_unexp_level_lower_sign	Can use > or >= in association the reported lower bound of an exposure range in the female_unexp_level_lower field
	female_unexp_level_lower	If a point exposure level is not reported in the female_unexp_level_value field, use this column to enter the reported lower bound of an exposure range
	female_unexp_level_upper_sign	Can use < or <= in association with the reported upper bound of an exposure range in the female_unexp_level_upper field
	female_unexp_level_upper	If a point exposure level is not reported in female_unexp_level_value, use this column to enter the reported upper bound of an exposure range
	unexp_freq_lower	If provided, the lower bound of alcohol consumption frequency for the unexposed group
	unexp_freq_upper	If provided, the upper bound of alcohol consumption frequency for the unexposed group
	unexp_freq_unit	If provided, the unit of alcohol consumption frequency for the unexposed group
	unexp_unit	Specify the unit of exposure (eg, grams/day) for the unexposed group
	unexp_unit_def	Free text field to record an article's conversion factor for alcohol consumption units of the unexposed group
Outcome	outcome	Outcome: Select the outcome
	acause	Auto-populated from outcome
	outcome_components	List specific outcomes that are included in aggregate outcome definitions (eg, pneumonia for the lower respiratory infections outcome). Separate with semi-colons.
	outcome_def	Outcome definition: Provide a brief description of the outcome as reported in the study.
	outcome_type	Outcome type: please specify if the outcome definition included incidence of or mortality from a disease endpoint
	outcome_assess_1	Method of outcome assessment: Specify the method of assessment of the study outcome. If more than 1 are appropriate, enter additional methods in the next column labeled "outcome_assess_2"
	outcome_assess_2	Method of outcome assessment: Specify the method of assessment of the study outcome. If more than 2 are appropriate, enter additional methods in the next column labeled "outcome_assess_3"

Table S4. Causal criteria extraction template

Category	Variable	Definition
	outcome_assess_3	Method of outcome assessment: Specify the method of assessment of the study outcome.
Confounders	confounders_age	if controlled for in the relative risk estimation analysis, mark 1 for yes. Mark 0 for no
	confounders_sex	if controlled for in the relative risk estimation analysis, mark 1 for yes. Mark 0 for no
	confounders_education	if controlled for in the relative risk estimation analysis, mark 1 for yes. Mark 0 for no
	confounders_income	if controlled for in the relative risk estimation analysis, mark 1 for yes. Mark 0 for no
	confounders_smoking	if controlled for in the relative risk estimation analysis, mark 1 for yes. Mark 0 for no
	confounders_alcohol_use*	if controlled for in the relative risk estimation analysis, mark 1 for yes. Mark 0 for no
	confounders_physical_activity	if controlled for in the relative risk estimation analysis, mark 1 for yes. Mark 0 for no
	confounders_dietary_components	if controlled for in the relative risk estimation analysis, mark 1 for yes. Mark 0 for no
	confounders_bmi	if controlled for in the relative risk estimation analysis, mark 1 for yes. Mark 0 for no
	confounders_hypertension	if controlled for in the relative risk estimation analysis, mark 1 for yes. Mark 0 for no
	confounders_diabetes	if controlled for in the relative risk estimation analysis, mark 1 for yes. Mark 0 for no
	confounders_hypercholesterolemia	if controlled for in the relative risk estimation analysis, mark 1 for yes. Mark 0 for no
	confounders_other	For other confounders that not listed, list here
Effect Size	page_num	Page number (where you found effect_size) from literature, or survey question where you found effect size; Use page number(s) of article, not page # of pdf
	table_num	Table/Figure number from literature or survey question. For figures, put F before the figure number. Text may be entered (eg, name of a table).
	in_supplement	Is the effect size found in the supplementary material of the article? 1=yes, 0=no
	measure	The type of effect size being extracted
	effect_size_unit	Mathematical space of the effect size. Rows with 'linear' effect_size_unit values must have positive values for mean. If lower and upper are provided, they must also be positive.
	effect_size_measure	Effect size measure: Specify the measure of effect size
	mean	Effect size estimate: Provide the effect size estimate
	lower	Provide the lower limit of the confidence interval. Enter on a "per 1" basis. (If the CI is reported as a percent, you must convert to a decimal.) These 3 fields must all be filled in if any of them are filled in: lower, upper, uncertainty_type_value.
	upper	Provide the upper limit of the confidence interval. Enter on a "per 1" basis. (If the CI is reported as a percent, you must convert to a decimal.) These 3 fields must all be filled in if any of them are filled in: lower, upper, uncertainty_type_value.
	uncertainty_type_value	This field is required if 'lower' & 'upper' are entered. This column represents the confidence level which is reported at (Eg. 95, 90, 99). These 3 fields must all be filled in if any of them are filled in: lower, upper, uncertainty_type_value.
	standard_error	Required if upper and lower uncertainty bounds are not extracted
	uncertainty_issue	Mark with a 1 if no uncertainty is reported, if some sort of uncertainty is reported, mark 0
	effect_size_derived	Was the effect size calculated (eg, adjusted to linear space, calculated using an online tool)? 1=yes, no=0
	effect_size_from_image	Was WebPlotDigitizer or another image processing tool used to extract the effect size? 1=yes, no=0
	subgroup_analysis	1 if RR is from main analysis (all participants), 0 if sub-analysis (only males, or among a specific age group, etc.)
	subgroup_analysis_free_text	if a sub-analysis, describe it (i.e. age, sex, etc.)
	effect_size_multi_location	1 if the reported effect size is from a multi-country study and only one effect size has been reported for all locations, otherwise 0
	effect_size_multi_location_specify	which geography level is the RR for
	pooled_cohort	1 if the reported effect size is from a pooled analysis and only pooled effect size has been reported, otherwise 0
	dose_response	Does the study support a dose-response relationship between the exposure and the outcome? (1= yes, 0=no)
	most_adj_model	Does this model which produced the effect size have the most number of controlled variables of all the reported models? 1=yes, 0=no
	least_adj_model	Does this model which produced the effect size have the least number of controlled variables of all the reported models? 1=yes, 0=no

Table S4. Causal criteria extraction template

Category	Variable	Definition
	reverse_causation	Is there a high potential for reverse causation, ie, the outcome causing the risk? 1=yes, 0=no
	reverse_causation_assess	Enter a semi-colon-separted list of methods employed by researchers to control reverse causation. If no methods were utilized, enter 'none'.
Cohort studies	cohort_person_years_exp	Please specify the person years of follow up in the exposed group
	cohort_person_years_unexp	Please specify the person years of follow up in the unexposed group
	cohort_person_years_total	Enter the total person-years of follow-up if person-years of follow up in exposed and unexposed not reported
	cohort_number_events_exp	Please specify the number of events in the exposed group
	cohort_number_events_unexp	Please specify the number of events in the unexposed group
	cohort_number_events_total	Enter the total number of events/cases if number of events in exposed and unexpoxed not reported
	cohort_sample_size_exp	Please specify the number of people in the exposed group if person-years of follow up in exposed not reported
	cohort_sample_size_unexp	Please specify the number of people in the unexposed group if person-years of follow up in unexposed not reported
	cohort_sample_size_total	Please specify the number of people included in the analysis if total person-years of follow up in not reported
Case-control studies	cc_community	Were the controls selected from the community? 1 = yes, 0 = no
	cc_community_type	Select the type of community control
	cc_cases_exp	Number of cases in the exposed category
	cc_controls_exp	Number of controls in the exposed category
	cc_cases_unexp	Number of cases in the unexposed category
	cc_controls_unexp	Number of controls in the unexposed category
	cc_cases_total	Number of cases (across all exposure levels used in the estimate of the effect size)
	cc_controls_total	Number of controls (across all exposure levels used in the estimate of the effect size)
Other	note_modeler	for modelers only, audience is modeler, not for correspondence
	note_sr	notes related to extraction, including assumptions, data adjustment, problems with source, any other notes that may be relevant, etc.
	group_review	Indicate whether data should be visible (=1) or hidden (=0) for modeling purposes. If data are hidden, they have no influence on modeling whatsoever.
	bundle_name	Best practices are to enter bundle_name. You must enter bundle_name if you're extracting more than one bundle into the template; without this, you won't be able to split your sheets by bundle for upload.
	input_type	IGNORE DURING EXTRACTION. Options are: extracted, adjusted, split, or collapsed.
	is_outlier	IGNORE DURING EXTRACTION. 0 = not outliered, 1 = outlier; will be 0 for all rows after initial upload.
	extractor	uwnet id of person who extracted the data

Note. * This column refers to characteristics of participants' alcohol consumption other than average alcohol intake (e.g., binge drinking).
CI = confidence interval, GBD = Global Burden of Diseases, Injuries, and Risk Factors Study, IHME = Institute for Health Metrics and Evaluation, RR = relative risk.

Section 4: Study characteristics

Table S5. Study characteristics of included conventional observational studies

Author	Year	Study name	Population	Location	Study design	Sex	Follow-up	Age start	Age end	Exposure assessment	Endpoint	Disease ascertainment	Person-years	Events	Sample size	Cases	Controls	Control pool	Exposed
Albert	1999	Physician's Health Study	US male physicians	United States of America	Prospective cohort	Males	12 years	40	84	Self-report	Mortality	Self-report; Medical records/disease registries	258,444	141	21,537	not provided	not provided	not provided	not provided
Arriola	2010	EPIC Study	Residents of the Asturias, San Sebastian, Navarra, Granada, and Murcia regions of Spain	Spain	Prospective cohort	Both	3 years	29	69	Self-report	Incidence & Mortality	Self-report; Medical records/disease registries; death certificates	160,024	609	41,245	not provided	not provided	not provided	not provided
Au Yeung	2013	Guangzhou Biobank Cohort Study	Residents aged 50+	China	Prospective cohort	Males	not provided	50	99	Self-report	Incidence	not provided	not provided	not provided	4,867	234	not provided	not provided	2,482
Augustin	2004	not provided	Patients in greater Milan area with first episode of nonfatal acute MI	Italy	Case-control	Both	not provided	25	79	Self-report	Morbidity	Self-report; Hospital registry; Medical records/disease registries	not provided	not provided	985	507	478	Community	698
Bazzano	2009	China National Hypertension Survey Epidemiology Follow-up Study	Nationally representative population of men from 15 provinces of China	China	Prospective cohort	Males	8 years	40	99	Self-report	Mortality, Incidence	Self-report; Medical records/disease registries; Death certificates	494,084	725	64,579	not provided	not provided	not provided	not provided
Bell	2017	CALIBER programme	Patients in the UK aged >= 30 between 1 Jan. 1997 and 25 Mar. 2010 with no record indicating any CVD before study entry	United Kingdom	Prospective cohort	Both	6 years	30	99	Self-report	Incidence & Mortality	Medical records/disease registries; Self-report	11,637,926	21,754	1,937,360	not provided	not provided	not provided	not provided
Bergmann	2013	EPIC Study	The EPIC population consists of sub-cohorts recruited in 10 European countries	Denmark, France, Germany, Greece, Italy, Netherlands, Spain, England	Prospective cohort	Both	3 years	25	70	Self-report	Mortality	Medical records/disease registries; Death certificates	1,323,357	26,411	380,395	not provided	not provided	not provided	not provided
Beulens	2007	Health Professional's Follow-Up Study	Male health professionals with hypertension	United States of America	Prospective cohort	Males	16 years	40	75	Self-report	Mortality	Medical records/disease registries	187,376	279	11,711	not provided	not provided	not provided	not provided
Bianchi	1993	not provided	Women with acute MI admitted to	Italy	Case-control	Females	not provided	18	74	Self-report	Morbidity	Medical records/disease	not provided	not provided	983	298	685	Community (age-matched;	569

Table S5. Study characteristics of included conventional observational studies

Author	Year	Study name	Population	Location	Study design	Sex	Follow-up	Age start	Age end	Exposure assessment	Endpoint	Disease ascertainment	Person-years	Events	Sample size	Cases	Controls	Control pool	Exposed
			coronary care units of 30 hospitals in northern Italy									registries; Self-report						admitted to hospital for other issues)	
Bobak	2016	Health, Alcohol and Psychosocial factors in Eastern Europe (HAPIEE) study	Random population sample recruited	Czechia, Lithuania, Poland, Russian Federation	Prospective cohort	Both	6.9 years	45	72	Self-report	Mortality	Medical records/disease registries; Death certificates	236,698	672	34,304	not provided	not provided	not provided	not provided
Boffetta	1990	American Cancer Society prospective study	White male ACS volunteers aged 40-49	United States of America	Prospective cohort	Males	12 years	40	59	Self-report	Mortality	Medical records/disease registries; Death certificates	3,321,624	18,771	276,802	not provided	not provided	not provided	not provided
Brenner	2001	not provided	Patients with clinically stable, angiographically-confirmed coronary heart disease	Germany	Case-control	Both	not provided	40	68	Self-report	Morbidity	Biomarker	not provided	not provided	791	312	479	Communit y (blood donors recruited from local Red Cross center)	614
Britton	2004	Whitehall II study	Male civil servants	United Kingdom	Prospective cohort	Males	15 years	35	55	Self-report	Incidence & Mortality	Medical records/disease registries	78,165	356	5,211	not provided	not provided	not provided	not provided
Camargo	1997	Physician's Health Study	US male physicians	United States of America	Prospective cohort	Males	11 years	40	84	Self-report	Incidence & Mortality	Self-report; Medical records and disease registries	236,830	690	21,530	not provided	not provided	not provided	not provided
Chang	2020	not provided	patients of the Korean National Health Insurance service between 2007 and 2014	Republic of Korea	Prospective cohort	Both	2 years	30	84	Self-report	Incidence & Mortality	Administrative medical records	331,669	2,583	112,403	312	2,271	Non-drinkers within the cohort profile	15,687
Chiuve	2010	Nurses' Health Study	US registered female nurses	United States of America	Prospective cohort	Females	26 years	30	55	Self-report	Incidence & Mortality	Death certificates; Medical records and disease registries	3,164,200	3,182	121,700	not provided	not provided	not provided	not provided
Cho	2015	Korean Genome and Epidemiology Study (KoGES)	Subjects recruited from Ansung-Ansan cohorts	Republic of Korea	Prospective cohort	Both	not provided	39	70	Self-report	Incidence	Physician diagnosis; Self-report	not provided	122	7,152	not provided	not provided	not provided	3,387
Colditz	1985	not provided	Massachusetts residents aged 66 and older identified from a statewide area probability sample	United States of America	Prospective cohort	Both	4.75	66	99	Self-report	Mortality	Death certificates	5,823.5	317	1,226	not provided	not provided	not provided	not provided

Table S5. Study characteristics of included conventional observational studies

Author	Year	Study name	Population	Location	Study design	Sex	Follow-up	Age start	Age end	Exposure assessment	Endpoint	Disease ascertainment	Person-years	Events	Sample size	Cases	Controls	Control pool	Exposed
Dai	2015	National Heart, Lung, and Blood Institute (NHLBI) Twin Study	Middle-aged white veteran male twins	United States of America	Prospective cohort	Males	41 years	42	52	Self-report	Mortality	Medical records/disease registries; Death certificates	42,148	129	1,028	not provided	not provided	not provided	not provided
Dam	2016	Diet, Cancer, and Health study	Postmenopausal women born in Denmark	Denmark	Prospective cohort	Females	11 years	50	64	Self-report	Incidence & Mortality	Medical records/disease registries	236,753	1,750	21,523	not provided	not provided	not provided	not provided
Degerud	2021	Finnmark III; Cohort of Norway; Twin panel II	Current drinkers with no history of major IHD events or stroke from Norwegian population-based health surveys and a survey from the Norwegian Twin Registry.	Norway	Retrospective cohort	Both	9 years	19	89	Self-report	Incidence & Mortality	Medical records/disease registries; Death certificates	400,284	1,535	44,476	not provided	not provided	not provided	not provided
de Labry	1992	not provided	Males that completed an alcohol consumption survey in 1973	United States of America	Prospective cohort	Males	12 years	18	99	Self-report	Mortality	Medical Records, death certificates	21,716	159	1,823	54	not provided	cohort sample	not provided
Doll	2005	not provided	Male British doctors	United Kingdom	Prospective cohort	Males	23 years	48	78	Self-report	Mortality	Death certificates; Medical records/disease registries	283,475	1,819	12,325	not provided	not provided	not provided	not provided
Dorn	2007	not provided	Incident MI cases recruited from Western NY hospitals	United States of America	Case-control	Females	not provided	35	69	Self-report	Morbidity	Medical records/disease registries; Physician diagnosis	not provided	not provided	1,885	320	1,565	Community (Identified from motor vehicle rolls and HCFA files)	947
Dyer	1980	Chicago Western Electric Company study	Men employed by the Hawthorne Works of the Western Electric Company	United States of America	Prospective cohort	Males	17 years	40	55	Self-report	Mortality	Death certificates; Medical records/disease registries	31,144	149	1,832	not provided	not provided	not provided	not provided
Ebbert	2005	Iowa Women's Heatlh Study (IWHS)	Women with a valid Iowa driver's license in 1985	United States of America	Prospective cohort	Females	14 years	55	69	Self-report	Mortality	Death certificates; Medical records/disease registries	404,377	757	41,836	not provided	not provided	not provided	13,827
Ebrahim	2008	British Women's Heart and Health Study and	BWHHS: Women aged 50-79 selected from 23 British towns;	United Kingdom	Prospective cohorts	Both	20 years	45	79	Self-report	Incidence & Mortality	Medical records/disease registries; Self-report	29,498.7	283	4,525	not provided	not provided	not provided	not provided

Table S5. Study characteristics of included conventional observational studies

Author	Year	Study name	Population	Location	Study design	Sex	Follow-up	Age start	Age end	Exposure assessment	Endpoint	Disease ascertainment	Person-years	Events	Sample size	Cases	Controls	Control pool	Exposed
		Caerphilly study	Caerphilly: Men recruited from electoral roles in Caerphilly, South Wales																
Fan	2019	NESARC-III	Cases were NESARC-III respondents with doctor-ascertained CHD	United States of America	Case-control	Both	not provided	18	99	Self-report	Morbidity	Physician diagnosis; Medical records/disease registries	not provided	not provided	19,300	1,671	17,629	Community (NESARC-III respondents free of CHD and other alcohol-related conditions)	8,006
Friedman	1986	Framingham Heart Study	Residents of Framingham, MA	United States of America	Prospective cohort	Both	24 years	30	59	Self-report	Mortality	Medical records/disease registries; Death certificates	113,880	2,414	4,745	not provided	not provided	not provided	1,560
Fuchs	2004	Atherosclerosis Risk in Communities (ARIC) study	Equal numbers of participants were selected from Forsyth County, North Carolina; Jackson, Mississippi; selected suburbs of Minneapolis, Minnesota; and Washington County, Maryland	United States of America	Prospective cohort	Both	9.8 years	45	64	Self-report	Incidence & Mortality	Self-report; Medical records/disease registries	142,158.8	707	14,506	not provided	not provided	not provided	10,791
Fumeron	1995	ECTIM study	Cases recruited from WHO-MONICA registers from Belfast, Lille, Strasbourg, and Toulouse	France, Northern Ireland	Case-control	Males	not provided	25	64	Self-report	Morbidity	Physician diagnosis; Medical records/disease registries	not provided	not provided	1,332	608	724	Community (age- and sex-matched controls recruited from same areas)	1,145
Garfinkel	1988	American Cancer Society study	Women over 30 in the US	United States of America	Prospective cohort	Females	12 years	30	99	Self-report	Mortality	Medical records/disease registries; Death certificates	6,139,265	17,349	581,321	not provided	not provided	not provided	113,939
Gaziano	1993	not provided	Patients admitted to the coronary care units of 6	United States of America	Case-control	Both	not provided	18	76	Self-report	Morbidity	Physician diagnosis; Medical	not provided	not provided	680	340	340	Community (location-matched)	not provided

Table S5. Study characteristics of included conventional observational studies

Author	Year	Study name	Population	Location	Study design	Sex	Follow-up	Age start	Age end	Exposure assessment	Endpoint	Disease ascertainment	Person-years	Events	Sample size	Cases	Controls	Control pool	Exposed
			suburban Boston hospitals									records/disease registries							
Gémes	2016	HUNT 2 study	All adults aged 20 and older residing in Nord-Trøndelag County, Norway	Norway	Prospective cohort	Both	11.6 years	20	99	Self-report	Incidence & Mortality	Medical records/disease registries; Death certificates	682,393.2	2,966	58,827	not provided	not provided	not provided	not provided
Genchev	2001	not provided	Cases were admissions to the cardiology unit, Central Clincal Hospital, Sofia	Bulgaria	Case-control	Both	not provided	45	69	Self-report	Morbidity	Physician diagnosis; Medical records/disease registries	not provided	not provided	309	155	154	Community (concurrent admissions for minor elective surgery)	164
Gigleux	2006	Quebec Cardiovascular Study	Random sample of French Canadian men from 7 towns in the Quebec City metropolitan area	Canada	Prospective cohort	Males	13 years	35	64	Self-report	Incidence & Mortality	Medical records/disease registries; Self-report; Death certificates	25,558	219	1,966	not provided	not provided	not provided	1,484
Goldberg	1994	Honolulu Heart Program	Men of Japanese ancestry born between 1900 and 1919 residing on Oahu in 1965	United States of America	Prospective cohort	Males	15 years	55	64	Self-report	Incidence & Mortality	Death certificates; Medical records and disease registries	91,035	132	6,069	not provided	not provided	not provided	2,362
Goldberg	1995	Honolulu Heart Program	Men of Japanese ancestry born between 1900 and 1919 residing on Oahu in 1965	United States of America	Prospective cohort	Males	20 years	55	64	Self-report	Incidence & Mortality	Death certificates; Medical records and disease registries	54,200	352	2,710	not provided	not provided	not provided	not provided
Gordon	1985	Albany Study	Male civil service employees in New York state	United States of America	Prospective cohort	Males	28 years	38	55	Self-report	Incidence & Mortality	Physician diagnosis; Medical records/disease registries; Death certificates	47,824	507	1,708	not provided	not provided	not provided	not provided
Gun	2006	not provided	Male employees of Australian Institute of Petroleum	Australia	Prospective cohort	Males	20 years	15	99	Self-report	Mortality	Medical records/disease registries	330,940	295	16,547	not provided	not provided	not provided	not provided
Hammar	1997	not provided	Individuals in the Swedish Twin Registry	Sweden	Nested case-control	Males	not provided	18	75	Self-report	Morbidity & Mortality	Physician diagnosis; Medical records/disease registries	not provided	not provided	2,329	429	1,900	Community (selected from study base)	204
Harriss	2007	Melbourne Collaborative Cohort Study	Residents of Melbourne metropolitan area	Australia	Prospective cohort	Both	11.4 years	40	69	Self-report	Mortality	Medical records/disease registries;	435,480	275	38,200	not provided	not provided	not provided	not provided

Table S5. Study characteristics of included conventional observational studies

Author	Year	Study name	Population	Location	Study design	Sex	Follow-up	Age start	Age end	Exposure assessment	Endpoint	Disease ascertainment	Person-years	Events	Sample size	Cases	Controls	Control pool	Exposed
												Death certificates							
Hart	2008	Midspan Collaborative cohort study	Participants from 27 workplaces in Glasgow, Clydebank, and Grangemouth	Scotland	Prospective cohort	Males	29 years	21	64	Self-report	Mortality & Incidence	Medical records/disease registries; Death certificates	174,000	1,217	6,000	not provided	not provided	not provided	4,114
Henderson	2007	Multiethnic Cohort	Men and women in Hawaii and California	United States of America	Prospective cohort	Both	10 years	45	75	Self-report	Mortality	Death certificates	1,394,060	572	139,406	not provided	not provided	not provided	not provided
Hines	2001	Physician's Health Study	US Male Physicians with newly-diagnosed cases of MI	United States of America	Case-control	Males	not provided	40	84	Self-report	Morbidity	Self-report; Medical records/disease registries	not provided	not provided	1,166	396	770	Communit y (drawn from th same cohort)	846
Hippe	1999	The Copenhagen City Heart Study; The Copenhagen Male Study; and the Glostrup Population Studies	Danish population	Denmark	Prospective cohort	Both	12.3 years	20	93	Self-report	Incidence & Mortality	Medical records/disease registries; Death certificates	303,367.2	1,304	24,664	not provided	not provided	not provided	not provided
Ikehara	2008	The Japanese Collaborative Cohort Study	People living in 45 communities across Japan	Japan	Prospective cohort	Both	14.2 years	40	79	Self-report	Mortality	Death certificates	1,065,295	736	83,682	not provided	not provided	not provided	35,035
Ikehara	2009	The Japan Public Health Center (JPHC) Study - cohort II	Residents of 5 PHC areas	Japan	Prospective cohort	Males	9.9 years	40	69	Self-report	Mortality	Medical records/disease registries; Death certificates	191,624.4	207	19,356	not provided	not provided	not provided	15,229
Ilic	2018	not provided	Cases were newly diagnosed MI patients in Kragujevac	Serbia	Case-control	Both	not provided	18	99	Self-report	Morbidity	Physician diagnosis; Medical records/disease registries	not provided	not provided	374	187	187	Communit y (matched by age, gender, and place of residence)	196
Iso	1995	not provided	Men free of a history of stroke and CHD in three rural communities in Japan	Japan	Prospective cohort	Males	10.5 years	40	69	Self-report	Incidence & Mortality	National insurance claims; Medical records/disease registries; Death certificates	30,345	34	2,890	not provided	not provided	not provided	2,305
Jackson	1991	MONICA	White Residents of Auckland, New Zealand 25-64 in 1986-1988	New Zealand	Case-control	Both	not provided	25	64	Self-report	Morbidity	Induction into the MONICA register	not provided	not provided	1,867	457	1,410	Communit y	810

Table S5. Study characteristics of included conventional observational studies

Author	Year	Study name	Population	Location	Study design	Sex	Follow-up	Age start	Age end	Exposure assessment	Endpoint	Disease ascertainment	Person-years	Events	Sample size	Cases	Controls	Control pool	Exposed
Jakovljevic	2004	not provided	Adults aged 30-60 without chronic disease who agreed to participate	Serbia	Prospective cohort	Both	20 years	30	60	Self-report	Mortality	Death certificates	not provided	80	286	146	140	Non-drinkers within the cohort profile	286
Kabagambe	2005	not provided	Hispanic adult americans living in the central valley of Costa Rica from 1994 to 2004 presenting to a participating hospital with myocardial infarction	Costa Rica	Case-control	Both	2 weeks	0	99	Self-report	Morbidity	Hospital record	not provided	not provided	4,180	2,090	2,090	Community	3,409
Kalandidi	1992	not provided	Patients diagnosed with CHD at the Hippokrateion Hospital from 1990 to 1991	Greece	Case-control	Both	not provided	30	90	Administered interview	Morbidity	Hospital record	not provided	not provided	899	329	570	Hospital patients with non-CHD diseases	not provided
Kaufman	1985	not provided	Men 30-54 residents of the northeastern US who were hospitalized for first myocardial infarction between 1980 to 1983	United States of America	Case-control	Males	not provided	30	54	Administered interview	Morbidity	Hospital record	not provided	not provided	3,151	2,170	981	Hospital controls with a primary diagnosis not alcohol related	3,037
Kawanishi	1990	not provided	Males over the age of 44 diagnosed with CHD	Japan	Case-control	Males	not provided	44	99	Self-report	Morbidity	Hospital diagnosis	not provided	not provided	197	44	153	University employees	84
Keil	1997	MONICA	Participants of the MONICA cohort aged 45-64	Germany	Prospective cohort	Males	8 years	45	64	Self-report	Incidence & Mortality	Physical examination	15,340	92	2,041	not provided	not provided	not provided	1,491
Key	2009	EPIC-Oxford	Adult participants of the EPIC-Oxford Cohort aged 35-69 recruited between 1993 to 1999	United Kingdom	Prospective cohort	Both	10 years	35	69	Self-report	Mortality	Death certificates	not provided	742	64,234	not provided	not provided	not provided	39,240
Kitamura	1998	not provided	Male workers in Osaka, Japan working at 1 of	Japan	Prospective cohort	Males	not provided	40	59	Self-report	Incidence & Mortality	Death certificates; Insurance	not provided	169	8,476	7,003	1,260	Participants without CVD	not provided

Table S5. Study characteristics of included conventional observational studies

Author	Year	Study name	Population	Location	Study design	Sex	Follow-up	Age start	Age end	Exposure assessment	Endpoint	Disease ascertainment	Person-years	Events	Sample size	Cases	Controls	Control pool	Exposed
			13 industrial companies that participated in CVD risk surveys between 1975 and 1954									claims; Absenteeism reports due to sickness; Annual risk factor surveys							
Kivelä	1989	not provided	Rural Finnish men aged 55-74 first examined in 1959	Finland	Prospective cohort	Males	10 years	55	74	Self-report	Mortality	Death certificates	not provided	241	2,403	not provided	297	not provided	2,006
Klatsky	2005	not provided	Participants of prepaid healthcare program in San Fransisco and Oakland, CA from 1978 to 1985	United States of America	Prospective cohort	Both	not provided	30	70	Self-report	Incidence	Administrative medical records	1,820,200	1,559	126,235	not provided	15,079	not provided	112,156
Kono	1986	not provided	Japanese physicians participating in the 1965 cohort study	Japan	Prospective cohort	Males	not provided	20	99	Self-report	Mortality	Death certificataes	not provided	1,283	5,135	not provided	1,074	Non-drinkers	4,061
Kono	1991	not provided	Adults aged 40-69 ddmitted for first AMI at Fukuoka University Hospital or Fukuoka City Hospital from 1988 to 1990	Japan	Case-control	Males	not provided	40	69	Self-report	Morbidity	Hospital records	not provided	not provided	592	116	476	Communit y	269
Kunutsor	2021	PREVEND	Representative sample of inhabitants living in Groningen	Netherlands	Prospective cohort	Both	8.3 years	28	75	Self-report	Incidence & Mortality	PRISMANT (Dutch national registry of hospital discharge diagnoses)	43,209.8	326	5,206	not provided	not provided	not provided	3,928
Kurl	2021	Kuopio Ischemic Heart Disease study (KIHD)	Men aged 42-61 at recruitment with no history of CVD	Finland	Prospective cohort	Males	28 years	42	61	Self-report	Incidence & Mortality	Statistics Finland; Medical records/disease registries	45,416	196	1,622	not provided	not provided	not provided	not provided
Larsson	2017	COSM and SMC cohorts	Adult participants of two prospective cohorts from Sweden from 1997	Sweden	Prospective cohort	Both	12 years	45	83	Self-report	Incidence & Mortality	Administrative medical records	883,553	5,178	74,612	not provided	5,970	Non-drinkers	68,642
Lazarus	1991	not provided	Adult residents of Alameda	United States of America	Prospective cohort	Both	9 years	16	99	Self-report	Mortality	Death certificates	not provided	591	4,070	not provided	830	Non-drinkers	3,240

Table S5. Study characteristics of included conventional observational studies

Author	Year	Study name	Population	Location	Study design	Sex	Follow-up	Age start	Age end	Exposure assessment	Endpoint	Disease ascertainment	Person-years	Events	Sample size	Cases	Controls	Control pool	Exposed
			county, CA in 1965																
Lee	2004	Iowa Women's Health Study	Postmenopausal women aged 55-69 participating in the Iowa Women's Health Study cohort in 1986	United States of America	Prospective cohort	Females	11 years	55	69	Self-report	Incidence	Self-report; Death certificates	332,858	1,922	35,698	753	1,168	Non-drinkers	
Liao	2000	National Health Interview Surveys	Adult participants of the National Health Interview Survey in 1988 and 1990	United States of America	Prospective cohort	Both	6 years	40	99	Self-report	Mortality	Death certificates	not provided	5,540	43,695	not provided	1,720	Non-drinkers	13,186
Licaj	2016	Swedish Women's Lifestyle and Health cohort	Women aged 30-49 randomly selected to participate in the WLH cohort study from the Uppsala Healthcare Region in Sweden from 1991-1992	Sweden	Prospective cohort	Females	21 years	30	49	Self-report	Mortality	Death certificates	900,000	2,100	48,249	not provided	6,587	Non-drinkers	41,662
Lindschou	2011	Diet, Cancer, and Health study	Danish adults aged 50-64 born in denmark without previous cancers	Denmark	Prospective cohort	Both	not provided	50	64	Self-report	Incidence & Mortality	Administrative medical records	422,171	1,131	57,053	not provided	5,927	Non-drinkers	49,535
Makelä	2005	The Finnish Drinking Habit Surveys	Participants in the Finnish Drinking Habit Surveys in 1969, 1976, and 1984	Finland	Prospective cohort	Both	16.3 years	25	69	Administered interview	Incidence & Mortality	Death register	not provided	1,144	6,394	not provided	1,275	Non-drinkers	5,119
Malyutina	2002	MONICA	Male residents of Novosibirsk, Russia who participated in the WHO MONICA cohort study from 1985 to 1995	Siberia	Prospective cohort	Males	9.5 years	25	64	Self-report	Mortality	Death certificate	55,195.2	815	6,502	not provided	776	Non-drinkers	5,594
Maraldi	2006	Health, Aging, Body Composition Study	participants aged 70-79 recruited between 1997 to 1998 of the health aging and body composition cohort study	United States of America	Prospective cohort	Both	5.6 years	0	99	Self-report	Incidence & Mortality	Death certificate	not provided	397	2,487	not provided	1,221	Non-drinkers	1,226

Table S5. Study characteristics of included conventional observational studies

Author	Year	Study name	Population	Location	Study design	Sex	Follow-up	Age start	Age end	Exposure assessment	Endpoint	Disease ascertainment	Person-years	Events	Sample size	Cases	Controls	Control pool	Exposed
Marques-Vidal	2004	PRIME study	Middle-aged men in Lille, Strasbourg, Toulouse, and Belfast	France	Prospective cohort	Males	5+ years	50	59	Self-report	Incidence & Mortality	Physician diagnosis	not provided	200	9,750	not provided	693	Non-drinkers	6,659
Mehlig	2014	INTERGENE case-control study	Cases are patients admitted to 3 regional hospitals and diagnosed with MI	Sweden	Case-control	Both	not provided	25	74	Self-report	Morbidity	Physician diagnosis	not provided	not provided	3,539	618	2,921	Community (age- and location-matched)	3,163
Meisinger	2006	MONICA Augsberg survey	Residents of Augsburg aged 25-64 between 1984-1985	Germany	Prospective cohort	Males	15.7 years	25	64	Self-report	Incidence & Mortality	Administrative medical records; Disease registries	29,484.6	150	1,878	not provided	not provided	not provided	1,639
Merry	2011	CAREMA study	Adults aged 20-59 at baseline and randomly sampled from the Maastricht region	Netherlands	Prospective cohort	Both	11.1 years	20	59	Self-report	Incidence & Mortality	Administrative medical records; Disease registries	211,965.6	420	19,096	not provided	not provided	not provided	16,705
Miller	1990	not provided	Trinidadian men aged 35-69 between 1977 and 1986	Trinidad and Tobago	Prospective cohort	Males	7.5 years	35	69	Self-report	Incidence & Mortality	Administrative medical records/disease registries; Death certificates	10,057.5	49	1,341	not provided	not provided	not provided	924
Millwood	2019	China Kadoorie Biobank cohort	Adults from 10 diverse rural and urban areas of China	China	Prospective cohort	Males	10 years	35	74	Self-report	Incidence & Mortality	Death certificates	2,102,050	5,676	210,205	not provided	not provided	not provided	69,897
Miyake	2000	Fukuoka Heart Study Group	Middle-aged Japanese patients admitted to hospital for first AMI	Japan	Case-control	Both	not provided	40	79	Self-report	Morbidity	Physician diagnosis	not provided	not provided	1,040	384	656	Community (residents of same municipality)	858
Mukamal	2006	Health Professional's Follow-up Study	US male health professionals	United States of America	Prospective cohort	Males	16 years	40	75	Self-report	Incidence & Mortality	Self-report; Administrative medical records/disease registries	141,872	106	8,867	not provided	not provided	not provided	6,978
Ng	2020	Canadian Community Health Survey	Canadian residents aged 20+ with no history of chronic disease	Canada	Prospective cohort	Both	15 years	20	99	Self-report	Incidence & Mortality	Administrative medical records/disease registries	1,693,050	17,043	112,870	not provided	not provided	not provided	52,574
Oliveira	2009a	not provided	Portugese caucasian women aged 18+, admitted	Portugal	Case-control	Females	not provided	18	99	Self-report	Morbidity	Physician diagnosis	not provided	not provided	1,671	222	1,449	Community (non-institutionalized	875

Table S5. Study characteristics of included conventional observational studies

Author	Year	Study name	Population	Location	Study design	Sex	Follow-up	Age start	Age end	Exposure assessment	Endpoint	Disease ascertainment	Person-years	Events	Sample size	Cases	Controls	Control pool	Exposed
			with incident AMI in Porto															inhabitants of Porto)	
Oliveira	2009b	not provided	Cases were male patients admitted with incident AMI in Porto	Portugal	Case-control	Males	not provided	18	99	Self-report	Morbidity	Physician diagnosis	not provided	not provided	1,489	638	851	Community (men from the non-institutionalized population of Porto)	1,299
Onat	2009	Turkish Adult Risk Factor (TARF)	Random sample of Turkish adult population	Turkey	Prospective cohort	Both	7.4 years	28	99	Self-report	Incidence	Administrative medical records/disease registries	25,478.2	433	3,443	not provided	not provided	not provided	670
Pedersen	2008	Copenhagen City Heart Study	Men and Women in Copenhagen aged 20+ in 1976-1978	Denmark	Prospective cohort	Both	20 years	20	99	Self-report	Mortality	Danish civil registration system	238,280	1,242	11,914	not provided	not provided	not provided	8,265
Reddiess	2021	BEAT-AF and Swiss-AF	Adults with a prior documentation of AF in Switzerland	Switzerland	Prospective cohort	Both	3 years	45	99	Self-report	Incidence	Self-report; Administrative medical records/disease registries	11,556	95	3,852	not provided	not provided	not provided	3,163
Rehm	1997	NHANES Epidemiologic Follow-up Study (NHEFS)	European-American adults	United States of America	Prospective cohort	Both	14.6 years	40	75	Self-report	Mortality	Administrative medical records/disease registries; Death certificates	99,104.8	552	6,788	not provided	not provided	not provided	not provided
Renaud	1998	not provided	Middle-aged native French men	France	Prospective cohort	Males	12.3 years	40	60	Self-report	Mortality	Death certificates; Administrative medical records/disease registries	418,068	284	34,014	not provided	not provided	not provided	30,266
Ricci	2018	EPIC-CVD study	Adults from 23 centers in 10 countries in Europe	Italy, Spain, Greece, Germany, Denmark, United Kingdom, Netherlands, Sweden	Prospective cohort	Both	12.5 years	35	70	Self-report	Incidence & Mortality	Self-report; Administrative medical records/disease registries; Death certificates	406,862.5	11,006	32,549	not provided	not provided	not provided	not provided
Rimm	1991	Health Professionals Follow-up Study	US male health professionals	United States of America	Prospective cohort	Males	2 years	40	75	Self-report	Mortality	Death certificates; Medical records and disease registries	88,118	350	44,059	not provided	not provided	not provided	33,757
Roerecke	2011	not provided	US residents	United States of America	Prospective cohort	Both	15.1 years	21	99	Self-report	Mortality	National Death Index	150,139.3	326	9,943	not provided	not provided	not provided	9,326

Table S5. Study characteristics of included conventional observational studies

Author	Year	Study name	Population	Location	Study design	Sex	Follow-up	Age start	Age end	Exposure assessment	Endpoint	Disease ascertainment	Person-years	Events	Sample size	Cases	Controls	Control pool	Exposed
Romelsjö	2003	SHEEP case-control study	Cases were patients admitted to hospital with first MI	Sweden	Case-control	Both	not provided	45	70	Self-report	Morbidity	not provided	not provided	not provided	3,800	1,566	2,234	Community (selected from SHEEP study base)	3,772
Romelsjö	2012	not provided	Swedish men up to age 55	Sweden	Prospective cohort	Males	35 years	18	55	Self-report	Incidence & Mortality	National Cause of Death register; National Sweish inpatient register	1,729,385	not provided	3,965	1,566	not provided	not provided	46,354
Rostron	2012	NHIS-LMF	US civilian adults	United States of America	Prospective cohort	Both	2 years	18	99	Self-report	Mortality	Medical records/disease registries	475,718	3,516	237,859	not provided	not provided	not provided	181,344
Ruidavets	2010	PRIME	Men aged 50-59 free of IHD at baseline	France, Northern Ireland	Prospective cohort	Males	10 years	50	59	Self-report	Incidence & Mortality	Medical records/disease registries	97,780	322	9,778	not provided	not provided	not provided	
Schooling	2008	not provided	Hong Kong residents 65+	Special Administrative Region of China	Prospective cohort	Males	4.2 years	65	99	Self-report	Mortality	Death register; Special outpatient and hospitalization databases	227,178	406	54,090	not provided	not provided	not provided	14,150
Schröder	2007	REGICOR project	Cases were patients hospitalized for first MI	Spain	Case-control	Males	not provided	25	74	Self-report	Morbidity	not provided	not provided	not provided	1,514	244	1,270	Community (same region)	1,419
Schutte	2022	UK Biobank Cohort	Participants from one of 22 assessment centers across the UK	United Kingdom	Prospective cohort	Both	6.9 years	40	69	Self-report	Incidence & Mortality	Health and Social Care Information Centre; Information Services Department	2,449,286.1	3,640	354,969	not provided	not provided	not provided	333,259
Scragg	1987	not provided	Residents of the Central Auckland Statistical Area - Cases are MI patients	New Zealand	Case-control	Both	not provided	35	64	Self-report	Morbidity	Medical records/disease registries	not provided	not provided	2,321	735	1,586	Community (location-based selection)	1,885
Sempos	2002	NHANES Epidemiological Follow Up Study (NHEFS)	Black and white US residents aged 40+	United States of America	Prospective cohort	Both	11.7 years	40	74	Self-report	Incidence & Mortality	Hospital discharge records; Death certificates	90,850.5	277	7,765	not provided	not provided	not provided	not provided
Shaper	1994	British Regional Heart Study	Middle-aged men from 24 towns in England, Wales, and Scotland	England, Scotland, Wales	Prospective cohort	Males	9.5 years	40	59	Self-report	Mortality	Self-report; National Health Service registries	73,482.5	611	7,735	not provided	not provided	not provided	7,323

Table S5. Study characteristics of included conventional observational studies

Author	Year	Study name	Population	Location	Study design	Sex	Follow-up	Age start	Age end	Exposure assessment	Endpoint	Disease ascertainment	Person-years	Events	Sample size	Cases	Controls	Control pool	Exposed
Simons	1997	Dubbo study	Non-institutionalized Australian elderly aged 60+ in Dubbo, NSW	Australia	Prospective cohort	Both	6.42 years	60	99	Self-report	Incidence & Mortality	Hospital discharge records; Death certificates	18,008	602	2,805	not provided	not provided	not provided	1,794
Skov-Ettrup	2011	Danish National Cohort Study (DANCOS)	Danish population aged 16+	Denmark	Prospective cohort	Both	6.9 years	16	99	Self-report	Incidence & Mortality	Medical records/disease registries	184823.4	1,136	26,786	not provided	not provided	not provided	21,077
Snow	2009	not provided	Residents in Winnipeg, Manitoba, Canada	Canada	Prospective cohort	Both	10 years	18	64	Self-report	Incidence & Mortality	Medical records/disease registries	11,540	104	1,154	not provided	not provided	not provided	not provided
Song	2018	The Million Veteran Program (MVP)	US veterans	United States of America	Prospective cohort	Both	2.9 years	18	99	Self-report	Incidence & Mortality	Electronic medical records	454,511.2	6,153	156,728	not provided	not provided	not provided	144,857
Streppel	2009	The Zutphen Study	Middle-aged men in Zutphen	Netherlands	Prospective cohort	Males	40 years	40	60	Self-report	Mortality	Death certificates	54,920	348	1,373	not provided	not provided	not provided	not provided
Suhonen	1987	not provided	Men participating in the Social Insurance Institution's Mobile Clinic Health Survey in 1973-1976	Finland	Prospective cohort	Males	5 years	40	64	Self-report	Mortality	Death certificates	22,660	140	4,532	not provided	not provided	not provided	3,564
Tavani	2004	not provided	Women in Northern Italy admitted to hospital with AMI	Italy	Case-control	Females	not provided	18	79	Self-report	Morbidity	Physician diagnosis; Medical records/disease registries	not provided	not provided	1,602	558	1,044	Communit y (patients in hospital for reasons other than AMI)	686
Tavani	2006	not provided	Cases were patients admitted to hospital with a first episode of non-fatal AMI	Italy	Case-control	Both	not provided	18	79	Self-report	Morbidity	Physician diagnosis; Medical records/disease registries	not provided	not provided	1,442	760	682	Communit y (admitted to hospital for acute conditions unrelated to diet)	573
Thun	1997	Cancer Prevention Study II	Middle-aged and elderly US adults	United States of America	Prospective cohort	Both	9 years	30	104	Self-report	Mortality	Personal inquiries; National Death Index	4,406,634	1,939	489,626	not provided	not provided	not provided	341,090
Tolstrup	2006	Danish Diet, Cancer, and Health study	Men and women born in Denmark with no previous cancers	Denmark	Prospective cohort	Both	5.7 years	50	65	Self-report	Incidence & Mortality	Danish Hospoital Discharge Register; Danish Register of Casuses of Death	304,950	2,032	53,500	not provided	not provided	not provided	52,497

Table S5. Study characteristics of included conventional observational studies

Author	Year	Study name	Population	Location	Study design	Sex	Follow-up	Age start	Age end	Exposure assessment	Endpoint	Disease ascertainment	Person-years	Events	Sample size	Cases	Controls	Control pool	Exposed
Wannameth ee	1992	British Regional Heart Study	Middle-aged men from 24 towns in England, Wales, and Scotland	England, Scotland, Wales	Prospective cohort	Males	8 years	40	59	Self-report	Mortality	Self-report; National Health Service registries	61,880	217	7,735	not provided	not provided	not provided	7,323
Wannameth ee	1999	British Regional Heart Study	Middle-aged men from 24 towns in England, Wales, and Scotland	England, Scotland, Wales	Prospective cohort	Males	16.8 years	40	59	Self-report	Incidence & Mortality	Self-report; National Health Service registries	129,948	901	7,735	not provided	not provided	not provided	7,323
Wilkins	2002	National Population Health Survey - 1994-1999	Canadian citizens over age 40 who have not been diagnosed with heart disease	Canada	Prospective cohort	Both	20 years	40	99	Self-report	Incidence & Mortality	Self-report; Statistics Canada's Canadian Mortality Database	120,280	479	6,014	not provided	not provided	not provided	not provided
Yang	2012	not provided	Men from 45 areas in China randomly selected from China's national Disease Surveillance Points	China	Prospective cohort	Males	15 years	40	79	Self-report	Mortality	Death certificates; Medical records/disease registries	3,272,835	1,437	218,189	not provided	not provided	not provided	72,866
Yi	2004	Kangwha Cohort	Residents 55+ residing in one of 10 administrative districts across Kangwha county	Republic of Korea	Prospective cohort	Males	14 years	55	99	not provided	Mortality	not provided	not provided	1,072	203	216	106	not provided	2,116
Younis	2005	Second Northwick Park Heart Study NPHS II	Subjects recruited from 9 general medical practices around England and Scotland	United Kingdom	Prospective cohort	Males	not provided	50	61	Self-report	Incidence	Hospital records	not provided	220	3,052	2,227	546	Non-drinker men within the cohort pool	2,227
Yusuf	2020	PURE	Any adult living in one of the study locations between 2005-1016	Canada, Saudi Arabia, Sweden, United Arab Emirates, Argentina, Brazil, Chile, China, Colombia, Iran (Islamic Republic of), Malaysia, Palestine, Philippines, Poland, Turkey, South Africa, Bangladesh, India, Pakistan,	Prospective cohort	Both	9.5 years	35	70	Self-report	Incidence & Mortality	Case-report forms	not provided	10,234	155,722	2,917	108,133	Never drinkers within the cohort pool	34,739

Table S5. Study characteristics of included conventional observational studies

Author	Year	Study name	Population	Location	Study design	Sex	Follow-up	Age start	Age end	Exposure assessment	Endpoint	Disease ascertainment	Person-years	Events	Sample size	Cases	Controls	Control pool	Exposed
				United Republic of Tanzania, Zimbabwe															
Zhang	2017	Donfeng-Tongji Cohort	Men aged 45-81 years who were free of coronary heart disease, stroke, or cancer from the Dongfeng-Tongji cohort	Hubei	Prospective cohort	Males	4.36 years	45	81	Self-report	Incidence & Mortality	Hospital diagnosis	15,830	959	8,469	375	500	Non-drinkers from within the cohort	3616
Zhou	2010	not provided	Patients of The First Affiliated Hospital of Nanjing Medical University and Nanjing Chest Hospital	Jiangsu	Case-control	Males	not provided	36	84	Self-report	Incidence	Biomarker	not provided	not provided	1,476	738	738	Hospital patients who did not have significant stenosis	not provided

Table S6. Study characteristics of included Mendelian randomization studies

Author	Year	Study name	Population	Location	Sex	Follow-up	Age start	Age end	Exposure assessment	Endpoint	Disease ascertainment	Person-years	Events	Sample size	Cases	Controls	Control pool	Exposed	MR method	One-/two-sample MR	Instrument	Ancestry
Au Yeung	2013	Guangzhou Biobank Cohort Study	Residents aged 50+	China	Males	not provided	50	99	Self-report	Incidence	not provided	not provided	not provided	4,867	234	not provided	not provided	2,482	2SLS	One	ALDH2 (rs671)	Asian
Biddinger	2022	UK Biobank	Participants within the UK Biobank study	United Kingdom	Both	10 years	40	69	Self-report	Incidence & Mortality	Administrative medical records	not provided	27,667	371,463	not provided	not provided	not provided	289,914	IVW, Non-linear MR	Both	9 SNPs for alcohol use disorder; 13 SNPs for AUDIT-C	European
Cho	2015	Korean Genome and Epidemiology Study (KoGES)	Subjects recruited from Ansung-Ansan cohorts	Republic of Korea	Both	not provided	39	70	Self-report	Incidence	Physician diagnosis; Self-report	not provided	122	7,152	not provided	not provided	not provided	3,387	2SLS	One	ALDH2 (rs671)	Asian
Lankester	2021	UK Biobank	Participants within the UK Biobank study	United Kingdom	Both	not provided	40	69	Self-report	Incidence & Mortality	Administrative medical records	not provided	16,102	337,484	not provided	not provided	Non-drinkers within the cohort profile	460,386	2SLS, IVW, MVMR	Both	ADH1B (rs1229984)	European
Millwood	2019	China Kadoorie Biobank	Adults from 10 diverse rural and urban areas of China	China	Males	10 years	35	74	Self-report	Incidence & Mortality	Death certificates	2,102,050	5,676	210,205	not provided	not provided	not provided	69,897	2SLS	One	ALDH2 (rs671), ADH1B (rs1229984) and a combination of 25 SNPs	Asian

Note. IVW = inverse varaiance weighted, MR = Mendelian randomization, MVMR = multivariable Mendelian randomization.

Section 5: Study quality and risk of bias assessment

Table S7. Quantified bias covariates

Author	Study design	cov_adjusted_0	cov_adjusted_1	cov_adjusted_2	cov_adjusted_3	cov_rep_prevalent_disease	cov_rep_geography	cov_exposure_study	cov_outcome_selfreport	cov_sick_quitters	cov_non_drinker	cov_older	cov_incidence	cov_mortality	cov_bmi	cov_blood_pressure	cov_cholesterol	cov_apolipoprotein	cov_fibrinogen	cov_adiponectin	cov_ihd	cov_mi	cov_design
**Conventional observational studies**																							
Albert	Prospective cohort	0	0	1	1	1	1	0	1	0	1	1	1	0	0	0	0	0	0	0	0	1	0
Arriola	Prospective cohort	0	0	1	1	1	1	1	1	1	0	1	0	0	0	0	1	0	0	0	1	0	0
Arriola	Prospective cohort	0	0	1	1	1	1	1	1	1	0	1	0	0	0	0	0	0	0	0	1	0	0
Arriola	Prospective cohort	0	0	1	1	1	1	1	1	1	0	1	0	0	0	1	0	0	0	0	1	0	0
Augustin	Case-control	0	0	0	0	0	1	1	1	0	1	1	0	1	1	1	1	0	0	0	1	0	1
Bazzano	Prospective cohort	0	0	0	0	1	0	1	1	0	1	1	1	0	1	1	0	0	0	0	0	1	0
Bazzano	Prospective cohort	0	0	0	0	1	0	1	1	0	1	0	0	1	1	1	0	0	0	0	0	1	0
Bazzano	Prospective cohort	0	0	0	0	1	0	1	1	0	1	1	0	1	1	1	0	0	0	0	0	1	0
Bell	Prospective cohort	0	0	0	0	1	0	1	1	0	0	1	0	0	1	0	0	0	0	0	0	1	0
Bergmann	Prospective cohort	0	0	0	0	1	1	1	1	0	0	1	1	0	1	0	0	0	0	0	0	1	0
Beulens	Prospective cohort	0	0	0	1	1	1	0	1	0	1	1	1	0	0	1	0	0	0	0	0	1	0
Bianchi	Case-control	0	0	0	0	1	1	1	1	1	1	1	0	1	1	1	1	0	0	0	1	0	1
Bobak	Prospective cohort	0	0	0	0	1	1	0	1	0	0	1	1	0	1	0	0	0	0	0	0	1	0
Bobak	Prospective cohort	0	0	0	0	1	1	0	1	0	1	1	1	0	1	0	0	0	0	0	0	1	0
Boffetta	Prospective cohort	0	0	0	1	1	1	1	1	0	1	1	1	0	0	0	0	0	0	0	0	1	0
Brenner	Case-control	0	0	0	0	0	1	1	1	0	1	1	0	1	1	0	1	1	1	0	0	1	1
Britton	Prospective cohort	0	0	0	0	1	1	0	1	0	0	1	0	0	1	1	1	0	0	0	1	1	0
Britton	Prospective cohort	0	0	0	0	1	1	0	1	0	1	1	0	0	1	1	1	0	0	0	1	1	0
Camargo	Prospective cohort	0	0	0	0	1	1	1	1	0	1	1	0	0	0	0	0	0	0	0	1	1	0

Table S7. Quantified bias covariates

Author	Study design	cov_adjusted_0	cov_adjusted_1	cov_adjusted_2	cov_adjusted_3	cov_rep_prevalent_disease	cov_rep_geography	cov_exposure_study	cov_outcome_selfreport	cov_sick_quitters	cov_non_drinker	cov_older	cov_incidence	cov_mortality	cov_bmi	cov_blood_pressure	cov_cholesterol	cov_apolipoprotein	cov_fibrinogen	cov_adiponectin	cov_ihd	cov_mi	cov_design
Camargo	Prospective cohort	0	0	0	0	1	1	1	1	0	1	1	0	0	0	0	0	0	0	0	1	0	0
Chang	Prospective cohort	0	0	0	0	1	0	0	0	0	1	1	0	0	1	1	1	0	0	0	0	1	0
Chiuve	Prospective cohort	0	0	0	0	1	1	0	1	0	0	1	1	0	1	0	0	0	0	0	0	1	0
Cho	Prospective cohort	0	0	0	0	1	0	1	0	0	1	1	0	1	0	0	0	0	0	0	0	1	0
Colditz	Prospective cohort	0	0	0	0	1	0	1	1	0	1	0	1	0	0	0	1	0	0	0	1	0	0
Dai	Prospective cohort	0	0	0	0	1	1	0	1	0	1	1	1	0	1	1	1	0	0	0	0	1	0
Dam	Prospective cohort	0	0	0	0	1	1	1	1	0	0	0	0	0	1	1	1	0	0	0	0	1	0
Dam	Prospective cohort	0	0	0	0	1	1	1	1	0	1	0	0	0	1	1	1	0	0	0	0	1	0
de Labry	Prospective cohort	0	0	0	0	1	1	1	1	0	0	1	1	0	0	1	1	0	0	0	0	1	0
de Labry	Prospective cohort	0	0	0	0	1	1	1	1	0	1	1	1	0	0	1	1	0	0	0	0	1	0
Doll	Prospective cohort	0	1	1	1	1	1	0	1	0	1	1	1	0	0	0	0	0	0	0	0	1	0
Dorn	Case-control	0	0	0	0	0	1	1	1	0	0	1	0	1	1	0	0	0	0	0	1	0	1
Dyer	Prospective cohort	0	1	1	1	1	1	1	1	1	1	1	1	0	0	0	0	0	0	0	1	0	0
Ebbert	Prospective cohort	0	0	0	0	1	0	1	1	0	1	0	1	0	1	1	0	0	0	0	0	1	0
Ebrahim	Prospective cohort	0	1	1	1	1	1	1	1	0	0	0	0	0	0	0	0	0	0	0	0	1	0
Degerud	Retrospective cohort	0	0	1	1	1	0	1	0	0	1	1	0	0	0	0	0	0	0	0	0	1	0
Degerud	Retrospective cohort	0	0	0	0	1	0	1	0	0	1	1	0	0	1	0	0	0	0	0	0	1	0
Fan	Case-control	0	0	0	0	0	0	1	0	0	0	1	0	1	0	0	0	0	0	0	0	1	1
Friedman	Prospective cohort	0	1	1	1	1	1	0	1	0	1	1	1	0	0	0	0	0	0	0	0	1	0
Friedman	Prospective cohort	0	1	1	1	1	1	0	1	0	1	1	0	0	0	0	0	0	0	0	0	1	0
Fuchs	Prospective cohort	0	0	0	0	1	1	1	1	0	0	1	0	0	1	1	1	0	0	0	0	1	0
Fumeron	Case-control	0	0	1	1	0	1	1	1	0	1	1	0	1	0	0	0	0	0	0	1	0	1

Table S7. Quantified bias covariates

Author	Study design	cov_adjusted_0	cov_adjusted_1	cov_adjusted_2	cov_adjusted_3	cov_rep_prevalent_disease	cov_rep_geography	cov_exposure_study	cov_outcome_selfreport	cov_sick_quitters	cov_non_drinker	cov_older	cov_incidence	cov_mortality	cov_bmi	cov_blood_pressure	cov_cholesterol	cov_apolipoprotein	cov_fibrinogen	cov_adiponectin	cov_ihd	cov_mi	cov_design
Garfinkel	Prospective cohort	0	1	1	1	1	0	1	1	1	1	1	1	0	0	0	0	0	0	0	1	0	0
Gaziano	Case-control	0	0	0	0	0	1	1	1	0	1	1	0	1	1	1	0	0	0	0	1	0	1
Genchev	Case-control	0	0	1	1	0	1	1	1	0	1	1	0	1	1	1	1	0	0	0	0	1	1
Gigleux	Prospective cohort	0	0	0	0	1	1	1	1	0	1	1	0	0	1	1	1	0	1	0	1	1	0
Goldberg	Prospective cohort	0	0	0	0	1	1	1	1	0	1	0	0	0	0	1	1	0	0	0	0	1	0
Goldberg	Prospective cohort	0	0	0	0	1	1	1	1	0	0	0	0	0	1	1	1	0	0	0	1	1	0
Gordon	Prospective cohort	0	1	1	1	1	1	1	1	0	1	1	0	0	0	0	0	0	0	0	1	0	0
Gun	Prospective cohort	0	0	0	1	1	1	0	1	0	1	1	1	0	0	0	0	0	0	0	0	1	0
Gémes	Prospective cohort	0	0	0	0	1	1	1	1	0	1	1	0	0	1	0	0	0	0	0	1	0	0
Hammar	Nested case-control	0	0	0	0	0	1	1	1	0	1	1	0	0	0	0	0	0	0	0	1	0	1
Harriss	Prospective cohort	0	0	0	0	1	1	1	1	0	0	1	1	0	0	0	0	0	0	0	0	1	0
Hart	Prospective cohort	0	0	1	1	1	1	1	1	0	1	1	1	0	0	0	0	0	0	0	0	1	0
Hart	Prospective cohort	0	0	1	1	1	1	1	1	0	1	1	0	1	0	0	0	0	0	0	0	1	0
Henderson	Prospective cohort	0	0	0	0	1	1	1	1	0	1	1	1	0	1	1	0	0	0	0	1	0	0
Hines	Case-control	0	0	0	1	1	0	1	1	1	1	1	0	1	0	0	0	0	0	0	1	0	1
Hippe	Prospective cohort	0	0	1	1	1	1	1	1	0	1	1	0	0	0	0	0	0	0	0	1	0	0
Hippe	Prospective cohort	0	0	1	1	1	0	1	1	0	1	1	0	0	0	0	0	0	0	0	1	0	0
Ikehara	Prospective cohort	0	0	0	0	1	0	1	1	0	0	1	1	0	1	1	0	0	0	0	0	1	0
Ikehara	Prospective cohort	0	0	0	0	1	0	1	1	1	0	1	1	0	1	1	0	0	0	0	1	0	0
Ilic	Case-control	0	1	1	1	0	1	1	1	0	1	1	0	1	1	1	1	0	0	0	1	0	1
Iso	Prospective cohort	0	0	0	0	1	1	1	1	0	0	1	0	0	0	1	1	0	0	0	0	1	0
Jackson	Case-control	0	0	0	0	0	1	1	1	0	0	1	0	1	0	1	0	0	0	0	1	0	1
Jakovljevic	Prospective cohort	0	1	1	1	1	0	1	1	0	1	1	1	0	1	1	0	0	0	0	1	0	0

Table S7. Quantified bias covariates

Author	Study design	cov_adjusted_0	cov_adjusted_1	cov_adjusted_2	cov_adjusted_3	cov_rep_prevalent_disease	cov_rep_geography	cov_exposure_study	cov_outcome_selfreport	cov_sick_quitters	cov_non_drinker	cov_older	cov_incidence	cov_mortality	cov_bmi	cov_blood_pressure	cov_cholesterol	cov_apolipoprotein	cov_fibrinogen	cov_adiponectin	cov_ihd	cov_mi	cov_design
Kabagambe	Case-control	0	0	0	0	0	1	1	1	0	0	1	0	1	0	1	0	0	0	0	1	0	1
Kalandidi	Case-control	0	0	1	1	0	1	1	1	0	1	1	0	1	0	0	0	0	0	0	0	1	0
Kaufman	Case-control	0	0	0	1	0	1	1	1	0	0	1	0	1	0	0	0	0	0	0	1	0	1
Kawanishi	Case-control	0	1	1	1	0	1	1	1	0	1	1	0	1	0	0	0	0	0	0	1	1	1
Keil	Prospective cohort	0	0	0	0	1	1	1	1	0	1	1	0	0	1	1	0	0	0	0	1	0	0
Key	Prospective cohort	0	0	0	1	1	0	0	1	0	0	1	1	0	0	0	0	0	0	0	1	0	0
Kitamura	Prospective cohort	0	0	1	1	1	1	0	1	0	0	1	0	0	0	0	0	0	0	0	0	1	0
Kivelä	Prospective cohort	0	0	0	0	1	1	0	1	0	1	0	1	0	1	1	1	0	0	0	0	1	0
Klatsky	Prospective cohort	0	0	0	0	1	1	1	1	0	0	1	0	1	1	0	0	0	0	0	1	0	0
Kono	Prospective cohort	0	0	0	1	1	1	1	1	0	0	1	1	0	0	0	0	0	0	0	0	1	0
Kono	Case-control	0	0	0	0	0	1	1	1	0	0	1	0	1	1	1	0	0	0	0	1	0	1
Kunutsor	Prospective cohort	0	0	1	1	1	0	1	0	0	1	1	0	0	0	0	0	0	0	0	0	1	0
Kunutsor	Prospective cohort	0	0	0	0	1	0	1	0	0	1	1	0	0	0	1	1	0	0	0	0	1	0
Kunutsor	Prospective cohort	0	0	0	0	1	0	1	0	0	1	1	0	0	1	1	1	0	0	0	0	1	0
Kurl	Prospective cohort	0	0	0	0	1	0	1	0	0	1	1	0	0	1	1	1	0	0	0	0	1	0
Lankester	Prospective cohort	0	0	0	0	1	0	1	0	1	1	1	0	0	0	0	0	0	0	0	0	1	0
Lankester	Prospective cohort	0	0	0	0	1	0	1	0	1	1	1	0	0	1	1	1	0	0	0	0	1	0
Larsson	Prospective cohort	0	0	1	1	1	1	1	1	0	0	1	0	0	0	0	0	0	0	0	1	0	0
Lazarus	Prospective cohort	0	1	1	1	1	1	0	1	0	1	1	1	0	0	0	0	0	0	0	0	1	0
Lee	Prospective cohort	0	1	1	1	1	0	1	1	1	1	0	0	1	0	0	0	0	0	0	1	0	0
Liao	Prospective cohort	0	1	1	1	1	0	1	1	0	0	1	1	0	0	0	0	0	0	0	0	1	0
Licaj	Prospective cohort	0	0	0	0	1	1	0	1	0	0	1	1	0	1	0	0	0	0	0	0	1	0
Licaj	Prospective cohort	0	0	0	0	1	1	0	1	0	1	1	1	0	1	0	0	0	0	0	0	1	0

Table S7. Quantified bias covariates

Author	Study design	cov_adjusted_0	cov_adjusted_1	cov_adjusted_2	cov_adjusted_3	cov_rep_prevalent_disease	cov_rep_geography	cov_exposure_study	cov_outcome_selfreport	cov_sick_quitters	cov_non_drinker	cov_older	cov_incidence	cov_mortality	cov_bmi	cov_blood_pressure	cov_cholesterol	cov_apolipoprotein	cov_fibrinogen	cov_adiponectin	cov_ihd	cov_mi	cov_design
Lindschou	Prospective cohort	0	0	0	0	1	1	1	1	0	0	0	0	0	1	1	1	0	0	0	1	1	0
Lindschou	Prospective cohort	0	0	0	0	1	1	1	1	0	1	0	0	0	1	1	1	0	0	0	1	1	0
Makelä	Prospective cohort	0	0	0	0	1	0	1	1	0	1	1	0	0	0	0	0	0	0	0	0	1	0
Malyutina	Prospective cohort	0	0	0	0	1	1	0	1	0	0	1	1	0	1	1	1	0	0	0	0	1	0
Malyutina	Prospective cohort	0	0	0	0	1	1	0	1	0	1	1	1	0	1	1	1	0	0	0	0	1	0
Maraldi	Prospective cohort	1	1	1	1	1	1	1	1	0	1	0	0	0	0	0	0	0	0	0	0	1	0
Marques-Vidal	Prospective cohort	0	0	0	0	1	1	1	1	0	1	0	0	0	0	1	1	0	0	0	0	1	0
Mehlig	Case-control	0	0	0	0	1	1	1	1	0	0	1	0	1	1	0	1	0	0	0	1	0	1
Mehlig	Case-control	0	0	0	0	1	1	1	1	0	1	1	0	1	1	0	1	0	0	0	1	0	1
Meisinger	Prospective cohort	0	1	1	1	1	1	1	1	0	0	1	0	0	0	0	0	0	0	0	1	1	0
Merry	Prospective cohort	0	0	0	0	1	1	1	1	0	0	1	0	0	1	1	1	0	0	0	1	0	0
Miller	Prospective cohort	0	0	0	0	1	1	1	1	0	1	1	0	0	0	1	1	0	0	0	0	1	0
Millwood	Prospective cohort	0	0	0	0	1	0	1	0	1	1	1	0	0	0	0	0	0	0	0	0	1	0
Millwood	Prospective cohort	0	0	0	0	1	1	0	1	0	0	1	0	0	0	0	0	0	0	0	1	0	0
Millwood	Prospective cohort	0	0	0	0	1	1	0	1	0	0	1	0	0	0	0	0	0	0	0	0	1	0
Miyake	Case-control	0	0	1	1	0	1	1	1	0	0	1	0	1	0	0	0	0	0	0	1	0	1
Miyake	Case-control	0	0	1	1	0	1	1	1	0	0	0	0	1	0	0	0	0	0	0	1	0	1
Mukamal	Prospective cohort	0	0	1	1	1	1	0	1	0	1	1	0	0	0	0	0	0	0	0	1	1	0
Ng	Prospective cohort	0	0	0	0	1	0	1	1	0	0	1	0	0	1	1	0	0	0	0	1	0	0
Ng	Prospective cohort	0	0	0	0	1	0	1	1	0	1	1	0	0	1	1	0	0	0	0	1	0	0
Oliveira	Case-control	0	0	0	0	1	1	1	1	0	1	1	0	1	0	0	0	0	0	0	1	0	1
Oliveira	Case-control	0	0	0	0	0	1	1	1	0	0	1	0	1	0	1	1	0	0	0	1	0	1
Oliveira	Case-control	0	0	0	0	0	1	1	1	0	1	1	0	1	0	1	1	0	0	0	1	0	1
Onat	Prospective cohort	0	0	0	1	1	0	1	1	0	1	1	0	1	0	0	0	0	0	0	0	1	0

Table S7. Quantified bias covariates

Author	Study design	cov_adjusted_0	cov_adjusted_1	cov_adjusted_2	cov_adjusted_3	cov_rep_prevalent_disease	cov_rep_geography	cov_exposure_study	cov_outcome_selfreport	cov_sick_quitters	cov_non_drinker	cov_older	cov_incidence	cov_mortality	cov_bmi	cov_blood_pressure	cov_cholesterol	cov_apolipoprotein	cov_fibrinogen	cov_adiponectin	cov_ihd	cov_mi	cov_design
Pedersen	Prospective cohort	0	0	0	0	1	1	1	1	0	1	1	1	0	1	0	1	0	0	0	0	1	0
Reddiess	Prospective cohort	0	0	1	1	1	0	1	0	0	1	1	0	1	0	0	0	0	0	0	1	0	0
Reddiess	Prospective cohort	0	0	0	0	1	0	1	0	0	1	1	0	1	1	1	0	0	0	0	1	0	0
Rehm	Prospective cohort	0	0	0	1	1	0	0	1	0	0	1	1	0	0	0	0	0	0	0	0	1	0
Renaud	Prospective cohort	0	0	0	0	1	1	1	1	0	1	1	1	0	1	1	1	0	0	0	0	1	0
Ricci	Prospective cohort	0	1	1	1	1	1	1	1	0	0	1	0	1	1	1	0	0	0	0	0	1	0
Ricci	Prospective cohort	0	1	1	1	1	1	1	1	0	1	1	0	1	1	1	0	0	0	0	0	1	0
Ricci	Prospective cohort	0	1	1	1	1	1	1	1	0	0	1	0	0	1	1	0	0	0	0	1	0	0
Ricci	Prospective cohort	0	1	1	1	1	1	1	1	0	1	1	0	0	1	1	0	0	0	0	1	0	0
Rimm	Prospective cohort	0	0	0	0	1	1	1	1	0	1	1	1	0	1	1	1	0	0	0	1	1	0
Roerecke	Prospective cohort	0	0	0	0	1	0	1	1	0	0	1	1	0	0	0	0	0	0	0	0	1	0
Roerecke	Prospective cohort	0	0	0	0	1	0	1	1	0	1	1	1	0	0	0	0	0	0	0	0	1	0
Romelsjö	Prospective cohort	0	1	1	1	1	0	1	1	0	1	1	1	0	1	0	0	0	0	0	1	0	0
Romelsjö	Prospective cohort	0	1	1	1	1	0	1	1	0	1	1	0	1	1	0	0	0	0	0	1	0	0
Romelsjö	Prospective cohort	0	1	1	1	1	0	1	1	0	1	1	0	0	1	0	0	0	0	0	1	0	0
Romelsjö	Case-control	0	0	1	1	0	0	1	1	0	0	1	0	1	0	0	0	0	0	0	1	0	1
Romelsjö	Case-control	0	0	1	1	0	0	1	1	0	1	1	0	1	0	0	0	0	0	0	1	0	1
Rostron	Prospective cohort	0	1	1	1	1	0	1	1	0	0	1	1	0	1	0	0	0	0	0	0	1	0
Ruidavets	Prospective cohort	0	0	0	0	1	1	1	1	0	0	0	0	0	0	1	0	1	0	0	1	1	0
Schooling	Prospective cohort	0	0	0	0	1	0	1	1	0	0	0	1	0	1	0	0	0	0	0	0	1	0
Schröder	Case-control	0	0	0	0	0	1	1	1	0	1	1	0	1	0	1	1	0	0	0	1	0	1
Schutte	Prospective cohort	0	0	0	0	1	0	1	0	0	1	1	0	0	0	0	0	0	0	0	0	1	0

Table S7. Quantified bias covariates

Author	Study design	cov_adjusted_0	cov_adjusted_1	cov_adjusted_2	cov_adjusted_3	cov_rep_prevalent_disease	cov_rep_geography	cov_exposure_study	cov_outcome_selfreport	cov_sick_quitters	cov_non_drinker	cov_older	cov_incidence	cov_mortality	cov_bmi	cov_blood_pressure	cov_cholesterol	cov_apolipoprotein	cov_fibrinogen	cov_adiponectin	cov_ihd	cov_mi	cov_design
Schutte	Prospective cohort	0	0	0	0	1	0	1	0	0	1	1	0	0	1	1	0	0	0	0	0	1	0
Scragg	Case-control	0	0	1	1	0	1	1	1	0	1	1	0	1	0	0	0	0	0	0	1	0	1
Sempos	Prospective cohort	0	0	1	1	1	0	0	1	0	0	1	0	0	0	0	0	0	0	0	0	1	0
Shaper	Prospective cohort	0	0	0	0	1	1	1	1	0	0	1	1	0	1	0	0	0	0	0	1	0	0
Shaper	Prospective cohort	0	0	0	0	1	1	1	1	0	1	1	1	0	1	0	0	0	0	0	1	0	0
Shiu	Prospective cohort	0	0	0	0	1	0	1	1	1	1	0	0	1	0	0	0	0	0	0	0	1	0
Simons	Prospective cohort	0	0	0	0	1	1	1	1	0	1	0	0	0	1	1	1	1	0	0	0	1	0
Skov-Ettrup	Prospective cohort	0	1	1	1	1	0	0	1	0	1	1	0	0	0	0	0	0	0	0	0	1	0
Snow	Prospective cohort	0	1	1	1	1	1	1	1	0	1	1	0	0	0	0	0	0	0	0	0	1	0
Snow	Prospective cohort	0	1	1	1	1	1	1	1	0	1	0	0	0	0	0	0	0	0	0	0	1	0
Song	Prospective cohort	0	0	0	0	1	1	1	1	0	0	1	0	0	1	0	0	0	0	0	1	1	0
Streppel	Prospective cohort	0	1	1	1	1	1	0	1	0	1	1	1	0	0	0	0	0	0	0	0	1	0
Suhonen	Prospective cohort	0	1	1	1	1	0	1	1	0	1	1	1	0	0	0	0	0	0	0	0	1	0
Suhonen	Prospective cohort	0	1	1	1	1	0	1	1	0	1	0	1	0	0	0	0	0	0	0	0	1	0
Tavani	Case-control	0	0	0	0	0	1	1	1	0	1	1	0	1	1	1	1	0	0	0	1	0	1
Tavani	Case-control	0	0	0	0	0	1	1	1	0	1	1	0	1	1	1	0	0	0	0	1	0	1
Thun	Prospective cohort	0	0	0	0	1	0	1	1	0	1	1	1	0	1	0	0	0	0	0	0	1	0
Tolstrup	Prospective cohort	0	0	0	0	1	1	1	1	0	0	0	0	0	1	0	0	0	0	0	0	1	0
Tolstrup	Prospective cohort	0	0	0	0	1	1	1	1	0	1	0	0	0	1	0	0	0	0	0	0	1	0
Wannamethee	Prospective cohort	0	0	0	0	1	1	1	1	0	0	1	1	0	0	1	0	0	0	0	1	1	0
Wannamethee	Prospective cohort	0	0	0	0	1	1	1	1	0	0	1	0	0	1	0	1	0	0	0	0	1	0
Wannamethee	Prospective cohort	0	0	0	0	1	1	1	1	0	1	1	0	0	1	0	1	0	0	0	0	1	0

Table S7. Quantified bias covariates

Author	Study design	cov_adjusted_0	cov_adjusted_1	cov_adjusted_2	cov_adjusted_3	cov_rep_prevalent_disease	cov_rep_geography	cov_exposure_study	cov_outcome_selfreport	cov_sick_quitters	cov_non_drinker	cov_older	cov_incidence	cov_mortality	cov_bmi	cov_blood_pressure	cov_cholesterol	cov_apolipoprotein	cov_fibrinogen	cov_adiponectin	cov_ihd	cov_mi	cov_design
Wilkins	Prospective cohort	0	0	0	0	1	0	1	1	0	0	1	0	0	1	1	0	0	0	0	0	1	0
Yang	Prospective cohort	0	0	1	1	1	0	1	1	0	1	1	1	0	0	0	0	0	0	0	0	1	0
Yi	Prospective cohort	0	0	0	0	1	1	1	1	0	1	1	1	0	1	1	0	0	0	0	0	1	0
Younis	Prospective cohort	0	0	0	0	1	1	1	1	0	1	0	0	1	1	0	1	0	0	0	0	1	0
Yusuf	Prospective cohort	0	0	0	0	1	0	1	1	0	0	1	0	0	0	1	1	0	0	0	1	0	0
Zhang	Prospective cohort	0	0	0	0	1	1	1	1	0	1	1	0	0	1	1	1	0	0	0	0	1	0
Zhou	Case-control	0	0	0	0	0	1	1	1	0	1	1	0	1	1	1	1	0	0	0	1	1	1
**Mendelian randomization studies**																							
Biddinger	Mendelian randomization	0	1	1	1	0	1	0	0	N/A	0	0	0	0	0	0	0	0	0	0	0	1	N/A
Cho	Mendelian randomization	0	0	0	0	1	0	1	0	N/A	1	1	0	1	0	0	0	0	0	0	0	1	N/A
Lankester	Mendelian randomization	0	1	1	1	1	0	1	0	N/A	1	1	0	0	0	0	0	0	0	0	0	1	N/A
Millwood	Mendelian randomization	0	0	1	1	1	0	1	0	N/A	1	1	0	0	0	0	0	0	0	0	0	1	N/A
Shiu	Mendelian randomization	0	0	1	1	1	0	1	1	N/A	1	0	0	1	0	0	0	0	0	0	0	1	N/A

Note. N/A = not available.

Table S8. Bias covariate definitions

Bias covariate	Definitions
cov_adjusted_0,<br>cov_adjusted_1,<br>cov_adjusted_2,<br>cov_adjusted_3	Cascading dummy variables for adjustment level of the effect sizes (i.e., for which/how many variables the effect sizes were adjusted). There are four adjustment levels, namely, (0) no adjustment, (1) only adjusted for age or sex, (2) adjusted for age and sex, and (3) adjusted for age, sex, smoking, and more than 4 other covariates.<br>If the adjustment level is 0, then cov_adjusted_0 = 1, cov_adjusted_1 = 1, cov_adjusted_2 = 1, and cov_adjusted_3 = 1;<br>if the adjustment level is 1, then cov_adjusted_0 = 0, cov_adjusted_1 = 1, cov_ adjusted _2 = 1, cov_ adjusted _3 = 1;<br>if the adjustment level is 2, then cov_ adjusted _0 = 0, cov_adjusted_1 = 0, cov_adjusted_2 = 0, cov_adjusted_3 = 1;<br>if the adjustment level is 3, then cov_adjusted_0 = 0, cov_adjusted_1= 0, cov_adjusted_2 = 0, cov_adjusted_3 = 0
cov_rep_prevalent_disease	0 for studies where participants with pre-existing disease states were excluded;<br>1 for studies of sub-groups with prevalent disease(s)
cov_rep_geography	0 for studies where the sample was from a location that was representative of the underlying geography;<br>1 for studies where the sample was in a location that was not representative of the underlying geography
cov_exposure_study	0 for studies where the exposure was measured multiple times;<br>1 for studies where the exposure was measured only once
cov_outcome_selfreport	0 for studies where the outcome was measured with self-report and at least one additional measurement technique (e.g., physician diagnosis, administrative medical records);<br>1 for studies where the outcome was measured only with self-report
cov_sick_quitters	0 if risk of reverse causation was accounted for when estimating effect sizes;<br>1 if risk of reverse causation was not accounted for
cov_non_drinker	0 if the reference group in the effect sizes were non-drinkers;<br>1 if the reference group consists of persons other than non-drinkers
cov_older	0 if effect sizes were estimated for individuals aged ≥50 years;<br>1 if effect sizes were estimated for individuals younger than 50 years.
cov_morbidity,<br>cov_mortality	cascading dummy variables for the endpoint of the effect sizes. There are two types: (1) the endpoint is morbidity or mortality only, and (2) the endpoint combines both morbidity and mortality. If the endpoint type is 1 and thus the effect sizes are for morbidity or mortality only, then either cov_morbidity = 0 and cov_mortality = 1 or cov_morbidity = 1 and cov_mortality = 0. If the endpoint type is 2, then cov_morbidity = 0 and cov_mortality = 0
cov_bmi	0 if effect sizes were not adjusted for body mass index;<br>1 if effect sizes were adjusted for body-mass index
cov_blood_pressure	0 if effect sizes were not adjusted for blood pressure;<br>1 if effect sizes were adjusted for blood pressure
cov_cholesterol	0 if effect sizes were not adjusted for cholesterol (excluding high-density lipoprotein cholesterol);<br>1 if effect sizes were adjusted for cholesterol (excluding high-density lipoprotein cholesterol)
cov_apolipoprotein	0 if effect sizes were not adjusted for apolipoprotein A1;<br>1 if effect sizes were adjusted for apolipoprotein A1
cov_fibrinogen	0 if effect sizes were not adjusted for fibrinogen;<br>1 if effect sizes were adjusted for fibrinogen
cov_adiponectin	0 if effect sizes were not adjusted for adiponectin;<br>1 if effect sizes were adjusted for adiponectin
cov_ihd	0 if the outcome mapped to the definition of ischemic heart disease;<br>1 if the outcome corresponded to subtypes of ischemic heart disease
cov_mi	0 if the outcome mapped to the definition of myocardial infarction;<br>1 if the outcome did not correspond exclusively to myocardial infarction
cov_design	0 if the study was a cohort study;<br>1 if the study was a case-control study

Section 6: Results from individual studies

Table S9. Results from input studies

Author	Study design	Reference exposure group	Alternative exposure group	Log effect size	Standard error of effect size
Albert	Prospective cohort	0 - 0.3 g/day	10 - 10 g/day	-0.36	0.25
Albert	Prospective cohort	0 - 0.3 g/day	20 - 30 g/day	0.07	0.42
Albert	Prospective cohort	0 - 0.3 g/day	0.3 - 1 g/day	0.25	0.29
Albert	Prospective cohort	0 - 0.3 g/day	1.4 - 1.4 g/day	0.17	0.28
Albert	Prospective cohort	0 - 0.3 g/day	2.9 - 5.7 g/day	-0.03	0.26
Albert	Prospective cohort	0 - 0.3 g/day	7.1 - 8.6 g/day	-0.53	0.34
Arriola	Prospective cohort	0 - 0 g/day	0 - 5 g/day	-0.43	0.23
Arriola	Prospective cohort	0 - 0 g/day	5 - 30 g/day	-0.71	0.22
Arriola	Prospective cohort	0 - 0 g/day	30 - 90 g/day	-0.78	0.22
Arriola	Prospective cohort	0 - 0 g/day	0 - 5 g/day	-0.31	0.23
Arriola	Prospective cohort	0 - 0 g/day	5 - 30 g/day	-0.45	0.28
Arriola	Prospective cohort	0 - 0 g/day	30 - 90 g/day	-1.56	1.00
Augustin	Case-control	0 - 0 g/day	0 - 12 g/day	-1.17	0.46
Augustin	Case-control	0 - 0 g/day	12 - 12 g/day	0.10	0.59
Augustin	Case-control	0 - 0 g/day	24 - 24 g/day	-0.27	0.52
Augustin	Case-control	0 - 0 g/day	36 - 54 g/day	-0.02	0.35
Bazzano	Prospective cohort	0 - 0 g/day	1.8 - 10.7 g/day	0.18	0.16
Bazzano	Prospective cohort	0 - 0 g/day	12.5 - 60.7 g/day	-0.53	0.12
Bazzano	Prospective cohort	0 - 0 g/day	62.5 - 93.8 g/day	-0.63	0.20
Bazzano	Prospective cohort	0 - 0 g/day	1.8 - 10.7 g/day	-0.08	0.17
Bazzano	Prospective cohort	0 - 0 g/day	12.5 - 60.7 g/day	-0.54	0.13
Bazzano	Prospective cohort	0 - 0 g/day	62.5 - 93.8 g/day	-0.51	0.20
Bazzano	Prospective cohort	0 - 0 g/day	1.8 - 10.7 g/day	-0.01	0.19
Bazzano	Prospective cohort	0 - 0 g/day	12.5 - 60.7 g/day	-0.20	0.13
Bazzano	Prospective cohort	0 - 0 g/day	62.5 - 93.8 g/day	-0.51	0.22
Bell	Prospective cohort	0 - 28 g/day	28 - 41.9 g/day	0.28	0.19
Bell	Prospective cohort	0 - 0 g/day	0 - 28 g/day	-0.60	0.14
Bell	Prospective cohort	0 - 28 g/day	28 - 41.9 g/day	0.44	0.20
Bell	Prospective cohort	0 - 0 g/day	0 - 28 g/day	-0.29	0.17
Bergmann	Prospective cohort	0 - 1 g/day	2 - 12 g/day	-0.42	0.11
Bergmann	Prospective cohort	0 - 1 g/day	13 - 30 g/day	-0.48	0.13
Bergmann	Prospective cohort	0 - 1 g/day	30 - 45 g/day	-0.62	0.19
Beulens	Prospective cohort	0 - 0 g/day	0.1 - 4.9 g/day	-0.17	0.18
Beulens	Prospective cohort	0 - 0 g/day	5 - 9.9 g/day	-0.29	0.21
Beulens	Prospective cohort	0 - 0 g/day	10 - 14.9 g/day	-0.78	0.24
Beulens	Prospective cohort	0 - 0 g/day	15 - 29.9 g/day	-0.45	0.21
Beulens	Prospective cohort	0 - 0 g/day	30 - 49.9 g/day	-0.67	0.25
Beulens	Prospective cohort	0 - 0 g/day	50 - 75 g/day	-0.89	0.41
Bianchi	Case-control	0 - 0 g/day	0 - 10 g/day	-0.36	0.20
Bianchi	Case-control	0 - 0 g/day	10 - 20 g/day	-0.11	0.22
Bianchi	Case-control	0 - 0 g/day	20 - 30 g/day	0.47	0.29

Table S9. Results from input studies

Author	Study design	Reference exposure group	Alternative exposure group	Log effect size	Standard error of effect size
Bianchi	Case-control	0 - 0 g/day	30 - 45 g/day	0.59	0.35
Biddinger	Mendelian randomization	0 - 0 g/day	14 - 14 g/day	0.28	0.13
Biddinger	Mendelian randomization	0 - 0 g/day	28 - 28 g/day	1.00	0.46
Biddinger	Mendelian randomization	0 - 0 g/day	42 - 42 g/day	2.15	1.00
Biddinger	Mendelian randomization	0 - 0 g/day	56 - 56 g/day	3.75	1.72
Biddinger	Mendelian randomization	0 - 0 g/day	65.2 - 65.2 g/day	5.03	2.31
Bobak	Prospective cohort	0 - 10 g/day	10 - 60 g/day	-0.08	0.11
Bobak	Prospective cohort	0 - 10 g/day	60 - 90 g/day	0.49	0.24
Bobak	Prospective cohort	0 - 5 g/day	5 - 20 g/day	-0.08	0.40
Bobak	Prospective cohort	0 - 5 g/day	20 - 30 g/day	0.33	0.72
Bobak	Prospective cohort	0 - 0 g/day	0 - 10 g/day	-0.35	0.12
Bobak	Prospective cohort	0 - 0 g/day	0 - 5 g/day	-0.34	0.17
Boffetta	Prospective cohort	0 - 0 g/day	10 - 10 g/day	-0.24	0.02
Boffetta	Prospective cohort	0 - 0 g/day	20 - 20 g/day	-0.22	0.03
Boffetta	Prospective cohort	0 - 0 g/day	30 - 30 g/day	-0.19	0.04
Boffetta	Prospective cohort	0 - 0 g/day	40 - 40 g/day	-0.30	0.05
Boffetta	Prospective cohort	0 - 0 g/day	50 - 50 g/day	-0.16	0.07
Boffetta	Prospective cohort	0 - 0 g/day	60 - 90 g/day	-0.08	0.04
Brenner	Case-control	0 - 0 g/day	0 - 17.9 g/day	-0.34	0.25
Brenner	Case-control	0 - 0 g/day	17.9 - 26.8 g/day	-0.17	0.26
Britton	Prospective cohort	1.1 - 6.9 g/day	8 - 11.4 g/day	0.13	0.16
Britton	Prospective cohort	1.1 - 6.9 g/day	12.6 - 22.9 g/day	-0.04	0.18
Britton	Prospective cohort	1.1 - 6.9 g/day	24 - 36 g/day	0.45	0.23
Britton	Prospective cohort	0 - 0 g/day	1.1 - 6.9 g/day	-0.57	0.18
Britton	Prospective cohort	0 - 0 g/day	1.1 - 6.9 g/day	-0.06	0.13
Camargo	Prospective cohort	0 - 1.4 g/day	10 - 10 g/day	-0.37	0.08
Camargo	Prospective cohort	0 - 1.4 g/day	20 - 30 g/day	-0.82	0.21
Camargo	Prospective cohort	0 - 1.4 g/day	1.4 - 1.4 g/day	0.04	0.09
Camargo	Prospective cohort	0 - 1.4 g/day	2.9 - 5.7 g/day	0.00	0.08
Camargo	Prospective cohort	0 - 1.4 g/day	7.1 - 8.6 g/day	-0.30	0.10
Camargo	Prospective cohort	0 - 1.4 g/day	10 - 10 g/day	-0.43	0.11
Camargo	Prospective cohort	0 - 1.4 g/day	20 - 30 g/day	-0.63	0.26
Camargo	Prospective cohort	0 - 1.4 g/day	1.4 - 1.4 g/day	0.08	0.12
Camargo	Prospective cohort	0 - 1.4 g/day	2.9 - 5.7 g/day	-0.04	0.11
Camargo	Prospective cohort	0 - 1.4 g/day	7.1 - 8.6 g/day	-0.20	0.14
Chang	Prospective cohort	0 - 0 g/day	0 - 10 g/day	-0.29	0.11
Chang	Prospective cohort	0 - 0 g/day	10 - 20 g/day	-0.24	0.20
Chang	Prospective cohort	0 - 0 g/day	20 - 40 g/day	-0.19	0.22
Chang	Prospective cohort	0 - 0 g/day	40 - 60 g/day	0.17	0.27

Table S9. Results from input studies

Author	Study design	Reference exposure group	Alternative exposure group	Log effect size	Standard error of effect size
Chiuve	Prospective cohort	0 - 0 g/day	0.1 - 4.9 g/day	-0.58	0.09
Chiuve	Prospective cohort	0 - 0 g/day	5 - 14.9 g/day	-0.97	0.11
Chiuve	Prospective cohort	0 - 0 g/day	15 - 29.9 g/day	-1.05	0.20
Chiuve	Prospective cohort	0 - 0 g/day	30 - 45 g/day	-0.62	0.16
Cho	Prospective cohort	0 - 0 g/day	1 - 1 g/day	0.00	0.00
Cho	Prospective cohort	0 - 0 g/day	1 - 1 g/day	0.02	0.01
Cho	Mendelian randomization	0 - 0 g/day	5.2 - 5.2 g/day	-0.01	0.25
Cho	Mendelian randomization	0 - 0 g/day	26.1 - 26.1 g/day	-0.11	0.02
Colditz	Prospective cohort	0 - 0 g/day	0.1 - 8.9 g/day	-1.20	0.50
Colditz	Prospective cohort	0 - 0 g/day	9 - 34 g/day	-0.69	0.43
Colditz	Prospective cohort	0 - 0 g/day	34 - 51 g/day	-0.22	0.72
Dai	Prospective cohort	0 - 0 g/day	1.4 - 4.9 g/day	-0.07	0.10
Dai	Prospective cohort	0 - 0 g/day	5 - 14.2 g/day	-0.06	0.09
Dai	Prospective cohort	0 - 0 g/day	14.2 - 23.9 g/day	-0.21	0.11
Dai	Prospective cohort	0 - 0 g/day	24.2 - 36.3 g/day	-0.26	0.10
Dam	Prospective cohort	1.7 - 10.3 g/day	12 - 22.3 g/day	0.00	0.07
Dam	Prospective cohort	1.7 - 10.3 g/day	24 - 34.3 g/day	-0.21	0.08
Dam	Prospective cohort	1.7 - 10.3 g/day	36 - 46.3 g/day	-0.26	0.13
Dam	Prospective cohort	1.7 - 10.3 g/day	48 - 72 g/day	-0.46	0.14
Dam	Prospective cohort	0 - 1.7 g/day	1.7 - 10.3 g/day	-0.15	0.07
de Labry	Prospective cohort	14 - 28 g/day	42 - 63 g/day	1.03	0.39
de Labry	Prospective cohort	1.2 - 42 g/day	42 - 63 g/day	0.10	0.34
de Labry	Prospective cohort	0 - 1.1 g/day	1.2 - 42 g/day	-0.34	0.64
Doll	Prospective cohort	0 - 0 g/day	1.1 - 8 g/day	-0.35	0.08
Doll	Prospective cohort	0 - 0 g/day	9.1 - 16 g/day	-0.56	0.08
Doll	Prospective cohort	0 - 0 g/day	17.1 - 32 g/day	-0.59	0.08
Doll	Prospective cohort	0 - 0 g/day	33.1 - 49.7 g/day	-0.50	0.08
Dorn	Case-control	0 - 0 g/day	0 - 12 g/day	-0.04	0.30
Dorn	Case-control	0 - 0 g/day	12 - 24 g/day	-0.40	0.26
Dorn	Case-control	0 - 0 g/day	24 - 36 g/day	-0.51	0.31
Dorn	Case-control	0 - 0 g/day	36 - 54 g/day	-0.65	0.30
Dyer	Prospective cohort	0 - 11.2 g/day	11.2 - 11.2 g/day	-0.01	0.13
Dyer	Prospective cohort	0 - 11.2 g/day	22.4 - 33.6 g/day	0.13	0.16
Dyer	Prospective cohort	0 - 11.2 g/day	44.8 - 56 g/day	0.43	0.28
Dyer	Prospective cohort	0 - 11.2 g/day	67.2 - 100.8 g/day	0.39	0.25
Ebbert	Prospective cohort	0 - 0 g/day	0 - 14 g/day	-0.26	0.12
Ebbert	Prospective cohort	0 - 0 g/day	14 - 21 g/day	-0.69	0.38
Ebrahim	Prospective cohort	0 - 0 g/day	0.7 - 8.6 g/day	-0.17	0.23
Ebrahim	Prospective cohort	0 - 0 g/day	10 - 30 g/day	-0.39	0.30
Ebrahim	Prospective cohort	0 - 0 g/day	30 - 45 g/day	-0.22	1.00
Degerud	Retrospective cohort	0 - 2 g/day	2 - 12 g/day	0.09	0.11
Degerud	Retrospective cohort	0 - 2 g/day	12 - 24 g/day	-0.20	0.18

Table S9. Results from input studies

Author	Study design	Reference exposure group	Alternative exposure group	Log effect size	Standard error of effect size
Degerud	Retrospective cohort	0 - 2 g/day	24 - 60 g/day	-0.42	0.36
Degerud	Retrospective cohort	0 - 2 g/day	2 - 60 g/day	-0.03	0.09
Degerud	Retrospective cohort	0 - 2 g/day	2 - 12 g/day	-0.02	0.11
Degerud	Retrospective cohort	0 - 2 g/day	12 - 24 g/day	-0.27	0.18
Degerud	Retrospective cohort	0 - 2 g/day	24 - 60 g/day	-0.49	0.38
Degerud	Retrospective cohort	0 - 2 g/day	2 - 60 g/day	-0.12	0.09
Degerud	Retrospective cohort	0 - 2 g/day	2 - 60 g/day	-0.09	0.09
Fan	Case-control	0 - 3.1 g/day	3.1 - 17.9 g/day	-0.15	0.14
Fan	Case-control	0 - 3.1 g/day	17.9 - 59.7 g/day	-0.27	0.15
Fan	Case-control	0 - 3.1 g/day	59.8 - 89.7 g/day	0.43	0.14
Fan	Case-control	0 - 0.4 g/day	0.4 - 4 g/day	-0.15	0.12
Fan	Case-control	0 - 0.4 g/day	4 - 20.6 g/day	-0.15	0.12
Fan	Case-control	0 - 0.4 g/day	20.6 - 30.9 g/day	-0.08	0.12
Fan	Case-control	0 - 1.2 g/day	1.2 - 6.7 g/day	-0.29	0.15
Fan	Case-control	0 - 1.2 g/day	6.7 - 23.4 g/day	-0.67	0.16
Fan	Case-control	0 - 1.2 g/day	23.4 - 35.1 g/day	-0.53	0.15
Fan	Case-control	0 - 0.4 g/day	0.4 - 2 g/day	-0.04	0.12
Fan	Case-control	0 - 0.4 g/day	2 - 9.1 g/day	-0.31	0.13
Fan	Case-control	0 - 0.4 g/day	9.1 - 13.7 g/day	-0.43	0.14
Fan	Case-control	0 - 0 g/day	0 - 3.1 g/day	0.33	0.19
Fan	Case-control	0 - 0 g/day	3.1 - 17.9 g/day	0.17	0.19
Fan	Case-control	0 - 0 g/day	17.9 - 59.7 g/day	0.04	0.20
Fan	Case-control	0 - 0 g/day	59.8 - 89.7 g/day	0.74	0.19
Fan	Case-control	0 - 0 g/day	0 - 0.4 g/day	0.25	0.12
Fan	Case-control	0 - 0 g/day	0.4 - 4 g/day	0.09	0.13
Fan	Case-control	0 - 0 g/day	4 - 20.6 g/day	0.10	0.13
Fan	Case-control	0 - 0 g/day	20.6 - 30.9 g/day	0.18	0.13
Fan	Case-control	0 - 0 g/day	0 - 1.2 g/day	0.53	0.19
Fan	Case-control	0 - 0 g/day	1.2 - 6.7 g/day	0.24	0.20
Fan	Case-control	0 - 0 g/day	6.7 - 23.4 g/day	-0.14	0.21
Fan	Case-control	0 - 0 g/day	23.4 - 35.1 g/day	0.01	0.21
Fan	Case-control	0 - 0 g/day	0 - 0.4 g/day	0.21	0.13
Fan	Case-control	0 - 0 g/day	0.4 - 2 g/day	0.17	0.14
Fan	Case-control	0 - 0 g/day	2 - 9.1 g/day	-0.11	0.15
Fan	Case-control	0 - 0 g/day	9.1 - 13.7 g/day	-0.21	0.15
Friedman	Prospective cohort	0 - 0 g/day	3.3 - 3.3 g/day	-0.13	0.24
Friedman	Prospective cohort	0 - 0 g/day	6.7 - 10 g/day	-0.14	0.31
Friedman	Prospective cohort	0 - 0 g/day	13.3 - 23.3 g/day	-0.69	0.38
Friedman	Prospective cohort	0 - 0 g/day	26.7 - 63.3 g/day	-1.20	0.28
Friedman	Prospective cohort	0 - 0 g/day	66.7 - 100 g/day	-0.69	0.41
Friedman	Prospective cohort	0 - 0 g/day	3.3 - 3.3 g/day	-0.02	0.25
Friedman	Prospective cohort	0 - 0 g/day	6.7 - 10 g/day	-1.20	0.53
Friedman	Prospective cohort	0 - 0 g/day	13.3 - 30 g/day	0.00	0.46
Friedman	Prospective cohort	0 - 0 g/day	33.3 - 50 g/day	0.00	0.76

Table S9. Results from input studies

Author	Study design	Reference exposure group	Alternative exposure group	Log effect size	Standard error of effect size
Fuchs	Prospective cohort	0 - 0 g/day	0.1 - 10 g/day	0.05	0.21
Fuchs	Prospective cohort	0 - 0 g/day	10 - 20 g/day	-0.21	0.25
Fuchs	Prospective cohort	0 - 0 g/day	20 - 30 g/day	-0.21	0.30
Fuchs	Prospective cohort	0 - 0 g/day	30 - 45 g/day	-0.37	0.28
Fuchs	Prospective cohort	0 - 0 g/day	0.1 - 10 g/day	-0.45	0.29
Fuchs	Prospective cohort	0 - 0 g/day	10 - 15 g/day	-0.60	0.37
Fuchs	Prospective cohort	0 - 0 g/day	0.1 - 0.2 g/day	-0.71	0.45
Fuchs	Prospective cohort	0 - 0 g/day	0 - 0.1 g/day	-0.71	0.27
Fuchs	Prospective cohort	0 - 0 g/day	0 - 1.9 g/day	-0.24	0.23
Fuchs	Prospective cohort	0 - 0 g/day	0 - 1.9 g/day	-0.71	0.27
Fuchs	Prospective cohort	0 - 0 g/day	0 - 1.9 g/day	-0.99	1.02
Fumeron	Case-control	0 - 0 g/day	0 - 25 g/day	0.10	0.17
Fumeron	Case-control	0 - 0 g/day	25 - 50 g/day	-0.07	0.18
Fumeron	Case-control	0 - 0 g/day	50 - 75 g/day	-0.33	0.21
Fumeron	Case-control	0 - 0 g/day	75 - 112.5 g/day	-0.33	0.21
Garfinkel	Prospective cohort	0 - 0 g/day	5 - 15 g/day	-0.21	0.03
Garfinkel	Prospective cohort	0 - 0 g/day	15 - 25 g/day	-0.27	0.04
Garfinkel	Prospective cohort	0 - 0 g/day	25 - 35 g/day	-0.56	0.08
Garfinkel	Prospective cohort	0 - 0 g/day	35 - 45 g/day	-0.31	0.09
Garfinkel	Prospective cohort	0 - 0 g/day	45 - 55 g/day	-0.65	0.19
Garfinkel	Prospective cohort	0 - 0 g/day	55 - 90 g/day	-0.54	0.12
Gaziano	Case-control	0 - 0.3 g/day	13.2 - 39.6 g/day	-0.67	0.24
Gaziano	Case-control	0 - 0.3 g/day	39.6 - 59.4 g/day	-0.80	0.29
Gaziano	Case-control	0 - 0.3 g/day	0.4 - 12.8 g/day	0.02	0.22
Genchev	Case-control	0 - 0 g/day	0 - 18 g/day	-0.39	0.39
Genchev	Case-control	0 - 0 g/day	18 - 36 g/day	-0.94	0.43
Genchev	Case-control	0 - 0 g/day	36 - 54 g/day	-0.49	0.52
Genchev	Case-control	0 - 0 g/day	54 - 81 g/day	-0.15	0.75
Gigleux	Prospective cohort	0 - 1.3 g/day	1.3 - 5.4 g/day	-0.13	0.20
Gigleux	Prospective cohort	0 - 1.3 g/day	5.5 - 15.1 g/day	-0.21	0.20
Gigleux	Prospective cohort	0 - 1.3 g/day	15.2 - 22.8 g/day	-0.65	0.23
Goldberg	Prospective cohort	0 - 0 g/day	0.8 - 11 g/day	-0.21	0.16
Goldberg	Prospective cohort	0 - 0 g/day	11.8 - 30.8 g/day	-0.39	0.28
Goldberg	Prospective cohort	0 - 0 g/day	31.6 - 47.4 g/day	-0.80	0.30
Goldberg	Prospective cohort	0 - 0 g/day	0.8 - 11 g/day	-0.51	0.26
Goldberg	Prospective cohort	0 - 0 g/day	11.8 - 30.8 g/day	-1.27	0.71
Goldberg	Prospective cohort	0 - 0 g/day	31.6 - 47.4 g/day	-0.33	0.43
Goldberg	Prospective cohort	0 - 0 g/day	0 - 2.9 g/day	-0.03	0.21
Goldberg	Prospective cohort	0 - 0 g/day	3 - 18.8 g/day	-0.58	0.27
Goldberg	Prospective cohort	0 - 0 g/day	18.9 - 28.3 g/day	-0.60	0.27
Gordon	Prospective cohort	0 - 0 g/day	0.8 - 7 g/day	-0.46	0.14
Gordon	Prospective cohort	0 - 0 g/day	7.8 - 14.8 g/day	-0.37	0.15
Gordon	Prospective cohort	0 - 0 g/day	15.6 - 22.6 g/day	-0.40	0.19
Gordon	Prospective cohort	0 - 0 g/day	23.3 - 45.9 g/day	-0.48	0.18

Table S9. Results from input studies

Author	Study design	Reference exposure group	Alternative exposure group	Log effect size	Standard error of effect size
Gordon	Prospective cohort	0 - 0 g/day	46.7 - 69.2 g/day	0.00	0.23
Gordon	Prospective cohort	0 - 0 g/day	70 - 105 g/day	0.34	0.28
Gun	Prospective cohort	0 - 0 g/day	1.4 - 10 g/day	-0.51	0.17
Gun	Prospective cohort	0 - 0 g/day	11.4 - 30 g/day	-0.45	0.17
Gun	Prospective cohort	0 - 0 g/day	31.4 - 50 g/day	-0.67	0.21
Gun	Prospective cohort	0 - 0 g/day	51.4 - 70 g/day	-0.12	0.22
Gun	Prospective cohort	0 - 0 g/day	71.4 - 107.1 g/day	-0.26	0.22
Gémes	Prospective cohort	0 - 0.9 g/day	0.9 - 4.3 g/day	-0.13	0.06
Gémes	Prospective cohort	0 - 0.9 g/day	4.3 - 8.6 g/day	-0.29	0.07
Gémes	Prospective cohort	0 - 0.9 g/day	8.6 - 12.9 g/day	-0.17	0.11
Gémes	Prospective cohort	0 - 0.9 g/day	12.9 - 19.3 g/day	-0.36	0.16
Hammar	Nested case-control	0 - 0 g/day	0 - 20 g/day	-0.51	0.21
Hammar	Nested case-control	0 - 0 g/day	20 - 30 g/day	-0.69	0.41
Hammar	Nested case-control	0 - 0 g/day	30 - 45 g/day	-0.36	0.48
Harriss	Prospective cohort	0 - 0 g/day	0 - 1.4 g/day	0.06	0.36
Harriss	Prospective cohort	0 - 0 g/day	1.4 - 20 g/day	-0.48	0.34
Harriss	Prospective cohort	0 - 0 g/day	20 - 40 g/day	0.10	0.32
Harriss	Prospective cohort	0 - 0 g/day	40 - 60 g/day	-1.66	0.71
Hart	Prospective cohort	0 - 0 g/day	1.1 - 8 g/day	0.01	0.08
Hart	Prospective cohort	0 - 0 g/day	9.1 - 16 g/day	-0.14	0.09
Hart	Prospective cohort	0 - 0 g/day	17.1 - 24 g/day	0.25	0.10
Hart	Prospective cohort	0 - 0 g/day	25.1 - 38.9 g/day	0.09	0.10
Hart	Prospective cohort	0 - 0 g/day	40 - 60 g/day	0.24	0.11
Hart	Prospective cohort	0 - 0 g/day	1.1 - 8 g/day	-0.12	0.08
Hart	Prospective cohort	0 - 0 g/day	9.1 - 16 g/day	-0.16	0.08
Hart	Prospective cohort	0 - 0 g/day	17.1 - 24 g/day	0.02	0.09
Hart	Prospective cohort	0 - 0 g/day	25.1 - 38.9 g/day	-0.20	0.10
Hart	Prospective cohort	0 - 0 g/day	40 - 60 g/day	-0.04	0.11
Henderson	Prospective cohort	0 - 0 g/day	0 - 12 g/day	-0.19	0.14
Henderson	Prospective cohort	0 - 0 g/day	12 - 18 g/day	-0.40	0.22
Hines	Case-control	0 - 1.4 g/day	1.4 - 10 g/day	0.01	0.15
Hines	Case-control	0 - 1.4 g/day	10 - 15 g/day	-0.45	0.17
Hippe	Prospective cohort	0 - 1.6 g/day	1.1 - 6.9 g/day	-0.30	0.10
Hippe	Prospective cohort	0 - 1.6 g/day	8 - 14.9 g/day	-0.29	0.14
Hippe	Prospective cohort	0 - 1.6 g/day	16 - 30.9 g/day	-0.29	0.18
Hippe	Prospective cohort	0 - 1.6 g/day	1.1 - 6.9 g/day	-0.30	0.10
Hippe	Prospective cohort	0 - 1.6 g/day	8 - 14.9 g/day	-0.43	0.09
Hippe	Prospective cohort	0 - 1.6 g/day	16 - 30.9 g/day	-0.48	0.09
Hippe	Prospective cohort	0 - 1.6 g/day	32 - 48 g/day	-0.54	0.11
Hippe	Prospective cohort	0 - 1.6 g/day	1.6 - 9.4 g/day	-0.30	0.10
Hippe	Prospective cohort	0 - 1.6 g/day	11 - 20.4 g/day	-0.43	0.09
Hippe	Prospective cohort	0 - 1.6 g/day	22 - 42.4 g/day	-0.48	0.09
Hippe	Prospective cohort	0 - 1.6 g/day	44 - 66 g/day	-0.54	0.11
Hippe	Prospective cohort	0 - 1.6 g/day	1.6 - 9.4 g/day	-0.30	0.10

Table S9. Results from input studies

Author	Study design	Reference exposure group	Alternative exposure group	Log effect size	Standard error of effect size
Hippe	Prospective cohort	0 - 1.6 g/day	11 - 20.4 g/day	-0.29	0.14
Hippe	Prospective cohort	0 - 1.6 g/day	22 - 42.4 g/day	-0.29	0.18
Ikehara	Prospective cohort	0 - 0 g/day	0.1 - 22.9 g/day	-0.04	0.15
Ikehara	Prospective cohort	0 - 0 g/day	23 - 45.9 g/day	-0.20	0.14
Ikehara	Prospective cohort	0 - 0 g/day	46 - 68.9 g/day	-0.27	0.16
Ikehara	Prospective cohort	0 - 0 g/day	69 - 103.5 g/day	-0.05	0.20
Ikehara	Prospective cohort	0 - 0 g/day	0.1 - 22.9 g/day	-0.19	0.23
Ikehara	Prospective cohort	0 - 0 g/day	23 - 45.9 g/day	0.37	0.39
Ikehara	Prospective cohort	0 - 0 g/day	46 - 69 g/day	1.41	0.47
Ikehara	Prospective cohort	0 - 0 g/day	0.1 - 21.3 g/day	-1.14	0.23
Ikehara	Prospective cohort	0 - 0 g/day	21.4 - 42.7 g/day	-1.47	0.25
Ikehara	Prospective cohort	0 - 0 g/day	42.9 - 64.1 g/day	-0.92	0.25
Ikehara	Prospective cohort	0 - 0 g/day	64.3 - 96.4 g/day	-1.43	0.35
Ilic	Case-control	0 - 0 g/day	33.6 - 50.4 g/day	0.83	0.40
Ilic	Case-control	0 - 0 g/day	0 - 65 g/day	-0.11	0.41
Ilic	Case-control	0 - 0 g/day	0 - 52 g/day	-0.11	0.46
Iso	Prospective cohort	0 - 0.9 g/day	1 - 20 g/day	-0.11	0.60
Iso	Prospective cohort	0 - 0.9 g/day	21 - 41 g/day	-0.36	0.60
Iso	Prospective cohort	0 - 0.9 g/day	42 - 69 g/day	-0.11	0.47
Iso	Prospective cohort	0 - 0.9 g/day	70 - 105 g/day	-0.22	0.67
Jackson	Case-control	0 - 0 g/day	0 - 4.6 g/day	-0.56	0.29
Jackson	Case-control	0 - 0 g/day	5.7 - 16 g/day	-0.56	0.29
Jackson	Case-control	0 - 0 g/day	17.1 - 40 g/day	-0.67	0.29
Jackson	Case-control	0 - 0 g/day	41.1 - 64 g/day	-0.54	0.40
Jackson	Case-control	0 - 0 g/day	64 - 96 g/day	0.21	0.41
Jackson	Case-control	0 - 0 g/day	0 - 4.6 g/day	-0.67	0.36
Jackson	Case-control	0 - 0 g/day	5.7 - 16 g/day	-1.77	0.54
Jackson	Case-control	0 - 0 g/day	17.1 - 40 g/day	-1.90	0.66
Jackson	Case-control	0 - 0 g/day	41.1 - 64 g/day	-0.87	1.20
Jakovljevic	Prospective cohort	0 - 11 g/day	11 - 22 g/day	-0.66	0.54
Jakovljevic	Prospective cohort	0 - 11 g/day	22 - 33 g/day	0.90	0.43
Kabagambe	Case-control	0 - 0 g/day	0 - 4.9 g/day	-0.26	0.12
Kalandidi	Case-control	0 - 10 g/day	10 - 30 g/day	-0.17	0.23
Kalandidi	Case-control	0 - 10 g/day	30 - 45 g/day	-0.24	0.26
Kaufman	Case-control	0 - 0 g/day	0 - 16.7 g/day	0.10	0.24
Kaufman	Case-control	0 - 0 g/day	16.7 - 30 g/day	0.10	0.25
Kaufman	Case-control	0 - 0 g/day	33.3 - 63.3 g/day	0.41	0.26
Kaufman	Case-control	0 - 0 g/day	66.7 - 100 g/day	0.10	0.28
Kawanishi	Case-control	0 - 25 g/day	25 - 37.5 g/day	-0.76	0.36
Keil	Prospective cohort	0 - 0 g/day	0.1 - 2 g/day	-0.62	0.41
Keil	Prospective cohort	0 - 0 g/day	20 - 39.9 g/day	-0.73	0.42
Keil	Prospective cohort	0 - 0 g/day	40 - 79.9 g/day	-0.46	0.37
Keil	Prospective cohort	0 - 0 g/day	80 - 120 g/day	-0.73	0.50
Key	Prospective cohort	1 - 7 g/day	8 - 15 g/day	-0.31	0.21

Table S9. Results from input studies

Author	Study design	Reference exposure group	Alternative exposure group	Log effect size	Standard error of effect size
Key	Prospective cohort	1 - 7 g/day	16 - 24 g/day	-0.14	0.21
Kitamura	Prospective cohort	0 - 0 g/day	1 - 22 g/day	-0.37	0.32
Kitamura	Prospective cohort	0 - 0 g/day	23 - 45 g/day	-0.60	0.33
Kitamura	Prospective cohort	0 - 0 g/day	46 - 68 g/day	-0.82	0.39
Kitamura	Prospective cohort	0 - 0 g/day	69 - 103.5 g/day	-0.33	0.48
Kivelä	Prospective cohort	0 - 0 g/day	0 - 9.1 g/day	0.00	0.27
Kivelä	Prospective cohort	0 - 0 g/day	9.1 - 13.6 g/day	0.18	0.29
Klatsky	Prospective cohort	0 - 0 g/day	0 - 0.2 g/day	-0.11	0.06
Klatsky	Prospective cohort	0 - 0 g/day	0 - 5 g/day	-0.36	0.07
Klatsky	Prospective cohort	0 - 0 g/day	10 - 20 g/day	-0.51	0.09
Klatsky	Prospective cohort	0 - 0 g/day	30 - 50 g/day	-0.51	0.12
Klatsky	Prospective cohort	0 - 0 g/day	60 - 90 g/day	-0.69	0.25
Kono	Prospective cohort	0 - 0 g/day	0 - 42.6 g/day	-0.36	0.20
Kono	Prospective cohort	0 - 0 g/day	42.6 - 63.9 g/day	-0.36	0.26
Kono	Case-control	0 - 0 g/day	0 - 23.7 g/day	0.10	0.40
Kono	Case-control	0 - 0 g/day	23.7 - 46.6 g/day	-1.17	0.52
Kono	Case-control	0 - 0 g/day	47.4 - 71 g/day	-2.04	0.50
Kunutsor	Prospective cohort	0 - 0 g/day	9.3 - 28 g/day	-0.30	0.18
Kunutsor	Prospective cohort	0 - 0 g/day	28 - 42 g/day	-0.43	0.33
Kunutsor	Prospective cohort	0 - 0 g/day	9.3 - 28 g/day	-0.17	0.19
Kunutsor	Prospective cohort	0 - 0 g/day	28 - 42 g/day	-0.39	0.33
Kunutsor	Prospective cohort	0 - 0 g/day	9.3 - 28 g/day	-0.14	0.19
Kunutsor	Prospective cohort	0 - 0 g/day	28 - 42 g/day	-0.33	0.34
Kunutsor	Prospective cohort	0 - 0 g/day	9.3 - 28 g/day	-0.14	0.19
Kunutsor	Prospective cohort	0 - 0 g/day	28 - 42 g/day	-0.36	0.34
Kunutsor	Prospective cohort	0 - 0 g/day	0.3 - 1.1 g/day	-0.62	0.22
Kunutsor	Prospective cohort	0 - 0 g/day	0.3 - 1.1 g/day	-0.48	0.22
Kunutsor	Prospective cohort	0 - 0 g/day	0.3 - 1.1 g/day	-0.48	0.22
Kunutsor	Prospective cohort	0 - 0 g/day	0.3 - 1.1 g/day	-0.48	0.22
Kunutsor	Prospective cohort	0 - 0 g/day	2.3 - 8 g/day	-0.22	0.17
Kunutsor	Prospective cohort	0 - 0 g/day	2.3 - 8 g/day	-0.12	0.18
Kunutsor	Prospective cohort	0 - 0 g/day	2.3 - 8 g/day	-0.09	0.18
Kunutsor	Prospective cohort	0 - 0 g/day	2.3 - 8 g/day	-0.11	0.18
Kurl	Prospective cohort	0 - 1.4 g/day	1.4 - 2.1 g/day	0.01	0.01
Lankester	Prospective cohort	0 - 0 g/day	0 - 10 g/day	-0.12	0.03
Lankester	Prospective cohort	0 - 0 g/day	10 - 20 g/day	-0.20	0.03
Lankester	Prospective cohort	0 - 0 g/day	20 - 30 g/day	-0.21	0.04
Lankester	Prospective cohort	0 - 0 g/day	30 - 45 g/day	-0.16	0.04
Lankester	Mendelian randomization	0 - 0 g/day	19.4 - 19.4 g/day	0.11	0.11
Lankester	Mendelian randomization	0 - 0 g/day	19.4 - 19.4 g/day	0.29	0.08
Lankester	Mendelian randomization	0 - 0 g/day	19.4 - 19.4 g/day	0.59	0.14

Table S9. Results from input studies

Author	Study design	Reference exposure group	Alternative exposure group	Log effect size	Standard error of effect size
Larsson	Prospective cohort	0 - 1.7 g/day	1.7 - 10.3 g/day	-0.09	0.06
Larsson	Prospective cohort	0 - 1.7 g/day	12 - 24 g/day	-0.19	0.06
Larsson	Prospective cohort	0 - 1.7 g/day	25.7 - 36 g/day	-0.20	0.08
Larsson	Prospective cohort	0 - 1.7 g/day	37.7 - 48 g/day	-0.24	0.12
Larsson	Prospective cohort	0 - 1.7 g/day	48 - 72 g/day	-0.17	0.11
Larsson	Prospective cohort	0 - 1.7 g/day	1.7 - 10.3 g/day	-0.19	0.06
Larsson	Prospective cohort	0 - 1.7 g/day	12 - 24 g/day	-0.53	0.12
Larsson	Prospective cohort	0 - 1.7 g/day	25.7 - 36 g/day	-1.11	0.38
Larsson	Prospective cohort	0 - 1.7 g/day	36 - 54 g/day	0.00	0.10
Larsson	Prospective cohort	0 - 0 g/day	0 - 1.7 g/day	0.20	0.09
Larsson	Prospective cohort	0 - 0 g/day	0 - 1.7 g/day	0.03	0.07
Lazarus	Prospective cohort	0 - 0 g/day	0.3 - 10 g/day	0.46	0.17
Lazarus	Prospective cohort	0 - 0 g/day	10.3 - 15.5 g/day	0.87	0.23
Lazarus	Prospective cohort	0 - 0 g/day	0.3 - 10 g/day	0.46	0.20
Lazarus	Prospective cohort	0 - 0 g/day	10.3 - 15.5 g/day	0.40	0.24
Lee	Prospective cohort	0 - 0 g/day	1 - 14 g/day	-0.51	0.05
Lee	Prospective cohort	0 - 0 g/day	15 - 22.5 g/day	-0.76	0.12
Liao	Prospective cohort	0 - 0 g/day	0 - 10 g/day	-0.63	0.15
Liao	Prospective cohort	0 - 0 g/day	10 - 20 g/day	-0.80	0.18
Liao	Prospective cohort	0 - 0 g/day	20 - 30 g/day	-0.82	0.20
Liao	Prospective cohort	0 - 0 g/day	20 - 40 g/day	-0.76	0.25
Liao	Prospective cohort	0 - 0 g/day	40 - 60 g/day	-0.63	0.21
Liao	Prospective cohort	0 - 0 g/day	0 - 10 g/day	-1.31	0.10
Liao	Prospective cohort	0 - 0 g/day	10 - 20 g/day	-1.11	0.17
Liao	Prospective cohort	0 - 0 g/day	20 - 30 g/day	-0.69	0.25
Liao	Prospective cohort	0 - 0 g/day	20 - 40 g/day	-0.51	0.41
Liao	Prospective cohort	0 - 0 g/day	40 - 60 g/day	-1.61	0.47
Licaj	Prospective cohort	0.1 - 1.4 g/day	1.5 - 4.9 g/day	-0.30	0.30
Licaj	Prospective cohort	0.1 - 1.4 g/day	5 - 9.9 g/day	-0.46	0.37
Licaj	Prospective cohort	0.1 - 1.4 g/day	10 - 14.9 g/day	-1.20	0.61
Licaj	Prospective cohort	0.1 - 1.4 g/day	15 - 22.5 g/day	-1.56	0.76
Licaj	Prospective cohort	0 - 0 g/day	0.1 - 1.4 g/day	0.02	0.34
Lindschou	Prospective cohort	1.7 - 10.3 g/day	12 - 22.3 g/day	-0.17	0.13
Lindschou	Prospective cohort	1.7 - 10.3 g/day	24 - 34.3 g/day	-0.25	0.17
Lindschou	Prospective cohort	1.7 - 10.3 g/day	36 - 46.3 g/day	-0.54	0.18
Lindschou	Prospective cohort	1.7 - 10.3 g/day	48 - 72 g/day	-0.51	0.17
Lindschou	Prospective cohort	0 - 1.7 g/day	1.7 - 10.3 g/day	-0.29	0.17
Makelä	Prospective cohort	0 - 0 g/day	0 - 1.3 g/day	-0.04	0.13
Makelä	Prospective cohort	0 - 0 g/day	1.3 - 4.5 g/day	-0.29	0.14
Makelä	Prospective cohort	0 - 0 g/day	4.6 - 11.7 g/day	-0.13	0.14
Makelä	Prospective cohort	0 - 0 g/day	11.7 - 17.5 g/day	-0.46	0.16
Makelä	Prospective cohort	0 - 0 g/day	0 - 0.2 g/day	0.07	0.14
Makelä	Prospective cohort	0 - 0 g/day	0.2 - 0.6 g/day	-0.31	0.22
Makelä	Prospective cohort	0 - 0 g/day	0.7 - 2.6 g/day	-0.29	0.22

Table S9. Results from input studies

Author	Study design	Reference exposure group	Alternative exposure group	Log effect size	Standard error of effect size
Makelä	Prospective cohort	0 - 0 g/day	2.6 - 3.9 g/day	-0.24	0.25
Malyutina	Prospective cohort	0 - 0 g/day	0 - 5.7 g/day	-0.54	0.35
Malyutina	Prospective cohort	0 - 0 g/day	5.7 - 11.3 g/day	-0.27	0.23
Malyutina	Prospective cohort	0 - 0 g/day	11.4 - 17 g/day	-0.15	0.23
Malyutina	Prospective cohort	0 - 0 g/day	17.1 - 22.7 g/day	0.12	0.27
Malyutina	Prospective cohort	0 - 0 g/day	22.9 - 34.3 g/day	0.00	0.20
Malyutina	Prospective cohort	0 - 11.4 g/day	11.4 - 17.1 g/day	0.12	0.16
Malyutina	Prospective cohort	0 - 11.4 g/day	17.1 - 25.7 g/day	0.16	0.19
Malyutina	Prospective cohort	0 - 11.4 g/day	22.9 - 34.3 g/day	0.24	0.23
Malyutina	Prospective cohort	0 - 0 g/day	0 - 0 g/day	-0.49	0.17
Malyutina	Prospective cohort	0 - 0 g/day	0 - 11.4 g/day	-0.59	0.18
Maraldi	Prospective cohort	0 - 2 g/day	2 - 14 g/day	-0.05	0.16
Maraldi	Prospective cohort	0 - 2 g/day	14 - 21 g/day	0.34	0.22
Marques-Vidal	Prospective cohort	0 - 0 g/day	0 - 14.4 g/day	-0.30	0.25
Marques-Vidal	Prospective cohort	0 - 0 g/day	14.4 - 29.9 g/day	-0.43	0.26
Marques-Vidal	Prospective cohort	0 - 0 g/day	29.9 - 49.7 g/day	-0.73	0.27
Marques-Vidal	Prospective cohort	0 - 0 g/day	49.7 - 74.6 g/day	-0.99	0.27
Mehlig	Case-control	0 - 4.9 g/day	4.9 - 9.8 g/day	-0.43	0.13
Mehlig	Case-control	0 - 4.9 g/day	9.8 - 14.7 g/day	-0.11	0.14
Mehlig	Case-control	0 - 0 g/day	0 - 4.9 g/day	-0.10	0.18
Meisinger	Prospective cohort	0 - 0 g/day	0.1 - 39.9 g/day	-0.48	0.28
Meisinger	Prospective cohort	0 - 0 g/day	40 - 60 g/day	-0.16	0.25
Merry	Prospective cohort	0 - 0 g/day	0 - 20 g/day	-0.37	0.16
Merry	Prospective cohort	0 - 0 g/day	20 - 40 g/day	-0.49	0.19
Merry	Prospective cohort	0 - 0 g/day	40 - 60 g/day	-0.62	0.24
Miller	Prospective cohort	0 - 0 g/day	1.4 - 5.7 g/day	-0.19	0.37
Miller	Prospective cohort	0 - 0 g/day	7.1 - 20 g/day	-0.78	0.41
Miller	Prospective cohort	0 - 0 g/day	21.4 - 84.3 g/day	-1.17	0.53
Millwood	Prospective cohort	0 - 0 g/day	0 - 20 g/day	0.00	0.03
Millwood	Prospective cohort	0 - 0 g/day	20 - 39.9 g/day	0.03	0.03
Millwood	Prospective cohort	0 - 0 g/day	40 - 59.9 g/day	0.10	0.04
Millwood	Prospective cohort	0 - 0 g/day	60 - 90 g/day	0.12	0.04
Millwood	Prospective cohort	0 - 20 g/day	20 - 39.9 g/day	0.10	0.07
Millwood	Prospective cohort	0 - 20 g/day	40 - 59.9 g/day	0.17	0.09
Millwood	Prospective cohort	0 - 20 g/day	60 - 90 g/day	0.13	0.09
Millwood	Prospective cohort	0 - 10 g/day	10 - 15 g/day	0.17	0.20
Millwood	Prospective cohort	0 - 20 g/day	20 - 39.9 g/day	0.03	0.03
Millwood	Prospective cohort	0 - 20 g/day	40 - 59.9 g/day	0.10	0.04
Millwood	Prospective cohort	0 - 20 g/day	60 - 90 g/day	0.12	0.04
Millwood	Prospective cohort	0 - 10 g/day	10 - 15 g/day	-0.17	0.07
Millwood	Mendelian randomization	0.6 - 0.6 g/day	2.7 - 2.7 g/day	0.03	0.05
Millwood	Mendelian randomization	0.6 - 0.6 g/day	4.9 - 4.9 g/day	0.08	0.03

Table S9. Results from input studies

Author	Study design	Reference exposure group	Alternative exposure group	Log effect size	Standard error of effect size
Millwood	Mendelian randomization	0.6 - 0.6 g/day	11.1 - 11.1 g/day	-0.06	0.04
Millwood	Mendelian randomization	0.6 - 0.6 g/day	18.6 - 18.6 g/day	0.04	0.03
Millwood	Mendelian randomization	0.6 - 0.6 g/day	36.4 - 36.4 g/day	0.06	0.04
Miyake	Case-control	0 - 0 g/day	0 - 15.8 g/day	-1.20	0.23
Miyake	Case-control	0 - 0 g/day	15.8 - 23.7 g/day	-0.92	0.32
Miyake	Case-control	0 - 0 g/day	0 - 15.8 g/day	-0.69	0.25
Miyake	Case-control	0 - 0 g/day	15.8 - 23.7 g/day	-0.22	0.37
Mukamal	Prospective cohort	0 - 0 g/day	0.1 - 4.9 g/day	-0.01	0.29
Mukamal	Prospective cohort	0 - 0 g/day	5 - 14.9 g/day	-0.49	0.30
Mukamal	Prospective cohort	0 - 0 g/day	15 - 29.9 g/day	-0.97	0.42
Mukamal	Prospective cohort	0 - 0 g/day	30 - 45 g/day	-0.06	0.43
Ng	Prospective cohort	0 - 2.9 g/day	4.3 - 20 g/day	0.26	0.31
Ng	Prospective cohort	0 - 2.9 g/day	20 - 30 g/day	0.73	0.46
Ng	Prospective cohort	0 - 4.3 g/day	5.7 - 30 g/day	0.10	0.14
Ng	Prospective cohort	0 - 4.3 g/day	30 - 45 g/day	-0.05	0.18
Ng	Prospective cohort	0 - 0 g/day	0 - 2.9 g/day	-0.65	0.27
Ng	Prospective cohort	0 - 0 g/day	0 - 4.3 g/day	-0.41	0.13
Oliveira	Case-control	0 - 0 g/day	0.1 - 15 g/day	-0.73	0.22
Oliveira	Case-control	0 - 0 g/day	15.1 - 30 g/day	-0.31	0.26
Oliveira	Case-control	0 - 0 g/day	30 - 45 g/day	-0.02	0.40
Oliveira	Case-control	0.1 - 30 g/day	30.1 - 60 g/day	0.60	0.30
Oliveira	Case-control	0.1 - 30 g/day	60 - 90 g/day	0.77	0.34
Oliveira	Case-control	0.1 - 30 g/day	30.1 - 60 g/day	0.21	0.19
Oliveira	Case-control	0.1 - 30 g/day	60 - 90 g/day	0.80	0.24
Oliveira	Case-control	0 - 0 g/day	0.1 - 30 g/day	-0.79	0.36
Oliveira	Case-control	0 - 0 g/day	0.1 - 30 g/day	-0.26	0.28
Onat	Prospective cohort	0 - 0 g/day	12 - 36 g/day	-0.33	0.19
Onat	Prospective cohort	0 - 0 g/day	36 - 54 g/day	0.83	0.29
Pedersen	Prospective cohort	0 - 1.4 g/day	1.4 - 20 g/day	-0.19	0.10
Pedersen	Prospective cohort	0 - 1.4 g/day	20 - 30 g/day	-0.11	0.11
Pedersen	Prospective cohort	0 - 1.4 g/day	1.4 - 20 g/day	-0.27	0.10
Pedersen	Prospective cohort	0 - 1.4 g/day	20 - 30 g/day	-0.29	0.23
Reddiess	Prospective cohort	0 - 0 g/day	0 - 10 g/day	-1.08	0.26
Reddiess	Prospective cohort	0 - 0 g/day	10 - 20 g/day	-1.20	0.35
Reddiess	Prospective cohort	0 - 0 g/day	20 - 30 g/day	-0.87	0.31
Reddiess	Prospective cohort	0 - 0 g/day	0 - 10 g/day	-0.89	0.26
Reddiess	Prospective cohort	0 - 0 g/day	10 - 20 g/day	-0.94	0.35
Reddiess	Prospective cohort	0 - 0 g/day	20 - 30 g/day	-0.60	0.32
Rehm	Prospective cohort	0 - 0 g/day	0 - 2.9 g/day	0.20	0.16
Rehm	Prospective cohort	0 - 0 g/day	2.9 - 10 g/day	-0.59	0.31
Rehm	Prospective cohort	0 - 0 g/day	11.4 - 20 g/day	-0.51	0.58

Table S9. Results from input studies

Author	Study design	Reference exposure group	Alternative exposure group	Log effect size	Standard error of effect size
Rehm	Prospective cohort	0 - 0 g/day	21.4 - 40 g/day	-0.58	0.72
Rehm	Prospective cohort	0 - 0 g/day	41.4 - 62.1 g/day	1.52	0.60
Rehm	Prospective cohort	0 - 0 g/day	0 - 2.9 g/day	-0.24	0.18
Rehm	Prospective cohort	0 - 0 g/day	2.9 - 10 g/day	-0.31	0.20
Rehm	Prospective cohort	0 - 0 g/day	11.4 - 20 g/day	-0.25	0.25
Rehm	Prospective cohort	0 - 0 g/day	21.4 - 40 g/day	-0.06	0.27
Rehm	Prospective cohort	0 - 0 g/day	41.4 - 62.1 g/day	-0.11	0.31
Renaud	Prospective cohort	0 - 0 g/day	1 - 21 g/day	-0.12	0.25
Renaud	Prospective cohort	0 - 0 g/day	22 - 32 g/day	-0.43	0.23
Renaud	Prospective cohort	0 - 0 g/day	33 - 54 g/day	-0.36	0.20
Renaud	Prospective cohort	0 - 0 g/day	55 - 76 g/day	-0.42	0.26
Ricci	Prospective cohort	0.1 - 4.9 g/day	5 - 14.9 g/day	-0.20	0.05
Ricci	Prospective cohort	0.1 - 4.9 g/day	15 - 29.9 g/day	-0.25	0.06
Ricci	Prospective cohort	0.1 - 4.9 g/day	30 - 59.9 g/day	-0.33	0.06
Ricci	Prospective cohort	0.1 - 4.9 g/day	60 - 90 g/day	-0.39	0.09
Ricci	Prospective cohort	0 - 0.1 g/day	0.1 - 4.9 g/day	-0.12	0.06
Ricci	Prospective cohort	0.1 - 4.9 g/day	5 - 14.9 g/day	-0.21	0.05
Ricci	Prospective cohort	0.1 - 4.9 g/day	15 - 29.9 g/day	-0.29	0.06
Ricci	Prospective cohort	0.1 - 4.9 g/day	30 - 59.9 g/day	-0.34	0.07
Ricci	Prospective cohort	0.1 - 4.9 g/day	60 - 90 g/day	-0.39	0.10
Ricci	Prospective cohort	0 - 0.1 g/day	0.1 - 4.9 g/day	-0.17	0.06
Rimm	Prospective cohort	0 - 0 g/day	0.1 - 5 g/day	-0.01	0.15
Rimm	Prospective cohort	0 - 0 g/day	5.1 - 30 g/day	-0.30	0.14
Rimm	Prospective cohort	0 - 0 g/day	30.1 - 45.1 g/day	-0.63	0.21
Roerecke	Prospective cohort	0 - 0 g/day	0 - 2.5 g/day	0.04	0.27
Roerecke	Prospective cohort	0 - 0 g/day	2.5 - 28 g/day	-0.12	0.26
Roerecke	Prospective cohort	0 - 0 g/day	28 - 56 g/day	-0.62	0.46
Roerecke	Prospective cohort	0 - 0 g/day	56 - 84 g/day	0.22	0.39
Roerecke	Prospective cohort	0 - 0 g/day	0 - 2.5 g/day	0.07	0.22
Roerecke	Prospective cohort	0 - 0 g/day	2.5 - 14 g/day	-0.20	0.34
Roerecke	Prospective cohort	0 - 0 g/day	14 - 21 g/day	-0.25	0.39
Roerecke	Prospective cohort	0 - 0 g/day	0 - 2.5 g/day	-0.09	0.23
Roerecke	Prospective cohort	0 - 0 g/day	2.5 - 28 g/day	-0.25	0.21
Roerecke	Prospective cohort	0 - 0 g/day	28 - 56 g/day	-0.76	0.43
Roerecke	Prospective cohort	0 - 0 g/day	56 - 84 g/day	0.08	0.36
Roerecke	Prospective cohort	0 - 0 g/day	0 - 2.5 g/day	0.12	0.20
Roerecke	Prospective cohort	0 - 0 g/day	2.5 - 14 g/day	-0.14	0.32
Roerecke	Prospective cohort	0 - 0 g/day	14 - 21 g/day	-0.20	0.38
Romelsjö	Prospective cohort	0 - 0 g/day	0.1 - 10 g/day	0.34	0.60
Romelsjö	Prospective cohort	0 - 0 g/day	10 - 30 g/day	0.43	0.62
Romelsjö	Prospective cohort	0 - 0 g/day	30 - 60 g/day	0.34	0.75
Romelsjö	Prospective cohort	0 - 0 g/day	60 - 90 g/day	1.24	0.76
Romelsjö	Prospective cohort	0 - 0 g/day	0.1 - 10 g/day	-0.13	0.18
Romelsjö	Prospective cohort	0 - 0 g/day	10 - 30 g/day	-0.19	0.19

Table S9. Results from input studies

Author	Study design	Reference exposure group	Alternative exposure group	Log effect size	Standard error of effect size
Romelsjö	Prospective cohort	0 - 0 g/day	30 - 60 g/day	-0.31	0.27
Romelsjö	Prospective cohort	0 - 0 g/day	60 - 90 g/day	-0.99	0.46
Romelsjö	Prospective cohort	0 - 0 g/day	0.1 - 10 g/day	-0.07	0.17
Romelsjö	Prospective cohort	0 - 0 g/day	10 - 30 g/day	-0.13	0.18
Romelsjö	Prospective cohort	0 - 0 g/day	30 - 60 g/day	-0.25	0.25
Romelsjö	Prospective cohort	0 - 0 g/day	60 - 90 g/day	-0.48	0.35
Romelsjö	Case-control	0 - 4.9 g/day	5 - 14.9 g/day	-0.31	0.12
Romelsjö	Case-control	0 - 4.9 g/day	15 - 29.9 g/day	-0.26	0.13
Romelsjö	Case-control	0 - 4.9 g/day	30 - 49.9 g/day	-0.20	0.16
Romelsjö	Case-control	0 - 4.9 g/day	50 - 69.9 g/day	-0.42	0.23
Romelsjö	Case-control	0 - 4.9 g/day	70 - 105 g/day	0.06	0.20
Romelsjö	Case-control	0 - 4.9 g/day	5 - 14.9 g/day	-0.36	0.16
Romelsjö	Case-control	0 - 4.9 g/day	15 - 19.9 g/day	-0.14	0.32
Romelsjö	Case-control	0 - 4.9 g/day	20 - 29.9 g/day	-0.71	0.36
Romelsjö	Case-control	0 - 4.9 g/day	30 - 45 g/day	-1.02	0.57
Romelsjö	Case-control	0 - 0 g/day	0 - 4.9 g/day	0.45	0.25
Romelsjö	Case-control	0 - 0 g/day	0 - 4.9 g/day	-0.43	0.23
Romelsjö	Case-control	0 - 0 g/day	0 - 4.9 g/day	0.13	0.17
Romelsjö	Case-control	0 - 0 g/day	0 - 4.9 g/day	-0.60	0.20
Rostron	Prospective cohort	0 - 0.3 g/day	10 - 10 g/day	-0.21	0.14
Rostron	Prospective cohort	0 - 0.3 g/day	20 - 20 g/day	-0.39	0.15
Rostron	Prospective cohort	0 - 0.3 g/day	30 - 45 g/day	-0.08	0.15
Rostron	Prospective cohort	0 - 0.3 g/day	10 - 10 g/day	-0.29	0.15
Rostron	Prospective cohort	0 - 0.3 g/day	20 - 20 g/day	-0.24	0.21
Rostron	Prospective cohort	0 - 0.3 g/day	30 - 45 g/day	0.22	0.22
Rostron	Prospective cohort	0 - 0 g/day	0 - 0.3 g/day	-0.05	0.14
Rostron	Prospective cohort	0 - 0 g/day	0 - 0.3 g/day	-0.43	0.13
Ruidavets	Prospective cohort	1 - 24 g/day	25 - 49 g/day	0.69	0.34
Ruidavets	Prospective cohort	1 - 24 g/day	50 - 74 g/day	-0.11	0.51
Ruidavets	Prospective cohort	1 - 24 g/day	75 - 112.5 g/day	0.69	0.48
Ruidavets	Prospective cohort	1 - 24 g/day	25 - 49 g/day	0.00	0.23
Ruidavets	Prospective cohort	1 - 24 g/day	50 - 74 g/day	-0.11	0.13
Ruidavets	Prospective cohort	1 - 24 g/day	75 - 112.5 g/day	-0.22	0.41
Schooling	Prospective cohort	0 - 0 g/day	0 - 30 g/day	0.00	0.31
Schooling	Prospective cohort	0 - 0 g/day	30 - 45 g/day	-0.58	0.71
Schröder	Case-control	0 - 0 g/day	0 - 20 g/day	-1.51	0.35
Schröder	Case-control	0 - 0 g/day	20 - 30 g/day	-1.97	0.46
Schröder	Case-control	0 - 0 g/day	30 - 45 g/day	-0.69	0.41
Schutte	Prospective cohort	0 - 0 g/day	1.1 - 1.7 g/day	0.01	0.06
Schutte	Prospective cohort	0 - 0 g/day	1.1 - 1.7 g/day	-0.08	0.06
Schutte	Prospective cohort	0 - 0 g/day	1.1 - 16 g/day	-0.15	0.15
Schutte	Prospective cohort	0 - 0 g/day	16 - 24 g/day	-0.01	0.12
Scragg	Case-control	0 - 0 g/day	1 - 9 g/day	-0.92	0.22
Scragg	Case-control	0 - 0 g/day	10 - 34 g/day	-0.51	0.21

Table S9. Results from input studies

Author	Study design	Reference exposure group	Alternative exposure group	Log effect size	Standard error of effect size
Scragg	Case-control	0 - 0 g/day	34 - 51 g/day	-0.51	0.21
Scragg	Case-control	0 - 0 g/day	1 - 9 g/day	-0.69	0.38
Scragg	Case-control	0 - 0 g/day	10 - 34 g/day	0.26	0.32
Scragg	Case-control	0 - 0 g/day	34 - 51 g/day	-0.92	0.67
Sempos	Prospective cohort	0 - 0 g/day	0 - 2.9 g/day	-0.04	0.08
Sempos	Prospective cohort	0 - 0 g/day	2.9 - 10 g/day	-0.51	0.15
Sempos	Prospective cohort	0 - 0 g/day	10 - 20 g/day	-0.34	0.27
Sempos	Prospective cohort	0 - 0 g/day	20 - 30 g/day	0.17	0.10
Sempos	Prospective cohort	0 - 0 g/day	0 - 2.9 g/day	-0.11	0.10
Sempos	Prospective cohort	0 - 0 g/day	2.9 - 10 g/day	-0.25	0.11
Sempos	Prospective cohort	0 - 0 g/day	10 - 20 g/day	-0.25	0.15
Sempos	Prospective cohort	0 - 0 g/day	20 - 30 g/day	-0.16	0.14
Shaper	Prospective cohort	0 - 1.3 g/day	1.3 - 19.3 g/day	0.00	0.26
Shaper	Prospective cohort	0 - 1.3 g/day	20.6 - 54 g/day	-0.33	0.27
Shaper	Prospective cohort	0 - 1.3 g/day	54 - 81 g/day	-0.19	0.34
Shaper	Prospective cohort	0 - 0 g/day	0 - 1.3 g/day	0.03	0.43
Shiu	Prospective cohort	0 - 0 g/day	13 - 13 g/day	0.00	0.04
Shiu	Prospective cohort	0 - 0 g/day	13 - 13 g/day	-0.01	0.04
Shiu	Mendelian randomization	0 - 0 g/day	13 - 13 g/day	-0.01	0.17
Shiu	Mendelian randomization	0 - 0 g/day	13 - 13 g/day	0.42	0.20
Simons	Prospective cohort	0 - 0 g/day	1.4 - 10 g/day	-0.14	0.16
Simons	Prospective cohort	0 - 0 g/day	11.4 - 20 g/day	-0.27	0.19
Simons	Prospective cohort	0 - 0 g/day	21.4 - 40 g/day	0.16	0.21
Simons	Prospective cohort	0 - 0 g/day	40 - 60 g/day	-0.21	0.30
Simons	Prospective cohort	0 - 0 g/day	1.4 - 10 g/day	-0.27	0.14
Simons	Prospective cohort	0 - 0 g/day	11.4 - 20 g/day	0.02	0.21
Simons	Prospective cohort	0 - 0 g/day	21.4 - 40 g/day	-0.42	0.52
Skov-Ettrup	Prospective cohort	0 - 0 g/day	0 - 36 g/day	0.28	0.11
Skov-Ettrup	Prospective cohort	0 - 0 g/day	0 - 24 g/day	0.55	0.10
Snow	Prospective cohort	0 - 0 g/day	0.7 - 5.8 g/day	0.51	1.08
Snow	Prospective cohort	0 - 0 g/day	5.8 - 18.1 g/day	-0.56	1.15
Snow	Prospective cohort	0 - 0 g/day	18.1 - 27.1 g/day	-0.82	1.21
Snow	Prospective cohort	0 - 0 g/day	0.7 - 5.8 g/day	-0.02	0.45
Snow	Prospective cohort	0 - 0 g/day	5.8 - 18.1 g/day	-0.29	0.49
Snow	Prospective cohort	0 - 0 g/day	18.1 - 27.1 g/day	-1.27	0.63
Snow	Prospective cohort	0 - 0 g/day	0.7 - 2.9 g/day	0.12	1.42
Snow	Prospective cohort	0 - 0 g/day	2.9 - 9.2 g/day	1.08	1.18
Snow	Prospective cohort	0 - 0 g/day	9.2 - 13.7 g/day	0.67	1.24
Snow	Prospective cohort	0 - 0 g/day	0.7 - 2.9 g/day	0.30	0.50
Snow	Prospective cohort	0 - 0 g/day	2.9 - 9.2 g/day	0.75	0.48
Snow	Prospective cohort	0 - 0 g/day	9.2 - 13.7 g/day	-0.82	0.79
Song	Prospective cohort	0 - 6 g/day	6 - 12 g/day	-0.08	0.06

Table S9. Results from input studies

Author	Study design	Reference exposure group	Alternative exposure group	Log effect size	Standard error of effect size
Song	Prospective cohort	0 - 6 g/day	12 - 24 g/day	-0.16	0.06
Song	Prospective cohort	0 - 6 g/day	24 - 36 g/day	-0.29	0.10
Song	Prospective cohort	0 - 6 g/day	36 - 48 g/day	-0.36	0.12
Song	Prospective cohort	0 - 0 g/day	0 - 6 g/day	-0.19	0.06
Song	Prospective cohort	0 - 0 g/day	0 - 6 g/day	-0.19	0.06
Song	Prospective cohort	0 - 0 g/day	6 - 12 g/day	-0.26	0.07
Song	Prospective cohort	0 - 0 g/day	12 - 24 g/day	-0.34	0.07
Song	Prospective cohort	0 - 0 g/day	24 - 36 g/day	-0.48	0.10
Song	Prospective cohort	0 - 0 g/day	36 - 48 g/day	-0.54	0.12
Streppel	Prospective cohort	0 - 0 g/day	0 - 20 g/day	-0.08	0.16
Streppel	Prospective cohort	0 - 0 g/day	20 - 30 g/day	-0.22	0.25
Suhonen	Prospective cohort	0 - 0 g/day	0 - 6.7 g/day	0.98	0.49
Suhonen	Prospective cohort	0 - 0 g/day	6.7 - 10 g/day	0.46	0.57
Suhonen	Prospective cohort	0 - 0 g/day	0 - 6.7 g/day	0.25	0.37
Suhonen	Prospective cohort	0 - 0 g/day	6.7 - 10 g/day	0.17	0.38
Suhonen	Prospective cohort	0 - 0 g/day	0 - 6.7 g/day	0.25	0.35
Suhonen	Prospective cohort	0 - 0 g/day	6.7 - 10 g/day	0.66	0.33
Tavani	Case-control	0 - 0 g/day	0 - 24 g/day	-0.22	0.13
Tavani	Case-control	0 - 0 g/day	48 - 72 g/day	0.18	0.33
Tavani	Case-control	0 - 10 g/day	10 - 20 g/day	-0.19	0.16
Tavani	Case-control	0 - 10 g/day	20 - 30 g/day	-0.33	0.13
Thun	Prospective cohort	0 - 0 g/day	0 - 7.7 g/day	-0.36	0.03
Thun	Prospective cohort	0 - 0 g/day	12 - 12 g/day	-0.36	0.07
Thun	Prospective cohort	0 - 0 g/day	24 - 36 g/day	-0.36	0.04
Thun	Prospective cohort	0 - 0 g/day	48 - 72 g/day	-0.51	0.05
Thun	Prospective cohort	0 - 0 g/day	0 - 7.7 g/day	-0.36	0.07
Thun	Prospective cohort	0 - 0 g/day	12 - 12 g/day	-0.51	0.09
Thun	Prospective cohort	0 - 0 g/day	24 - 36 g/day	-0.51	0.09
Thun	Prospective cohort	0 - 0 g/day	48 - 72 g/day	-0.51	0.09
Tolstrup	Prospective cohort	1.7 - 10.3 g/day	12 - 22.3 g/day	-0.19	0.08
Tolstrup	Prospective cohort	1.7 - 10.3 g/day	24 - 34.3 g/day	-0.17	0.09
Tolstrup	Prospective cohort	1.7 - 10.3 g/day	36 - 46.3 g/day	-0.27	0.10
Tolstrup	Prospective cohort	1.7 - 10.3 g/day	48 - 58.3 g/day	-0.42	0.14
Tolstrup	Prospective cohort	1.7 - 10.3 g/day	60 - 90 g/day	-0.43	0.11
Tolstrup	Prospective cohort	1.7 - 10.3 g/day	12 - 22.3 g/day	-0.19	0.10
Tolstrup	Prospective cohort	1.7 - 10.3 g/day	24 - 34.3 g/day	-0.33	0.14
Tolstrup	Prospective cohort	1.7 - 10.3 g/day	36 - 46.3 g/day	-0.36	0.19
Tolstrup	Prospective cohort	1.7 - 10.3 g/day	48 - 72 g/day	-0.46	0.24
Tolstrup	Prospective cohort	0 - 0 g/day	1.7 - 10.3 g/day	-0.49	0.17
Tolstrup	Prospective cohort	0 - 0 g/day	1.7 - 10.3 g/day	-0.08	0.21
Wannamethee	Prospective cohort	0 - 1.3 g/day	1.3 - 19.3 g/day	-0.15	0.41
Wannamethee	Prospective cohort	0 - 1.3 g/day	20.6 - 54 g/day	-0.34	0.47
Wannamethee	Prospective cohort	0 - 1.3 g/day	54 - 81 g/day	0.31	0.61
Wannamethee	Prospective cohort	0 - 1.3 g/day	54 - 81 g/day	-0.29	0.12

Table S9. Results from input studies

Author	Study design	Reference exposure group	Alternative exposure group	Log effect size	Standard error of effect size
Wannamethee	Prospective cohort	0 - 1.3 g/day	1.3 - 19.3 g/day	-0.27	0.09
Wannamethee	Prospective cohort	0 - 1.3 g/day	20.6 - 54 g/day	-0.25	0.09
Wannamethee	Prospective cohort	0 - 0 g/day	0 - 1.3 g/day	-0.03	0.14
Wilkins	Prospective cohort	0 - 0 g/day	1.4 - 1.4 g/day	-0.51	0.50
Wilkins	Prospective cohort	0 - 0 g/day	2.9 - 12.9 g/day	-0.92	0.38
Wilkins	Prospective cohort	0 - 0 g/day	14.3 - 21.4 g/day	-0.22	0.51
Wilkins	Prospective cohort	0 - 0 g/day	1.4 - 1.4 g/day	0.53	0.46
Wilkins	Prospective cohort	0 - 0 g/day	2.9 - 20 g/day	0.47	0.34
Wilkins	Prospective cohort	0 - 0 g/day	21.4 - 32.1 g/day	-0.36	0.47
Wilkins	Prospective cohort	0 - 0 g/day	0 - 0 g/day	-0.36	0.30
Wilkins	Prospective cohort	0 - 0 g/day	0 - 0 g/day	0.41	0.31
Yang	Prospective cohort	0 - 0 g/day	0 - 20 g/day	-0.01	0.10
Yang	Prospective cohort	0 - 0 g/day	20 - 39.9 g/day	-0.09	0.10
Yang	Prospective cohort	0 - 0 g/day	40 - 59.9 g/day	0.16	0.10
Yang	Prospective cohort	0 - 0 g/day	60 - 99.9 g/day	0.11	0.13
Yi	Prospective cohort	0 - 0 g/day	0 - 10 g/day	0.03	0.62
Yi	Prospective cohort	0 - 0 g/day	10 - 72 g/day	-0.97	0.69
Yi	Prospective cohort	0 - 0 g/day	72 - 108 g/day	-0.06	0.81
Younis	Prospective cohort	0 - 1.1 g/day	2.3 - 6.9 g/day	-0.34	0.19
Younis	Prospective cohort	0 - 1.1 g/day	6.9 - 10.3 g/day	-0.31	0.16
Yusuf	Prospective cohort	0 - 0 g/day	0 - 10 g/day	-0.31	0.07
Yusuf	Prospective cohort	0 - 0 g/day	11.4 - 24.2 g/day	-0.22	0.11
Yusuf	Prospective cohort	0 - 0 g/day	24.2 - 36.3 g/day	-0.34	0.16
Zhang	Prospective cohort	0 - 0 g/day	0 - 10 g/day	-0.22	0.11
Zhang	Prospective cohort	0 - 0 g/day	10 - 30 g/day	0.02	0.10
Zhang	Prospective cohort	0 - 0 g/day	30 - 45 g/day	-0.29	0.12
Zhang	Prospective cohort	0 - 0 g/day	0 - 20 g/day	0.01	0.10
Zhang	Prospective cohort	0 - 0 g/day	20 - 40 g/day	-0.27	0.11
Zhang	Prospective cohort	0 - 0 g/day	40 - 60 g/day	-0.15	0.11
Zhou	Case-control	0 - 1.4 g/day	1.6 - 9.4 g/day	0.15	0.27
Zhou	Case-control	0 - 1.4 g/day	11 - 20.4 g/day	0.58	0.13
Zhou	Case-control	0 - 1.4 g/day	20.4 - 30.6 g/day	0.78	0.20

Section 7: Risk curve details

Table S10. Relative risks across exposure range

	Alcohol consumption (g/day)	RR (95% UI with between-study heterogeneity)	RR (95% UI without between-study heterogeneity)
Ischemic heart disease	10	0.76 (0.57, 1.01)	0.76 (0.74, 0.78)
	20	0.70 (0.48, 1.01)	0.70 (0.67, 0.72)
	30	0.70 (0.48, 1.01)	0.70 (0.67, 0.72)
	40	0.71 (0.50, 1.01)	0.71 (0.69, 0.73)
	50	0.73 (0.53, 1.01)	0.73 (0.71, 0.75)
	60	0.76 (0.57, 1.01)	0.76 (0.74, 0.78)
	70	0.80 (0.63, 1.01)	0.80 (0.78, 0.82)
	80	0.85 (0.72, 1.01)	0.85 (0.84, 0.86)
	90	0.91 (0.82, 1.00)	0.91 (0.90, 0.92)
	100	0.96 (0.92, 1.00)	0.96 (0.96, 0.96)
Ischemic heart disease morbidity	10	0.76 (0.51, 1.14)	0.76 (0.73, 0.80)
	20	0.67 (0.37, 1.21)	0.67 (0.63, 0.72)
	30	0.66 (0.36, 1.22)	0.66 (0.62, 0.71)
	40	0.68 (0.39, 1.20)	0.68 (0.64, 0.73)
	50	0.71 (0.42, 1.18)	0.71 (0.67, 0.75)
	60	0.72 (0.44, 1.17)	0.72 (0.68, 0.76)
	70	0.73 (0.45, 1.17)	0.73 (0.69, 0.77)
	80	0.73 (0.45, 1.17)	0.73 (0.69, 0.77)
	90	0.72 (0.44, 1.17)	0.72 (0.68, 0.76)
	100	N/A	N/A
Ischemic heart disease morbidity – Females	10	0.77 (0.56, 1.04)	0.77 (0.66, 0.89)
	20	0.76 (0.55, 1.04)	0.76 (0.65, 0.89)
	30	0.81 (0.64, 1.03)	0.81 (0.73, 0.91)
	40	0.85 (0.70, 1.03)	0.85 (0.77, 0.93)
	50	0.86 (0.72, 1.02)	0.86 (0.79, 0.93)
	60	0.86 (0.72, 1.02)	0.86 (0.79, 0.93)
	70	0.86 (0.72, 1.02)	0.86 (0.79, 0.93)
	80	0.86 (0.72, 1.02)	0.86 (0.79, 0.93)
	90	0.86 (0.71, 1.02)	0.86 (0.78, 0.93)
	100	0.86 (0.71, 1.02)	0.86 (0.78, 0.93)
Ischemic heart disease morbidity – Males	10	0.63 (0.44, 0.91)	0.63 (0.54, 0.74)
	20	0.54 (0.33, 0.87)	0.54 (0.44, 0.66)
	30	0.58 (0.38, 0.89)	0.58 (0.49, 0.70)
	40	0.67 (0.48, 0.91)	0.67 (0.58, 0.76)
	50	0.73 (0.58, 0.93)	0.73 (0.66, 0.81)
	60	0.78 (0.64, 0.95)	0.78 (0.72, 0.85)
	70	0.81 (0.68, 0.95)	0.81 (0.75, 0.87)
	80	0.82 (0.70, 0.96)	0.82 (0.77, 0.88)
	90	N/A	N/A
	100	N/A	N/A
Ischemic heart disease mortality	10	0.81 (0.55, 1.21)	0.81 (0.77, 0.86)

Table S10. Relative risks across exposure range

	Alcohol consumption (g/day)	RR (95% UI with between-study heterogeneity)	RR (95% UI without between-study heterogeneity)
	20	0.75 (0.43, 1.31)	0.75 (0.70, 0.80)
	30	0.74 (0.42, 1.31)	0.74 (0.69, 0.80)
	40	0.77 (0.46, 1.27)	0.77 (0.72, 0.82)
	50	0.79 (0.51, 1.23)	0.79 (0.75, 0.84)
	60	0.81 (0.54, 1.21)	0.81 (0.77, 0.85)
	70	0.82 (0.57, 1.19)	0.82 (0.79, 0.86)
	80	0.83 (0.58, 1.19)	0.83 (0.79, 0.87)
	90	0.83 (0.58, 1.19)	0.83 (0.79, 0.87)
	100	N/A	N/A
Ischemic heart disease mortality – Females	10	0.86 (0.55, 1.33)	0.86 (0.75, 0.98)
	20	0.81 (0.44, 1.48)	0.81 (0.67, 0.97)
	30	0.83 (0.48, 1.42)	0.83 (0.70, 0.98)
	40	0.85 (0.53, 1.35)	0.85 (0.74, 0.98)
	50	0.86 (0.56, 1.32)	0.86 (0.75, 0.98)
	60	0.86 (0.56, 1.32)	0.86 (0.75, 0.98)
	70	N/A	N/A
	80	N/A	N/A
	90	N/A	N/A
	100	N/A	N/A
Ischemic heart disease mortality – Males	10	0.76 (0.64, 0.91)	0.76 (0.72, 0.80)
	20	0.70 (0.56, 0.88)	0.70 (0.65, 0.75)
	30	0.74 (0.61, 0.90)	0.74 (0.70, 0.79)
	40	0.81 (0.71, 0.93)	0.81 (0.78, 0.84)
	50	0.87 (0.80, 0.95)	0.87 (0.85, 0.89)
	60	0.91 (0.86, 0.97)	0.91 (0.90, 0.93)
	70	0.94 (0.91, 0.98)	0.94 (0.93, 0.95)
	80	0.96 (0.93, 0.98)	0.96 (0.95, 0.96)
	90	N/A	N/A
	100	N/A	N/A
Case-control studies	10	0.76 (0.64, 0.90)	0.76 (0.72, 0.81)
	20	0.66 (0.50, 0.85)	0.66 (0.60, 0.72)
	30	0.68 (0.53, 0.86)	0.68 (0.62, 0.74)
	40	0.79 (0.68, 0.92)	0.79 (0.75, 0.83)
	50	0.91 (0.85, 0.96)	0.91 (0.89, 0.93)
	60	0.99 (0.98, 1.00)	0.99 (0.99, 0.99)
	70	1.04 (1.01, 1.07)	1.04 (1.03, 1.05)
	80	1.07 (1.02, 1.11)	1.07 (1.05, 1.08)
	90	1.08 (1.03, 1.13)	1.08 (1.06, 1.10)
	100	1.08 (1.03, 1.14)	1.08 (1.07, 1.10)
Cohort studies	10	0.76 (0.58, 1.00)	0.76 (0.74, 0.78)
	20	0.69 (0.47, 1.01)	0.69 (0.66, 0.71)
	30	0.69 (0.47, 1.01)	0.69 (0.67, 0.72)
	40	0.70 (0.49, 1.00)	0.70 (0.68, 0.73)

Table S10. Relative risks across exposure range

	Alcohol consumption (g/day)	RR (95% UI with between-study heterogeneity)	RR (95% UI without between-study heterogeneity)
	50	0.72 (0.52, 1.00)	0.72 (0.70, 0.75)
	60	0.75 (0.56, 1.00)	0.75 (0.73, 0.77)
	70	0.78 (0.61, 1.00)	0.78 (0.76, 0.80)
	80	0.83 (0.68, 1.00)	0.83 (0.81, 0.84)
	90	0.89 (0.79, 1.00)	0.89 (0.88, 0.90)
	100	0.96 (0.93, 1.00)	0.96 (0.96, 0.97)
Mendelian randomization studies	10	1.00 (0.98, 1.02)	1.00 (0.99, 1.01)
	20	1.00 (0.94, 1.06)	1.00 (0.97, 1.03)
	30	1.00 (0.88, 1.14)	1.00 (0.94, 1.07)
	40	1.00 (0.80, 1.24)	1.00 (0.90, 1.12)
	50	N/A	N/A
	60	N/A	N/A
	70	N/A	N/A
	80	N/A	N/A
	90	N/A	N/A
	100	N/A	N/A
Myocardial infarction	10	0.68 (0.49, 0.97)	0.68 (0.65, 0.72)
	20	0.66 (0.45, 0.96)	0.66 (0.62, 0.70)
	30	0.69 (0.49, 0.97)	0.69 (0.65, 0.73)
	40	0.71 (0.52, 0.97)	0.71 (0.67, 0.75)
	50	0.73 (0.54, 0.97)	0.73 (0.69, 0.76)
	60	0.74 (0.56, 0.97)	0.74 (0.71, 0.77)
	70	0.75 (0.57, 0.97)	0.75 (0.71, 0.78)
	80	0.75 (0.58, 0.97)	0.75 (0.72, 0.78)
	90	0.75 (0.58, 0.98)	0.75 (0.72, 0.78)
	100	0.76 (0.59, 0.98)	0.76 (0.73, 0.79)
Myocardial infarction morbidity	10	0.65 (0.49, 0.87)	0.65 (0.60, 0.72)
	20	0.63 (0.47, 0.86)	0.63 (0.57, 0.70)
	30	0.69 (0.54, 0.88)	0.69 (0.64, 0.75)
	40	0.75 (0.62, 0.91)	0.75 (0.70, 0.80)
	50	0.80 (0.68, 0.93)	0.80 (0.76, 0.84)
	60	0.83 (0.74, 0.94)	0.83 (0.80, 0.87)
	70	0.86 (0.78, 0.95)	0.86 (0.83, 0.89)
	80	0.88 (0.81, 0.96)	0.88 (0.85, 0.90)
	90	0.89 (0.82, 0.96)	0.89 (0.86, 0.91)
	100	0.88 (0.81, 0.96)	0.88 (0.85, 0.90)
Myocardial infarction mortality	10	0.86 (0.52, 1.41)	0.86 (0.78, 0.94)
	20	0.79 (0.37, 1.70)	0.79 (0.69, 0.90)
	30	0.76 (0.32, 1.83)	0.76 (0.65, 0.89)
	40	0.75 (0.30, 1.88)	0.75 (0.64, 0.89)
	50	0.75 (0.29, 1.92)	0.75 (0.63, 0.88)
	60	0.74 (0.28, 1.96)	0.74 (0.62, 0.88)
	70	0.74 (0.27, 1.99)	0.74 (0.62, 0.88)

Table S10. Relative risks across exposure range

	Alcohol consumption (g/day)	RR (95% UI with between-study heterogeneity)	RR (95% UI without between-study heterogeneity)
	80	0.73 (0.26, 2.02)	0.73 (0.61, 0.88)
	90	N/A	N/A
	100	N/A	N/A

Note. N/A = not available, RR = relative risk, UI = uncertainty interval.

Section 8. Details on statistical methods

Table S11. MR-BRT splines and prior specifications

	Spline degree, number of interior knots	Priors & constraints
Ischemic heart disease	Quadratic, 2 interior knots	Gaussian max derivative prior on the right tail (0, 0.001)
Morbidity	Quadratic, 2 interior knots	Right linear tail, Gaussian max derivative prior on the right tail (0, 0.001)
Females	Quadratic, 2 interior knots	Right linear tail, Gaussian max derivative prior on the right tail (0, 0.001)
Males	Quadratic, 2 interior knots	Right linear tail, Gaussian max derivative prior on the right tail (0, 0.001)
Mortality	Quadratic, 2 interior knots	Right linear tail, Gaussian max derivative prior on the right tail (0, 0.001)
Females	Quadratic, 2 interior knots	Right linear tail, Gaussian max derivative prior on the right tail (0, 0.001)
Males	Quadratic, 2 interior knots	Right linear tail, Gaussian max derivative prior on the right tail (0, 0.001)
Case-control studies	Quadratic, 2 interior knots	Right linear tail, Gaussian max derivative prior on the right tail (0, 0.001)
Cohort studies	Quadratic, 2 interior knots	Gaussian max derivative prior on the right tail (0, 0.001)
Mendelian randomization studies	Quadratic, 2 interior knots	Left and right linear tail, Gaussian max derivative prior on the right tail (0, 0.001)
Myocardial infarction	Quadratic, 2 interior knots	Gaussian max derivative prior on the right tail (0, 0.001)
Morbidity	Quadratic, 2 interior knots	Gaussian max derivative prior on the right tail (0, 0.001)
Mortality	Quadratic, 2 interior knots	Right linear tail, Gaussian max derivative prior on the right tail (0, 0.001)

Table S12. Bias covariates and estimated parameters

	Selected bias covariates	Gamma solution (mean and SD)
Ischemic heart disease	cov_adjusted_0, cov_adjusted_1, cov_adjusted_3, cov_outcome_selfreport, cov_sick_quitters, cov_non_drinker, cov_older, cov_cholesterol, cov_apolipoprotein, cov_ihd	0.19 (0.06)
Morbidity	cov_bmi, cov_ihd, cov_mi, cov_outcome_selfreport	0.47 (0.2)
Females	cov_rep_prevalent_disease, cov_ihd, cov_adjusted_2	0.00000001 (0.74)
Males	cov_rep_prevalent_disease	0.00000003 (0.24)
Mortality	cov_rep_geography, cov_adjusted_1	0.41 (0.2)
Females	None	0.07 (0.16)
Males	None	0.02 (0.04)
Case-control studies	None	0.00000001 (0.05)
Cohort studies	cov_apolipoprotein, cov_ihd	0.18 (0.06)
Mendelian randomization studies	cov_mortality, cov_adjusted_1, cov_adjusted_2	23.44 (18.83)
Myocardial infarction	cov_mortality	0.09 (0.05)
Morbidity	None	0.000000004 (0.03)
Mortality	cov_rep_geography	0.6 (0.43)

Note. SD = standard deviation.

Section 9: Subanalyses

Figure S2a-c. Relative risk of alcohol consumption on morbidity of ischemic heart disease, for both sexes and by sex, based on data from all conventional observational (cohort and case-control) studies

a. Ischemic heart disease morbidity – Both sexes

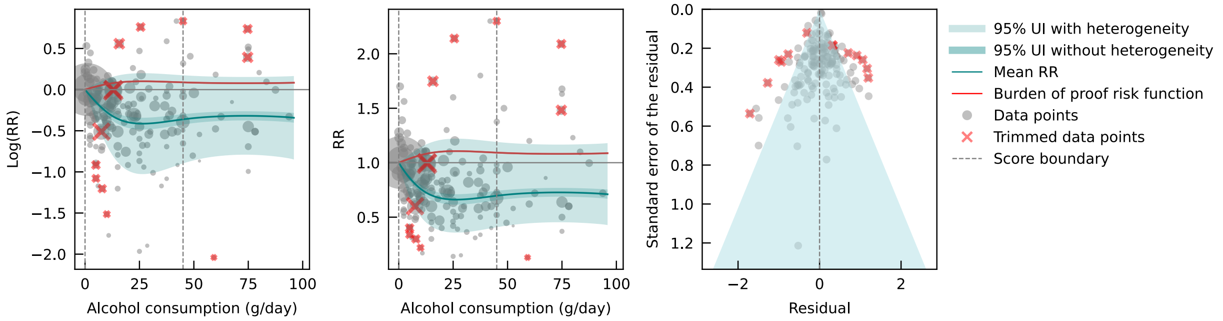

b. Ischemic heart disease morbidity – Females

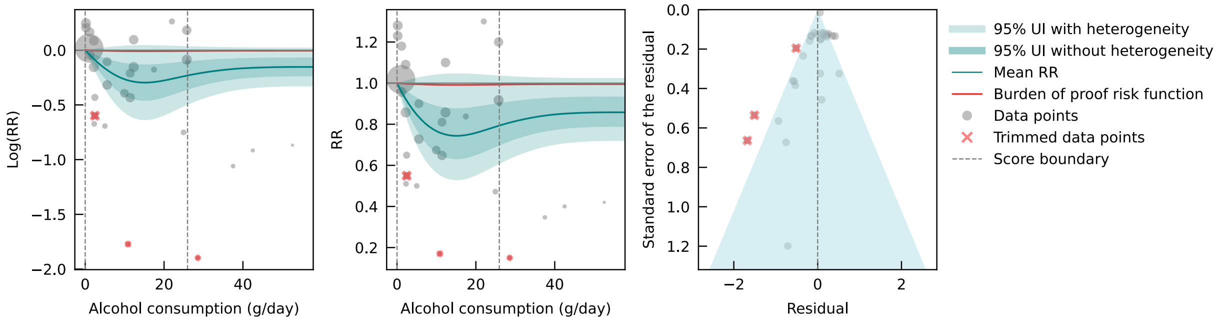

c. Ischemic heart disease morbidity – Males

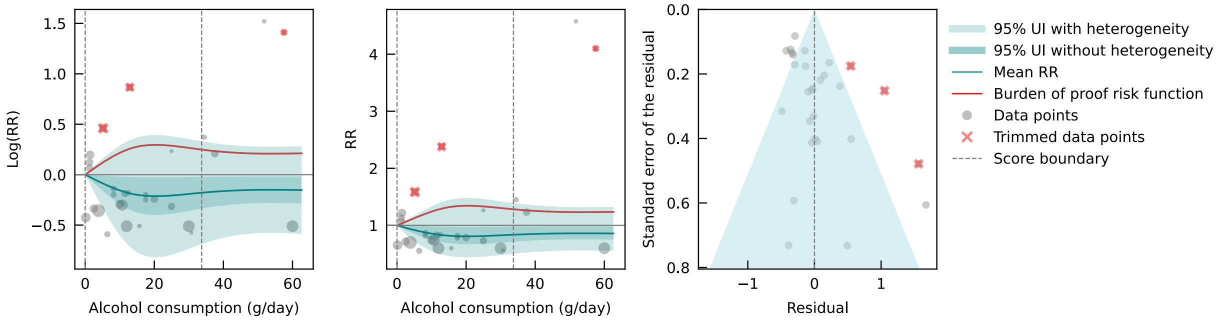

Note. The panels show the log(relative risk) function, the relative risk function, and a modified funnel plot showing the residuals (relative to 0) on the x axis and the estimated standard error of the residuals that includes the reported standard error and between-study heterogeneity on the y axis. RR = relative risk, UI = uncertainty interval. Source data are provided as a Source Data file.

Figure S3a-c. Relative risk of alcohol consumption on mortality of ischemic heart disease, for both sexes by sex, based on data from all conventional observational (cohort and case-control) studies

a. Ischemic heart disease mortality – Both sexes

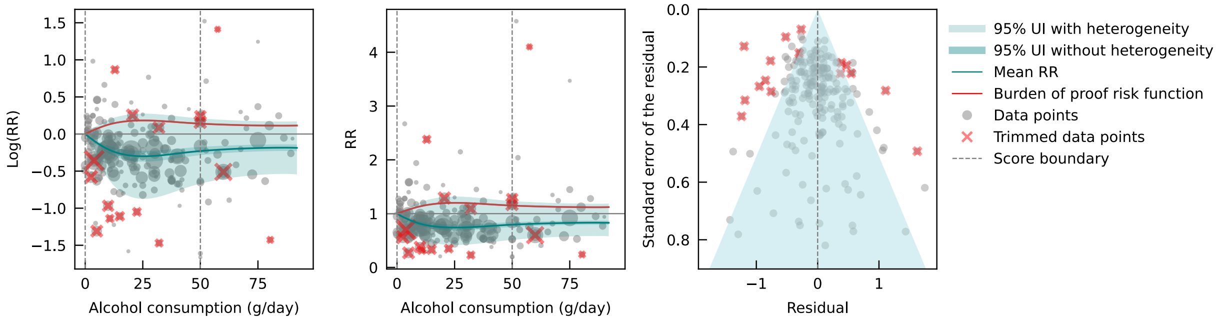

b. Ischemic heart disease mortality – Females

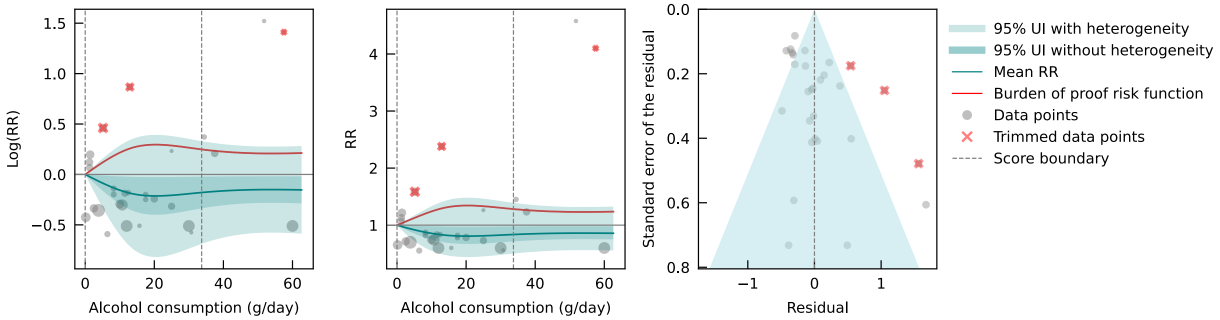

c. Ischemic heart disease mortality – Males

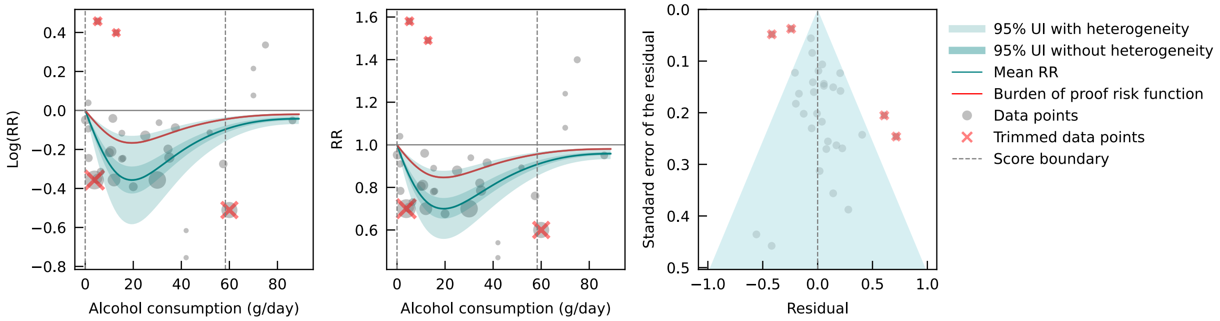

Note. The panels show the log(relative risk) function, the relative risk function, and a modified funnel plot showing the residuals (relative to 0) on the x axis and the estimated standard error that includes the reported standard error and between-study heterogeneity on the y axis. RR = relative risk, UI = uncertainty interval. Source data are provided as a Source Data file.

Figure S4. Relative risk of alcohol consumption on mortality of myocardial infarction, overall and by endpoint, based on data from all conventional observational (cohort and case-control) studies

a. Myocardial infarction (morbidity and mortality)

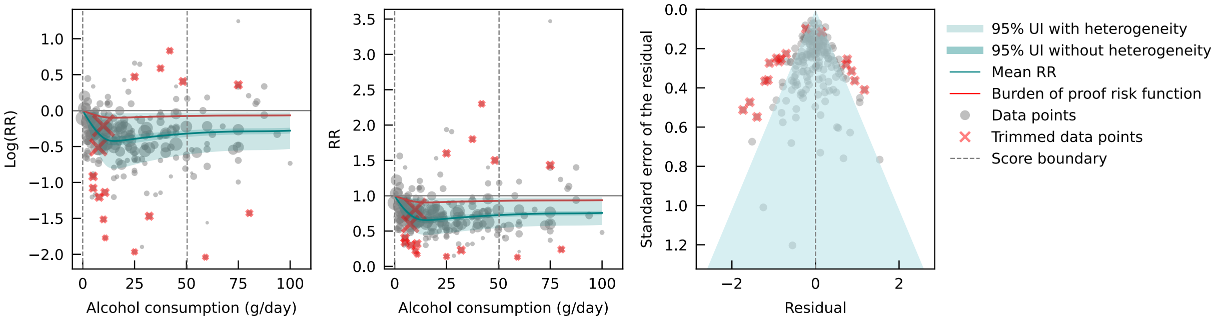

b. Myocardial infarction morbidity

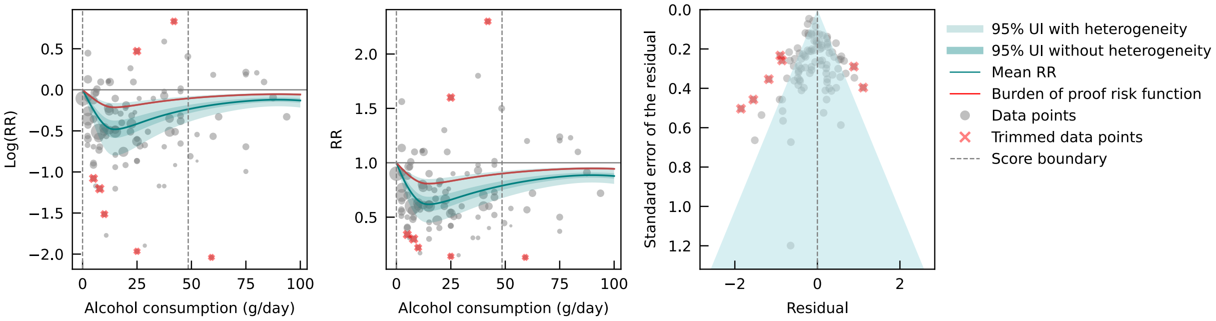

c. Myocardial infarction mortality

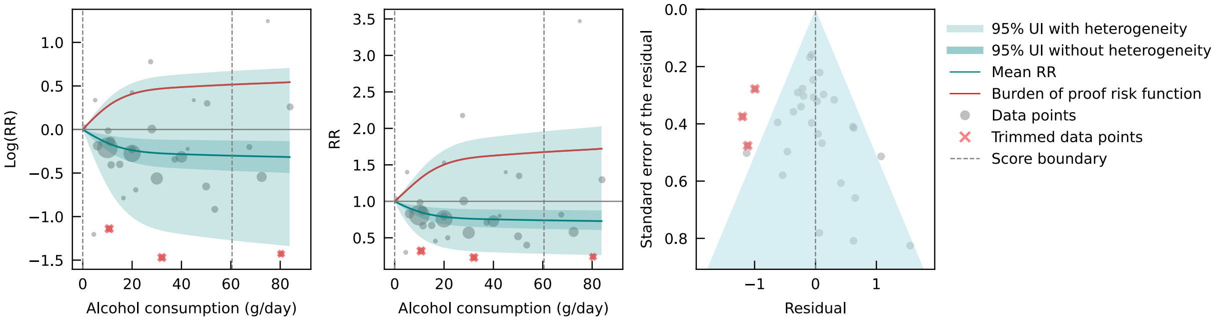

Note. The panels show the log(relative risk) function, the relative risk function, and a modified funnel plot showing the residuals (relative to 0) on the x axis and the estimated standard error that includes the reported standard error and between-study heterogeneity on the y axis. RR = relative risk, UI = uncertainty interval. Source data are provided as a Source Data file.

Section 10: Sensitivity analyses

Section 10.1: Results without trimming

Figure S5a-l. Relative risk of alcohol consumption on ischemic heart disease or subtypes, without trimming any data

a. Ischemic heart disease (morbidity and mortality)

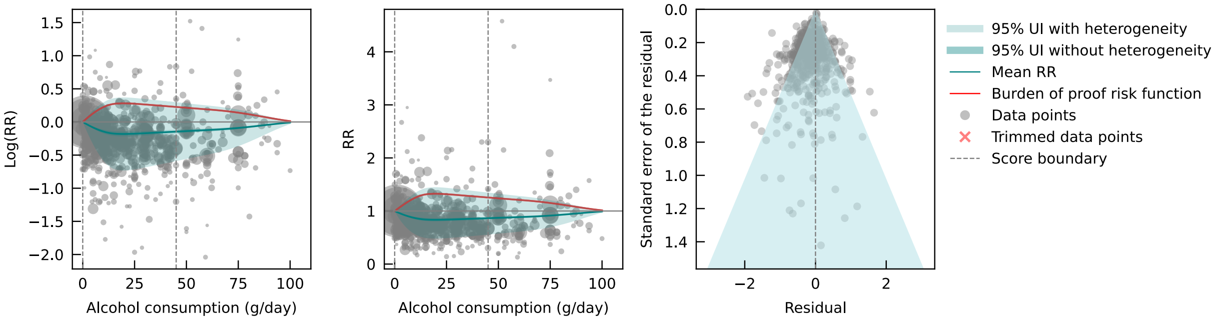

b. Ischemic heart disease morbidity – Both sexes

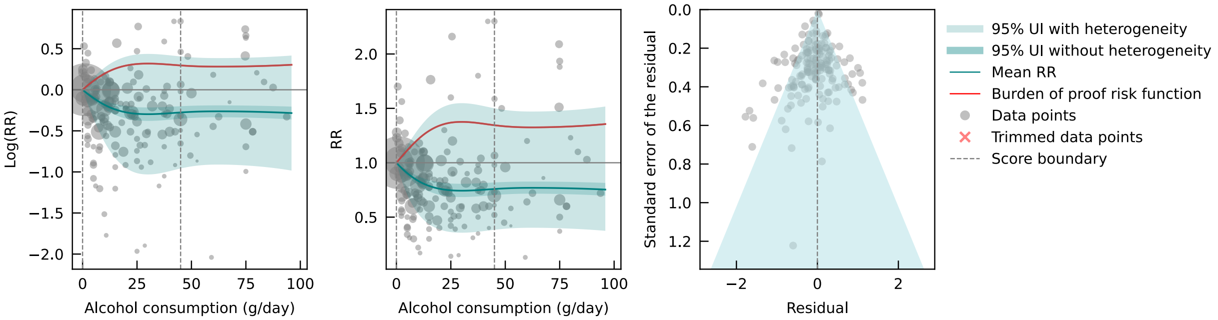

c. Ischemic heart disease morbidity – Females

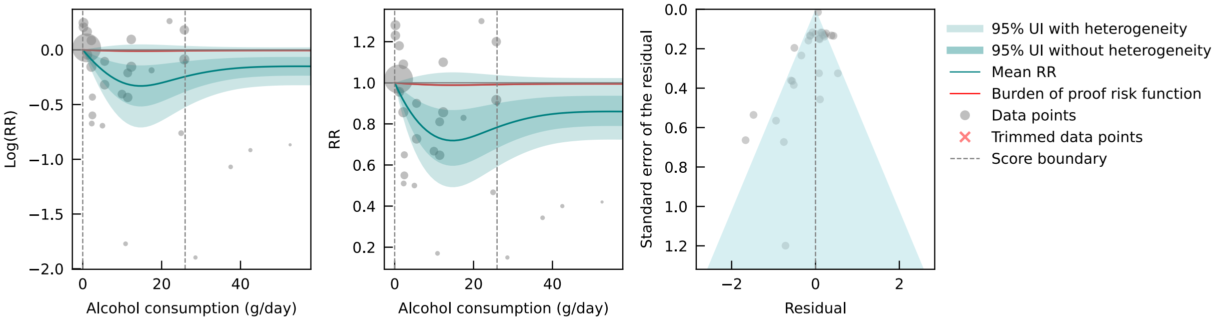

Note. The panels show the log(relative risk) function, the relative risk function, and a modified funnel plot showing the residuals (relative to 0) on the x axis and the estimated standard error that includes the reported standard error and between-study heterogeneity on the y axis. RR = relative risk, UI = uncertainty interval. Source data are provided as a Source Data file.

d. Ischemic heart disease morbidity – Males

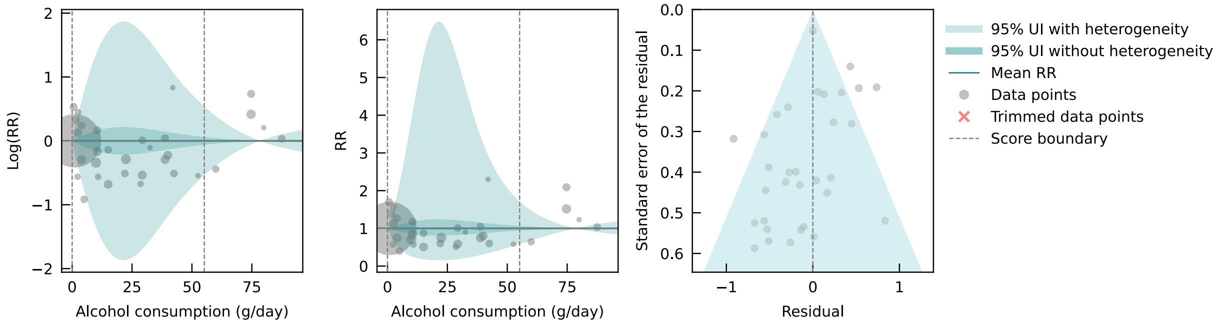

e. Ischemic heart disease mortality – Both sexes

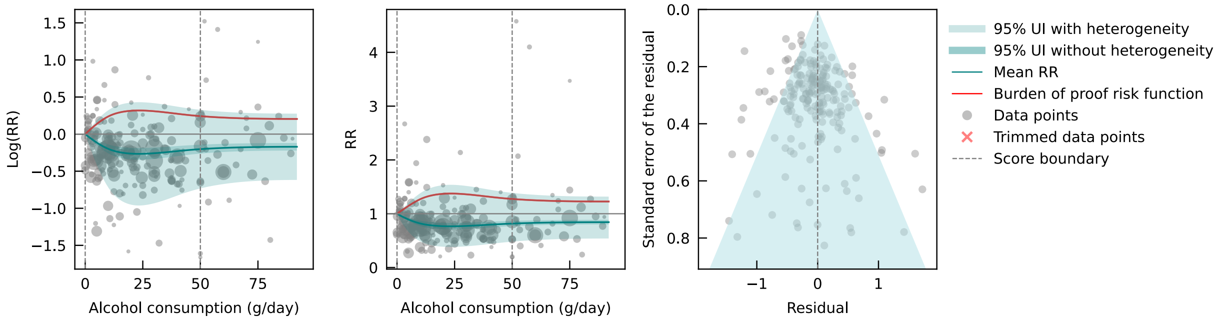

f. Ischemic heart disease mortality – Females

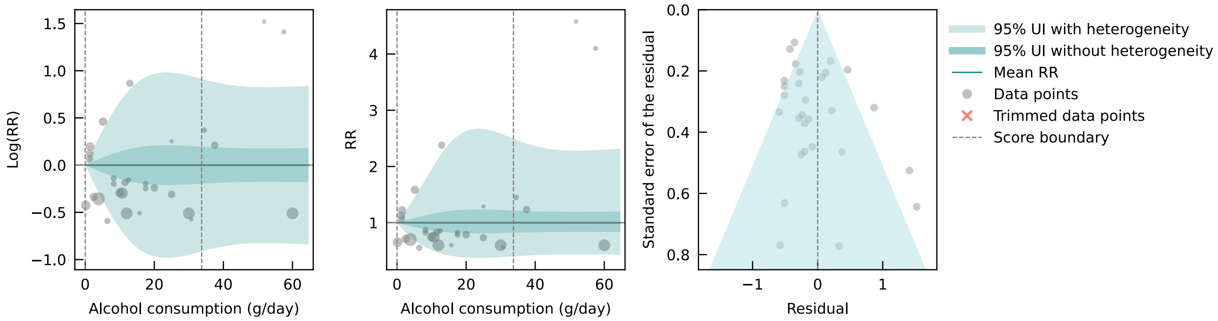

g. Ischemic heart disease mortality – Males

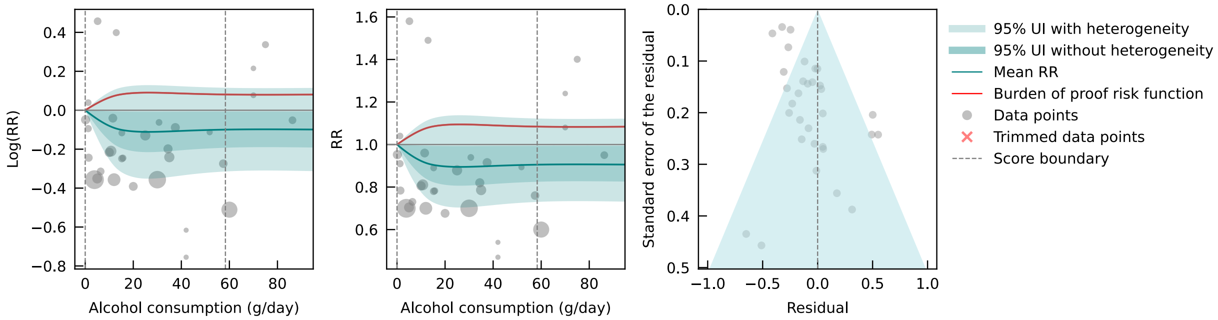

Note. The panels show the log(relative risk) function, the relative risk function, and a modified funnel plot showing the residuals (relative to 0) on the x axis and the estimated standard error that includes the reported standard error and between-study heterogeneity on the y axis. RR = relative risk, UI = uncertainty interval. Source data are provided as a Source Data file.

h. Case-control studies

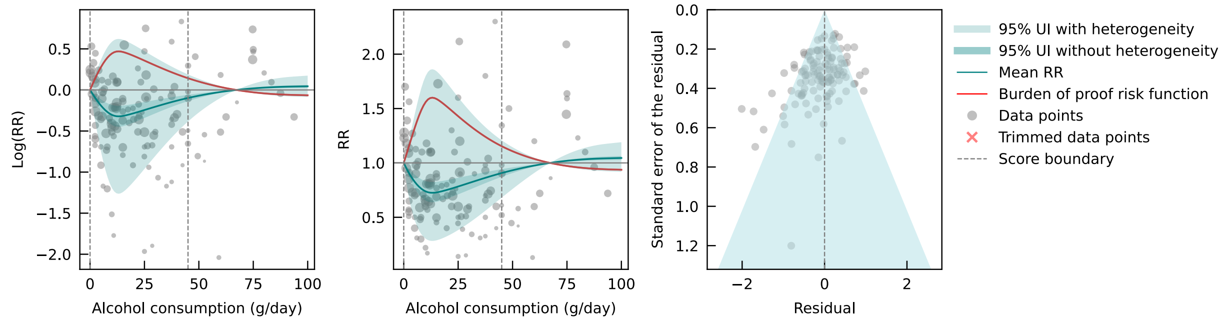

i. Cohort studies

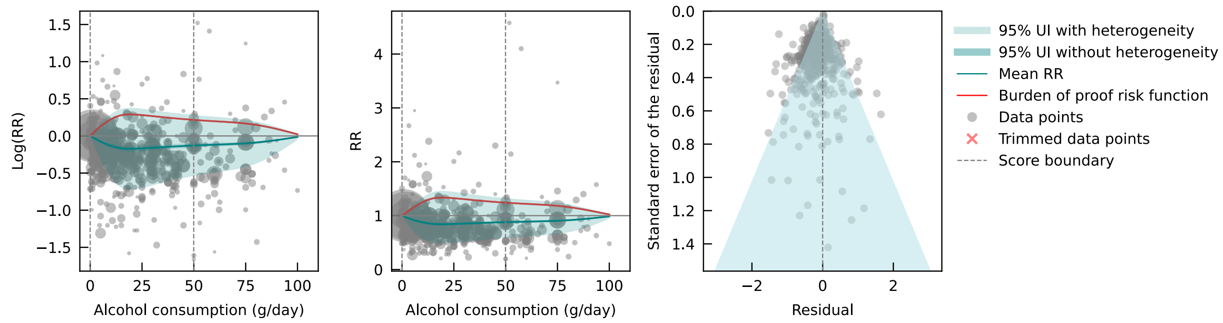

j. Myocardial infarction (morbidity and mortality)

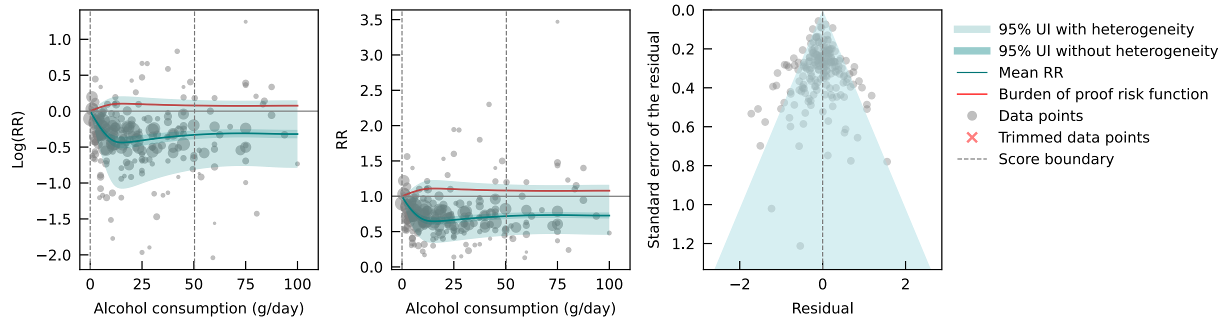

k. Myocardial infarction morbidity

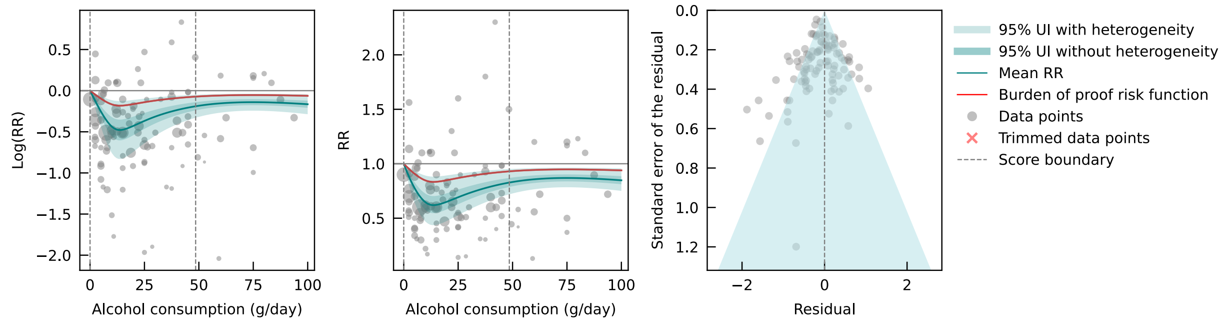

Note. The panels show the log(relative risk) function, the relative risk function, and a modified funnel plot showing the residuals (relative to 0) on the x axis and the estimated standard error that includes the reported standard error and between-study heterogeneity on the y axis. RR = relative risk, UI = uncertainty interval. Source data are provided as a Source Data file.

l. Myocardial infarction mortality

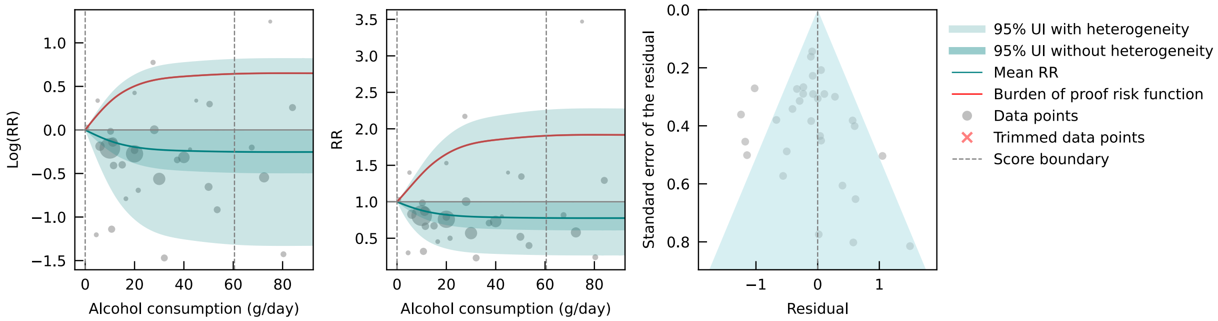

Note. The panels show the log(relative risk) function, the relative risk function, and a modified funnel plot showing the residuals (relative to 0) on the x axis and the estimated standard error that includes the reported standard error and between-study heterogeneity on the y axis. RR = relative risk, UI = uncertainty interval. Source data are provided as a Source Data file.

Table S13. Untrimmed results of the strength of the evidence for the relationship between alcohol consumption and ischemic heart disease

	RR (95% UI) at select exposure levels			Nadir exposure level	RR (95% UI) at nadir	85th percentile risk level	RR (95% UI) at 85th percentile risk level	Exposure-averaged BPRF	Conservative interpretation of the average risk increase/ decrease	ROS	Star rating	Pub. bias	No. of studies
	10 g/day	30 g/day	50 g/day										
Ischemic heart disease	0.87 (0.56, 1.34)	0.84 (0.50, 1.41)	0.87 (0.57, 1.33)	19 g/day	0.83 (0.48, 1.45)	45 g/day	0.86 (0.55, 1.35)	1.26	N/A	-0.23	☆	Yes	122
Morbidity	0.83 (0.53, 1.31)	0.74 (0.36, 1.55)	0.76 (0.39, 1.49)	30 g/day	0.74 (0.36, 1.55)	45 g/day	0.76 (0.38, 1.50)	1.29	N/A	-0.25	☆	Yes	37
Females	0.74 (0.53, 1.05)	0.81 (0.63, 1.03)	0.86 (0.72, 1.02)	15 g/day	0.72 (0.49, 1.05)	26 g/day	0.78 (0.59, 1.04)	0.99	1%	0.01	☆☆	No	6
Males	1.00 (0.26, 3.89)	1.00 (0.19, 5.30)	1.00 (0.48, 2.07)	21 g/day	1.00 (0.15, 6.49)	55 g/day	1.00 (0.58, 1.72)	N/A	N/A	N/A		No	6
Mortality	0.82 (0.49, 1.38)	0.77 (0.39, 1.52)	0.82 (0.48, 1.39)	23 g/day	0.77 (0.38, 1.54)	50 g/day	0.82 (0.48, 1.39)	1.29	N/A	-0.3	☆	Yes	44
Females	1.00 (0.51, 1.95)	1.00 (0.39, 2.59)	1.00 (0.44, 2.28)	23 g/day	1.00 (0.37, 2.67)	34 g/day	1.00 (0.40, 2.49)	N/A	N/A	N/A		Yes	8
Males	0.92 (0.78, 1.10)	0.90 (0.70, 1.14)	0.90 (0.72, 1.13)	26 g/day	0.89 (0.70, 1.14)	58 g/day	0.91 (0.73, 1.12)	1.08	N/A	-0.08	☆	Yes	8
Case-control studies	0.74 (0.30, 1.80)	0.82 (0.45, 1.48)	0.93 (0.76, 1.14)	13 g/day	0.73 (0.28, 1.86)	45 g/day	0.91 (0.68, 1.21)	1.37	N/A	-0.31	☆	Yes	27
Cohort studies	0.87 (0.57, 1.34)	0.85 (0.51, 1.42)	0.88 (0.58, 1.33)	19 g/day	0.84 (0.48, 1.46)	50 g/day	0.88 (0.58, 1.33)	1.26	N/A	-0.23	☆	Yes	95
Myocardial infarction	0.68 (0.38, 1.20)	0.68 (0.38, 1.20)	0.72 (0.44, 1.17)	15 g/day	0.65 (0.34, 1.23)	50 g/day	0.72 (0.44, 1.17)	1.09	N/A	-0.08	☆	Yes	45
Morbidity	0.65 (0.47, 0.89)	0.73 (0.58, 0.92)	0.83 (0.73, 0.95)	14 g/day	0.62 (0.44, 0.88)	49 g/day	0.83 (0.72, 0.95)	0.89	11%	0.12	☆☆	Yes	24
Mortality	0.88 (0.52, 1.50)	0.80 (0.30, 2.09)	0.78 (0.27, 2.23)	80 g/day	0.78 (0.26, 2.28)	61 g/day	0.78 (0.27, 2.26)	1.67	N/A	-0.5	☆	Yes	9

Note. The reported relative risk (RR) and its 95% uncertainty interval (UI) reflect the risk an individual who has been exposed to alcohol consumption has of developing ischemic heart disease or myocardial infarction relative to that of someone who does not drink alcohol (i.e., has zero intake). We report the 95% UI that incorporates unexplained between-study heterogeneity. The Burden of Proof Risk Function (BPRF) is calculated for risk-outcome pairs that were found to have significant relationships at an 0.05 level of significance when not incorporating between-study heterogeneity in the 95% UI. The BPRF corresponds to the 5th or 95th quantile estimate of relative risk accounting for between-study heterogeneity closest to the null for each relationship, and it reflects a conservative estimate of excess risk or risk reduction associated with alcohol consumption that is consistent with the available data. Since we define alcohol consumption as a continuous risk factor, the risk-outcome score (ROS) is calculated as the signed value of the log RR of the BPRF averaged between the 15th and 85th percentiles of exposure levels observed across studies. Negative ROSs indicate that the evidence of the association is weak and inconsistent. For ease of interpretation, we have transformed the ROS and BPRF into a star rating (1-5) with a higher rating representing a larger effect with stronger evidence. The potential existence of publication bias, which, if present, would affect the validity of the results, was tested using Egger's Regression. Included studies represent all available relevant data identified through our systematic reviews from January 1970 through December 2021. N/A = not available.

Section 10.2: Results with effect estimates derived from other Mendelian randomization methods

Figure S6a-c. Relative risk of alcohol consumption for ischemic heart disease, based on data from studies that used other Mendelian randomization methods

a. Inverse variance weighted estimates

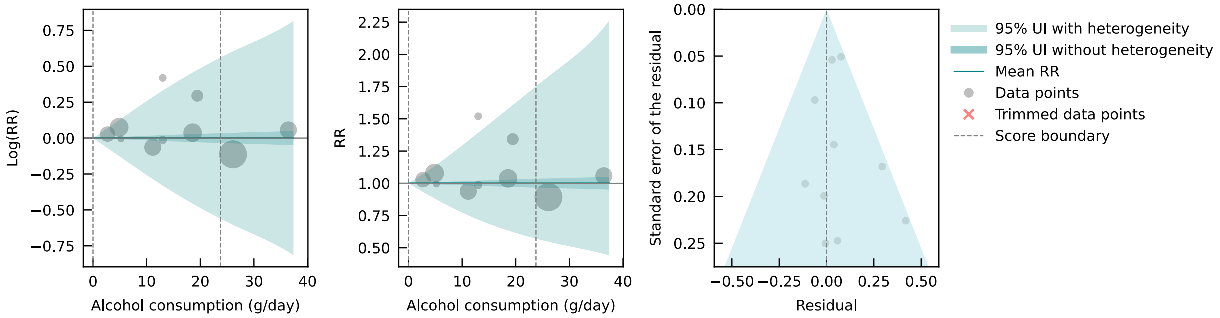

b. Multivariable Mendelian randomization estimates

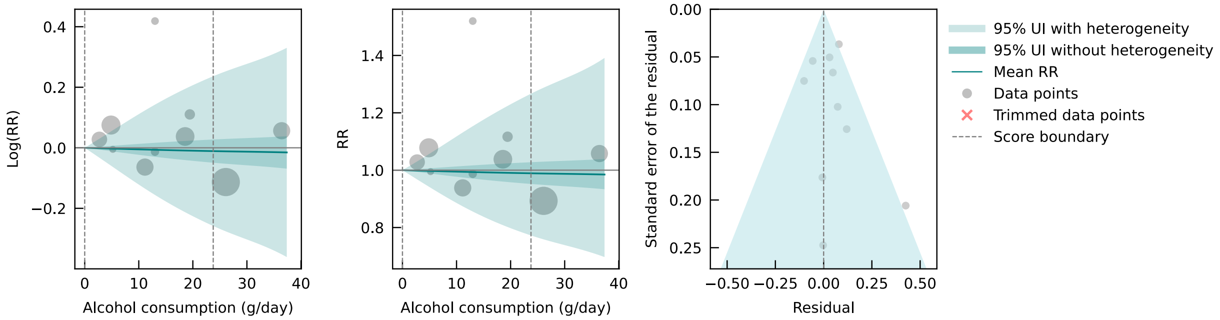

c. Non-linear Mendelian randomization estimates

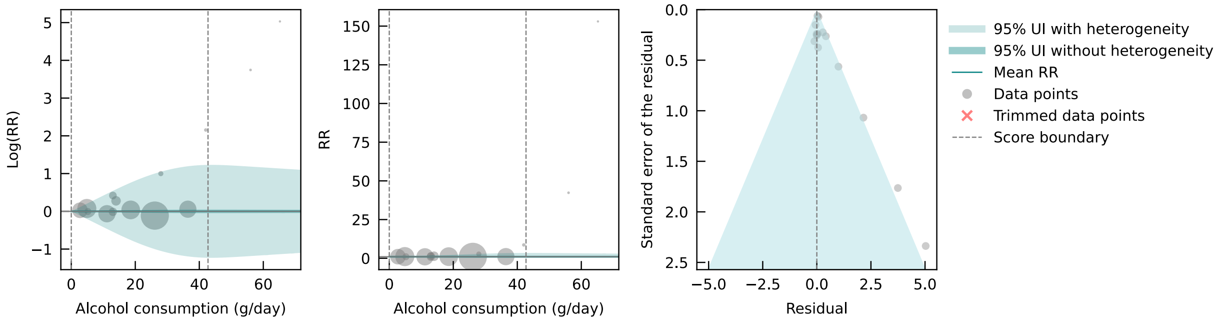

Note. The panels show the log(relative risk) function, the relative risk function, and a modified funnel plot showing the residuals (relative to 0) on the x axis and the estimated standard error that includes the reported standard error and between-study heterogeneity on the y axis. RR = relative risk, UI = uncertainty interval. Source data are provided as a Source Data file.

Section 10.3: Results of conventionally estimated effect sizes from Mendelian randomization studies

Figure S7. Relative risk of alcohol consumption on ischemic heart disease, based on data from conventional estimates from Mendelian randomization studies

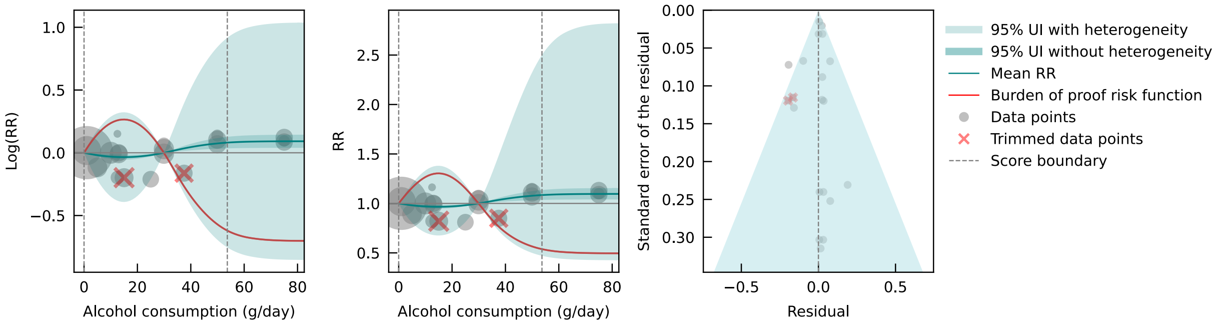

Note. The panels show the log(relative risk) function, the relative risk function, and a modified funnel plot showing the residuals (relative to 0) on the x axis and the estimated standard error that includes the reported standard error and between-study heterogeneity on the y axis. RR = relative risk, UI = uncertainty interval. Source data are provided as a Source Data file.

Section 10.4: Effect estimates from cohort studies conducted in the same geographical locations as Mendelian randomization studies.

Figure S8. Relative risk of alcohol consumption on ischemic heart disease, based on data from cohort studies conducted in the same locations as the Mendelian randomization studies

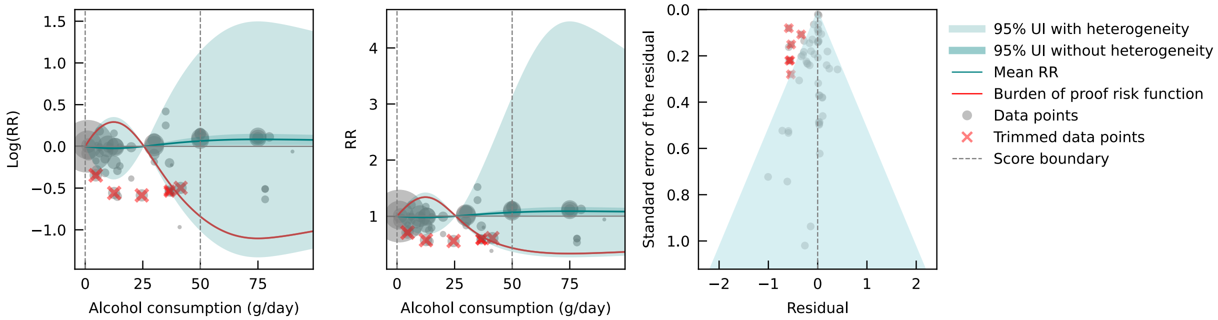

Note. The panels show the log(relative risk) function, the relative risk function, and a modified funnel plot showing the residuals (relative to 0) on the x axis and the estimated standard error that includes the reported standard error and between-study heterogeneity on the y axis. RR = relative risk, UI = uncertainty interval. Source data are provided as a Source Data file.

Table S14. Sensitivity analyses on the strength of the evidence for the relationship between alcohol consumption and ischemic heart disease

	RR (95% UI) at select exposure levels			Nadir exposure level	RR (95% UI) at nadir	85th percentile risk level	RR (95% UI) at 85th percentile risk level	Exposure-averaged BPRF	Conservative interpretation of the average risk increase/ decrease	ROS	Star rating	Pub. bias	No. of studies
	10 g/day	30 g/day	50 g/day										
Mendelian randomization – IVW	1.00 (0.77, 1.29)	1.00 (0.51, 1.95)	N/A	0 g/day	N/A	24 g/day	1.00 (0.57, 1.76)	N/A	N/A	N/A		No	4
Mendelian randomization – MVMR	0.99 (0.89, 1.12)	0.99 (0.74, 1.32)	N/A	37 g/day	0.98 (0.70, 1.39)	24 g/day	0.99 (0.77, 1.27)	N/A	N/A	N/A		No	4
Mendelian randomization – NLMR	1.00 (0.66, 1.52)	1.00 (0.34, 2.99)	1.00 (0.30, 3.37)	0 g/day	N/A	43 g/day	1.00 (0.29, 3.42)	N/A	N/A	N/A		Yes	4
Mendelian randomization – Conventional estimates	0.97 (0.71, 1.33)	1.00 (0.98, 1.02)	1.08 (0.50, 2.32)	15 g/day	0.97 (0.68, 1.38)	54 g/day	1.08 (0.47, 2.51)	0.76	N/A	-0.3	☆	No	4
Cohort studies in the same location	0.98 (0.68, 1.40)	1.01 (0.80, 1.28)	1.07 (0.36, 3.13)	12 g/day	0.98 (0.67, 1.42)	50 g/day	1.07 (0.36, 3.13)	0.73	N/A	-0.32	☆	Yes	13

Note. The reported relative risk (RR) and its 95% uncertainty interval (UI) reflect the risk an individual who has been exposed to alcohol consumption has of developing ischemic heart disease relative to that of someone who does not drink alcohol (i.e., has zero intake). We report the 95% UI that incorporates unexplained between-study heterogeneity. The Burden of Proof Risk Function (BPRF) is calculated for risk-outcome pairs that were found to have significant relationships at an 0.05 level of significance when not incorporating between-study heterogeneity in the 95% UI. The BPRF corresponds to the 5th or 95th quantile estimate of relative risk accounting for between-study heterogeneity closest to the null for each relationship, and it reflects a conservative estimate of excess risk or risk reduction associated with alcohol consumption that is consistent with the available data. Since we define alcohol consumption as a continuous risk factor, the risk-outcome score (ROS) is calculated as the signed value of the log RR of the BPRF averaged between the 15th and 85th percentiles of exposure levels observed across studies. Negative ROSs indicate that the evidence of the association is weak and inconsistent. For ease of interpretation, we have transformed the ROS and BPRF into a star rating (1-5) with a higher rating representing a larger effect with stronger evidence. The potential existence of publication bias, which, if present, would affect the validity of the results, was tested using Egger's Regression. Included studies represent all available relevant data identified through our systematic reviews from January 1970 through December 2021. IVW = inverse varaiance weighted, MVMR = multivariable Mendelian randomization, NLMR = non-linear Mendelian randomization, N/A = not available.

References

1. Albert, C. M. *et al.* Moderate alcohol consumption and the risk of sudden cardiac death among US male physicians. *Circulation* **100**, 944–950 (1999).
2. Arriola, L. *et al.* Alcohol intake and the risk of coronary heart disease in the Spanish EPIC cohort study. *Heart* **96**, 124–130 (2010).
3. Au Yeung, S. L. *et al.* Moderate alcohol use and cardiovascular disease from Mendelian randomization. *PLOS ONE* **8**, e68054 (2013).
4. Augustin, L. S. A. *et al.* Alcohol consumption and acute myocardial infarction: a benefit of alcohol consumed with meals? *Epidemiology* **15**, 767–769 (2004).
5. Bazzano, L. A. *et al.* Alcohol consumption and risk of coronary heart disease among Chinese men. *International Journal of Cardiology* **135**, 78–85 (2009).
6. Bell, S. *et al.* Association between clinically recorded alcohol consumption and initial presentation of 12 cardiovascular diseases: population based cohort study using linked health records. *BMJ* **356**, j909 (2017).
7. Bergmann, M. M. *et al.* The association of pattern of lifetime alcohol use and cause of death in the European prospective investigation into cancer and nutrition (EPIC) study. *International Journal of Epidemiology* **42**, 1772–1790 (2013).
8. Beulens, J. W. J. *et al.* Alcohol consumption and risk for coronary heart disease among men with hypertension. *Annals of Internal Medicine* **146**, 10–19 (2007).
9. Bianchi, C., Negri, E., La Vecchia, C. & Franceschi, S. Alcohol consumption and the risk of acute myocardial infarction in women. *Journal of Epidemiology and Community Health* **47**, 308–311 (1993).
10. Biddinger, K. J. *et al.* Association of habitual alcohol intake with risk of cardiovascular disease. *JAMA Network Open* **5**, e223849–e223849 (2022).
11. Bobak, M. *et al.* Alcohol, drinking pattern and all-cause, cardiovascular and alcohol-related mortality in Eastern Europe. *European Journal of Epidemiology* **31**, 21–30 (2016).
12. Boffetta, P. & Garfinkel, L. Alcohol drinking and mortality among men enrolled in an American Cancer Society prospective study. *Epidemiology* **1**, 342–348 (1990).
13. Brenner, H. *et al.* Coronary heart disease risk reduction in a predominantly beer-drinking population. *Epidemiology* **12**, 390–395 (2001).

14. Britton, A. & Marmot, M. Different measures of alcohol consumption and risk of coronary heart disease and all-cause mortality: 11-year follow-up of the Whitehall II Cohort Study. *Addiction* **99**, 109–116 (2004).

15. Camargo, C. A. *et al.* Moderate alcohol consumption and risk for angina pectoris or myocardial infarction in U.S. male physicians. *Annals of Internal Medicine* **126**, 372–375 (1997).

16. Chang, J. Y., Choi, S. & Park, S. M. Association of change in alcohol consumption with cardiovascular disease and mortality among initial nondrinkers. *Scientific Reports* **10**, 13419 (2020).

17. Chiuve, S. E. *et al.* Light-to-moderate alcohol consumption and risk of sudden cardiac death in women. *Heart Rhythm* **7**, 1374–1380 (2010).

18. Cho, Y. *et al.* Alcohol intake and cardiovascular risk factors: A Mendelian randomisation study. *Scientific Reports* **5**, 18422 (2015).

19. Colditz, G. A. *et al.* Moderate alcohol and decreased cardiovascular mortality in an elderly cohort. *American Heart Journal* **109**, 886–889 (1985).

20. Dai, J., Mukamal, K. J., Krasnow, R. E., Swan, G. E. & Reed, T. Higher usual alcohol consumption was associated with a lower 41-y mortality risk from coronary artery disease in men independent of genetic and common environmental factors: the prospective NHLBI Twin Study. *American Journal of Clinical Nutrition* **102**, 31–39 (2015).

21. Dam, M. K. *et al.* Five year change in alcohol intake and risk of breast cancer and coronary heart disease among postmenopausal women: prospective cohort study. *BMJ* **353**, i2314 (2016).

22. Degerud, E. *et al.* Associations of binge drinking with the risks of ischemic heart disease and stroke: a study of pooled Norwegian Health Surveys. *American Journal of Epidemiology* **190**, 1592–1603 (2021).

23. de Labry, L. O. *et al.* Alcohol consumption and mortality in an American male population: recovering the U-shaped curve--findings from the normative Aging Study. *Journal of Studies on Alcohol* **53**, 25–32 (1992).

24. Doll, R., Peto, R., Boreham, J. & Sutherland, I. Mortality in relation to alcohol consumption: a prospective study among male British doctors. *International Journal of Epidemiology* **34**, 199–204 (2005).

25. Dorn, J. M. *et al.* Alcohol drinking pattern and non-fatal myocardial infarction in women. *Addiction* **102**, 730–739 (2007).

26. Dyer, A. R. *et al.* Alcohol consumption and 17-year mortality in the Chicago Western Electric Company study. *Preventive Medicine* **9**, 78–90 (1980).

27. Ebbert, J. O., Janney, C. A., Sellers, T. A., Folsom, A. R. & Cerhan, J. R. The association of alcohol consumption with coronary heart disease mortality and cancer incidence varies by smoking history. *Journal of General Internal Medicine* **20**, 14–20 (2005).
28. Ebrahim, S. *et al.* Alcohol dehydrogenase type 1C (ADH1C) variants, alcohol consumption traits, HDL-cholesterol and risk of coronary heart disease in women and men: British Women's Heart and Health Study and Caerphilly cohorts. *Atherosclerosis* **196**, 871–878 (2008).
29. Fan, A. Z., Ruan, W. J. & Chou, S. P. Re-examining the relationship between alcohol consumption and coronary heart disease with a new lens. *Preventive Medicine* **118**, 336–343 (2019).
30. Friedman, L. A. & Kimball, A. W. Coronary heart disease mortality and alcohol consumption in Framingham. *Am J Epidemiol* **124**, 481–489 (1986).
31. Fuchs, F. D. *et al.* Association between alcoholic beverage consumption and incidence of coronary heart disease in whites and blacks: the Atherosclerosis Risk in Communities Study. *Am J Epidemiol* **160**, 466–474 (2004).
32. Fumeron, F. *et al.* Alcohol intake modulates the effect of a polymorphism of the cholesteryl ester transfer protein gene on plasma high density lipoprotein and the risk of myocardial infarction. *Journal of Clinical Investigation* **96**, 1664–1671 (1995).
33. Garfinkel, L., Boffetta, P. & Stellman, S. D. Alcohol and breast cancer: a cohort study. *Prev Med* **17**, 686–693 (1988).
34. Gaziano, J. M. *et al.* Moderate alcohol intake, increased levels of high-density lipoprotein and its subfractions, and decreased risk of myocardial infarction. *The New England Journal of Medicine* **329**, 1829–1834 (1993).
35. Gémes, K. *et al.* Alcohol consumption is associated with a lower incidence of acute myocardial infarction: results from a large prospective population-based study in Norway. *Journal of Internal Medicine* **279**, 365–375 (2016).
36. Genchev, G. D., Georgieva, L. M., Weijenberg, M. P. & Powles, J. W. Does alcohol protect against ischaemic heart disease in Bulgaria? A case-control study of non-fatal myocardial infarction in Sofia. *Central European Journal of Public Health* **9**, 83–86 (2001).
37. Gigleux, I. *et al.* Moderate alcohol consumption is more cardioprotective in men with the metabolic syndrome. *The Journal of Nutrition* **136**, 3027–3032 (2006).

38. Goldberg, R. J., Burchfiel, C. M., Reed, D. M., Wergowske, G. & Chiu, D. A prospective study of the health effects of alcohol consumption in middle-aged and elderly men. The Honolulu Heart Program. *Circulation* **89**, 651–659 (1994).
39. Goldberg, R. J. *et al.* Lifestyle and biologic factors associated with atherosclerotic disease in middle-aged men. 20-year findings from the Honolulu Heart Program. *Archives of Internal Medicine* **155**, 686–694 (1995).
40. Gordon, T. & Doyle, J. T. Drinking and coronary heart disease: the Albany Study. *American Heart Journal* **110**, 331–334 (1985).
41. Gun, R. T., Pratt, N., Ryan, P., Gordon, I. & Roder, D. Tobacco and alcohol-related mortality in men: estimates from the Australian cohort of petroleum industry workers. *Australian and New Zealand Journal of Public Health* **30**, 318–324 (2006).
42. Hammar, N., Romelsjö, A. & Alfredsson, L. Alcohol consumption, drinking pattern and acute myocardial infarction. A case referent study based on the Swedish Twin Register. *Journal of Internal Medicine* **241**, 125–131 (1997).
43. Harriss, L. R. *et al.* Alcohol consumption and cardiovascular mortality accounting for possible misclassification of intake: 11-year follow-up of the Melbourne Collaborative Cohort Study. *Addiction* **102**, 1574–1585 (2007).
44. Hart, C. L. & Smith, G. D. Alcohol consumption and mortality and hospital admissions in men from the Midspan collaborative cohort study. *Addiction* **103**, 1979–1986 (2008).
45. Henderson, S. O. *et al.* Established risk factors account for most of the racial differences in cardiovascular disease mortality. *PLOS ONE* **2**, e377 (2007).
46. Hines, L. M. *et al.* Genetic variation in alcohol dehydrogenase and the beneficial effect of moderate alcohol consumption on myocardial infarction. *The New England Journal of Medicine* **344**, 549–555 (2001).
47. Hippe, M. *et al.* Familial predisposition and susceptibility to the effect of other risk factors for myocardial infarction. *Journal of Epidemiology and Community Health* **53**, 269–276 (1999).
48. Ikehara, S. *et al.* Alcohol consumption and mortality from stroke and coronary heart disease among Japanese men and women: the Japan collaborative cohort study. *Stroke* **39**, 2936–2942 (2008).
49. Ikehara, S. *et al.* Alcohol consumption, social support, and risk of stroke and coronary heart disease among Japanese men: the JPHC Study. *Alcoholism, Clinical and Experimental Research* **33**, 1025–1032 (2009).

50. Ilic, M., Grujicic Sipetic, S., Ristic, B. & Ilic, I. Myocardial infarction and alcohol consumption: a case-control study. *PLOS ONE* **13**, e0198129 (2018).

51. Iso, H. *et al.* Alcohol intake and the risk of cardiovascular disease in middle-aged Japanese men. *Stroke* **26**, 767–773 (1995).

52. Jackson, R., Scragg, R. & Beaglehole, R. Alcohol consumption and risk of coronary heart disease. *BMJ* **303**, 211–216 (1991).

53. Jakovljević, B., Stojanov, V., Paunović, K., Belojević, G. & Milić, N. Alcohol consumption and mortality in Serbia: twenty-year follow-up study. *Croatian Medical Journal* **45**, 764–768 (2004).

54. Kabagambe, E. K., Baylin, A., Ruiz-Narvaez, E., Rimm, E. B. & Campos, H. Alcohol intake, drinking patterns, and risk of nonfatal acute myocardial infarction in Costa Rica. *The American Journal of Clinical Nutrition* **82**, 1336–1345 (2005).

55. Kalandidi, A. *et al.* A case-control study of coronary heart disease in Athens, Greece. *International Journal of Epidemiology* **21**, 1074–1080 (1992).

56. Kaufman, D. W., Rosenberg, L., Helmrich, S. P. & Shapiro, S. Alcoholic beverages and myocardial infarction in young men. *American Journal of Epidemiology* **121**, 548–554 (1985).

57. Kawanishi, M., Nakamoto, A., Konemori, G., Horiuchi, I. & Kajiyama, G. Coronary sclerosis risk factors in males with special reference to lipoproteins and apoproteins: establishing an index. *Hiroshima Journal of Medical Sciences* **39**, 61–64 (1990).

58. Keil, U., Chambless, L. E., Döring, A., Filipiak, B. & Stieber, J. The relation of alcohol intake to coronary heart disease and all-cause mortality in a beer-drinking population. *Epidemiology* **8**, 150–156 (1997).

59. Key, T. J. *et al.* Mortality in British vegetarians: results from the European Prospective Investigation into Cancer and Nutrition (EPIC-Oxford). *American Journal of Clinical Nutrition* **89**, 1613S-1619S (2009).

60. Kitamura, A. *et al.* Alcohol intake and premature coronary heart disease in urban Japanese men. *American Journal of Epidemiology* **147**, 59–65 (1998).

61. Kivelä, S. L. *et al.* Alcohol consumption and mortality in aging or aged Finnish men. *Journal of Clinical Epidemiology* **42**, 61–68 (1989).

62. Klatsky, A. L. *et al.* Alcohol drinking and risk of hospitalization for heart failure with and without associated coronary artery disease. *American Journal of Cardiology* **96**, 346–351 (2005).

63. Kono, S., Ikeda, M., Tokudome, S., Nishizumi, M. & Kuratsune, M. Alcohol and mortality: a cohort study of male Japanese physicians. *International Journal of Epidemiology* **15**, 527–532 (1986).

64. Kono, S. *et al.* Alcohol intake and nonfatal acute myocardial infarction in Japan. *American Journal of Cardiology* **68**, 1011–1014 (1991).

65. Kunutsor, S. K. *et al.* Self-reported alcohol consumption, carbohydrate deficient transferrin and risk of cardiovascular disease: The PREVEND prospective cohort study. *Clinica Chimica Acta* **520**, 1–7 (2021).

66. Kurl, S., Jae, S. Y., Voutilainen, A. & Laukkanen, J. A. The combined effect of blood pressure and C-reactive protein with the risk of mortality from coronary heart and cardiovascular diseases. *Nutrition, Metabolism, and Cardiovascular Diseases* **31**, 2051–2057 (2021).

67. Lankester, J., Zanetti, D., Ingelsson, E. & Assimes, T. L. Alcohol use and cardiometabolic risk in the UK Biobank: A Mendelian randomization study. *PLOS ONE* **16**, e0255801 (2021).

68. Larsson, S. C., Wallin, A. & Wolk, A. Contrasting association between alcohol consumption and risk of myocardial infarction and heart failure: two prospective cohorts. *International Journal of Cardiology* **231**, 207–210 (2017).

69. Lazarus, N. B., Kaplan, G. A., Cohen, R. D. & Leu, D. J. Change in alcohol consumption and risk of death from all causes and from ischaemic heart disease. *BMJ* **303**, 553–556 (1991).

70. Lee, D.-H., Folsom, A. R. & Jacobs, D. R. Dietary iron intake and Type 2 diabetes incidence in postmenopausal women: the Iowa Women's Health Study. *Diabetologia* **47**, 185–194 (2004).

71. Liao, Y., McGee, D. L., Cao, G. & Cooper, R. S. Alcohol intake and mortality: findings from the National Health Interview Surveys (1988 and 1990). *American Journal of Epidemiology* **151**, 651–659 (2000).

72. Licaj, I. *et al.* Alcohol consumption over time and mortality in the Swedish Women's Lifestyle and Health cohort. *BMJ Open* **6**, e012862 (2016).

73. Lindschou Hansen, J. *et al.* Alcohol intake and risk of acute coronary syndrome and mortality in men and women with and without hypertension. *European Journal of Epidemiology* **26**, 439–447 (2011).

74. Makelä, P., Paljärvi, T. & Poikolainen, K. Heavy and nonheavy drinking occasions, all-cause and cardiovascular mortality and hospitalizations: a follow-up study in a population with a low consumption level. *Journal of Studies on Alcohol* **66**, 722–728 (2005).

75. Malyutina, S. *et al.* Relation between heavy and binge drinking and all-cause and cardiovascular mortality in Novosibirsk, Russia: a prospective cohort study. *The Lancet* **360**, 1448–1454 (2002).

76. Maraldi, C. *et al.* Impact of inflammation on the relationship among alcohol consumption, mortality, and cardiac events: the health, aging, and body composition study. *Archives of Internal Medicine* **166**, 1490–1497 (2006).

77. Marques-Vidal, P. *et al.* Alcohol consumption and cardiovascular disease: differential effects in France and Northern Ireland. The PRIME study. *European Journal of Cardiovascular Prevention and Rehabilitation* **11**, 336–343 (2004).

78. Mehlig, K. *et al.* CETP TaqIB genotype modifies the association between alcohol and coronary heart disease: the INTERGENE case-control study. *Alcohol* **48**, 695–700 (2014).

79. Meisinger, C., Döring, A., Schneider, A., Löwel, H., & KORA Study Group. Serum gamma-glutamyltransferase is a predictor of incident coronary events in apparently healthy men from the general population. *Atherosclerosis* **189**, 297–302 (2006).

80. Merry, A. H. H. *et al.* Smoking, alcohol consumption, physical activity, and family history and the risks of acute myocardial infarction and unstable angina pectoris: a prospective cohort study. *BMC Cardiovascular Disorders* **11**, 13 (2011).

81. Miller, G. J., Beckles, G. L., Maude, G. H. & Carson, D. C. Alcohol consumption: protection against coronary heart disease and risks to health. *International Journal of Epidemiology* **19**, 923–930 (1990).

82. Millwood, I. Y. *et al.* Conventional and genetic evidence on alcohol and vascular disease aetiology: a prospective study of 500 000 men and women in China. *Lancet* **393**, 1831–1842 (2019).

83. Miyake, Y. Risk factors for non-fatal acute myocardial infarction in middle-aged and older Japanese. Fukuoka Heart Study Group. *Japanese Circulation Journal* **64**, 103–109 (2000).

84. Mukamal, K. J., Chiuve, S. E. & Rimm, E. B. Alcohol consumption and risk for coronary heart disease in men with healthy lifestyles. *Archives of Internal Medicine* **166**, 2145–2150 (2006).

85. Ng, R., Sutradhar, R., Yao, Z., Wodchis, W. P. & Rosella, L. C. Smoking, drinking, diet and physical activity-modifiable lifestyle risk factors and their associations with age to first chronic disease. *International Journal of Epidemiology* **49**, 113–130 (2020).

86. Oliveira, A., Barros, H. & Lopes, C. Gender heterogeneity in the association between lifestyles and non-fatal acute myocardial infarction. *Public Health Nutrition* **12**, 1799–1806 (2009).

87. Oliveira, A., Barros, H., Azevedo, A., Bastos, J. & Lopes, C. Impact of risk factors for non-fatal acute myocardial infarction. *European Journal of Epidemiology* **24**, 425–432 (2009).
88. Onat, A. *et al.* Moderate and heavy alcohol consumption among Turks: long-term impact on mortality and cardiometabolic risk. *Archives of the Turkish Society of Cardiology* **37**, 83–90 (2009).
89. Pedersen, J. Ø., Heitmann, B. L., Schnohr, P. & Grønbaek, M. The combined influence of leisure-time physical activity and weekly alcohol intake on fatal ischaemic heart disease and all-cause mortality. *European Heart Journal* **29**, 204–212 (2008).
90. Reddiess, P. *et al.* Alcohol consumption and risk of cardiovascular outcomes and bleeding in patients with established atrial fibrillation. *Canadian Medical Association Journal* **193**, E117–E123 (2021).
91. Rehm, J. T., Bondy, S. J., Sempos, C. T. & Vuong, C. V. Alcohol consumption and coronary heart disease morbidity and mortality. *American Journal of Epidemiology* **146**, 495–501 (1997).
92. Renaud, S. C., Guéguen, R., Schenker, J. & d'Houtaud, A. Alcohol and mortality in middle-aged men from eastern France. *Epidemiology* **9**, 184–188 (1998).
93. Ricci, C. *et al.* Alcohol intake in relation to non-fatal and fatal coronary heart disease and stroke: EPIC-CVD case-cohort study. *BMJ* **361**, k934 (2018).
94. Rimm, E. B. *et al.* Prospective study of alcohol consumption and risk of coronary disease in men. *The Lancet* **338**, 464–468 (1991).
95. Roerecke, M. *et al.* Heavy drinking occasions in relation to ischaemic heart disease mortality-- an 11-22 year follow-up of the 1984 and 1995 US National Alcohol Surveys. *International Journal of Epidemiology* **40**, 1401–1410 (2011).
96. Romelsjö, A. *et al.* Abstention, alcohol use and risk of myocardial infarction in men and women taking account of social support and working conditions: the SHEEP case-control study. *Addiction* **98**, 1453–1462 (2003).
97. Romelsjö, A., Allebeck, P., Andréasson, S. & Leifman, A. Alcohol, mortality and cardiovascular events in a 35 year follow-up of a nationwide representative cohort of 50,000 Swedish conscripts up to age 55. *Alcohol and Alcoholism* **47**, 322–327 (2012).
98. Rostron, B. Alcohol consumption and mortality risks in the USA. *Alcohol and Alcoholism* **47**, 334–339 (2012).
99. Ruidavets, J.-B. *et al.* Patterns of alcohol consumption and ischaemic heart disease in culturally divergent countries: the Prospective Epidemiological Study of Myocardial Infarction (PRIME). *BMJ* **341**, c6077 (2010).

100. Schooling, C. M. *et al.* Moderate alcohol use and mortality from ischaemic heart disease: a prospective study in older Chinese people. *PLOS ONE* **3**, e2370 (2008).

101. Schröder, H. *et al.* Myocardial infarction and alcohol consumption: a population-based case-control study. *Nutrition, Metabolism, and Cardiovascular Diseases* **17**, 609–615 (2007).

102. Schutte, R., Smith, L. & Wannamethee, G. Alcohol - The myth of cardiovascular protection. *Clinical Nutrition* **41**, 348–355 (2022).

103. Scragg, R., Stewart, A., Jackson, R. & Beaglehole, R. Alcohol and exercise in myocardial infarction and sudden coronary death in men and women. *Am J Epidemiol* **126**, 77–85 (1987).

104. Sempos, C., Rehm, J., Crespo, C. & Trevisan, M. No protective effect of alcohol consumption on coronary heart disease (CHD) in African Americans: average volume of drinking over the life course and CHD morbidity and mortality in a U.S. national cohort. *Contemporary Drug Problems* **29**, 805–820 (2002).

105. Shaper, A. G., Wannamethee, G. & Walker, M. Alcohol and coronary heart disease: a perspective from the British Regional Heart Study. *International Journal of Epidemiology* **23**, 482–494 (1994).

106. Simons, L. A., McCallum, J., Friedlander, Y. & Simons, J. Alcohol intake and survival in the elderly: a 77 month follow-up in the Dubbo study. *Australian and New Zealand Journal of Medicine* **26**, 662–670 (1996).

107. Skov-Ettrup, L. S., Eliasen, M., Ekholm, O., Grønbæk, M. & Tolstrup, J. S. Binge drinking, drinking frequency, and risk of ischaemic heart disease: a population-based cohort study. *Scandinavian Journal of Public Health* **39**, 880–887 (2011).

108. Snow, W. M., Murray, R., Ekuma, O., Tyas, S. L. & Barnes, G. E. Alcohol use and cardiovascular health outcomes: a comparison across age and gender in the Winnipeg Health and Drinking Survey Cohort. *Age and Ageing* **38**, 206–212 (2009).

109. Song, R. J. *et al.* Alcohol consumption and risk of coronary artery disease (from the Million Veteran Program). *American Journal of Cardiology* **121**, 1162–1168 (2018).

110. Streppel, M. T., Ocké, M. C., Boshuizen, H. C., Kok, F. J. & Kromhout, D. Long-term wine consumption is related to cardiovascular mortality and life expectancy independently of moderate alcohol intake: the Zutphen Study. *Journal of Epidemiology and Community Health* **63**, 534–540 (2009).

111. Suhonen, O., Aromaa, A., Reunanen, A. & Knekt, P. Alcohol consumption and sudden coronary death in middle-aged Finnish men. *Acta Medica Scandinavica* **221**, 335–341 (1987).

112. Tavani, A., Bertuzzi, M., Gallus, S., Negri, E. & La Vecchia, C. Risk factors for non-fatal acute myocardial infarction in Italian women. *Preventive Medicine* **39**, 128–134 (2004).

113. Tavani, A. *et al.* Intake of specific flavonoids and risk of acute myocardial infarction in Italy. *Public Health Nutrition* **9**, 369–374 (2006).

114. Thun, M. J. *et al.* Alcohol consumption and mortality among middle-aged and elderly U.S. adults. *The New England Journal of Medicine* **337**, 1705–1714 (1997).

115. Tolstrup, J. *et al.* Prospective study of alcohol drinking patterns and coronary heart disease in women and men. *BMJ* **332**, 1244–1248 (2006).

116. Wannamethee, G. & Shaper, A. G. Alcohol and sudden cardiac death. *British Heart Journal* **68**, 443–448 (1992).

117. Wannamethee, S. G. & Shaper, A. G. Type of alcoholic drink and risk of major coronary heart disease events and all-cause mortality. *American Journal of Public Health* **89**, 685–690 (1999).

118. Wilkins, K. Moderate alcohol consumption and heart disease. *Health Reports* **14**, 9–24 (2002).

119. Yang, L. *et al.* Alcohol drinking and overall and cause-specific mortality in China: nationally representative prospective study of 220,000 men with 15 years of follow-up. *International Journal of Epidemiology* **41**, 1101–1113 (2012).

120. Yi, S. W., Yoo, S. H., Sull, J. W. & Ohrr, H. Association between alcohol drinking and cardiovascular disease mortality and all-cause mortality: Kangwha Cohort Study. *Journal of Preventive Medicine and Public Health* **37**, 120–126 (2004).

121. Younis, J., Cooper, J. A., Miller, G. J., Humphries, S. E. & Talmud, P. J. Genetic variation in alcohol dehydrogenase 1C and the beneficial effect of alcohol intake on coronary heart disease risk in the Second Northwick Park Heart Study. *Atherosclerosis* **180**, 225–232 (2005).

122. Yusuf, S. *et al.* Modifiable risk factors, cardiovascular disease, and mortality in 155 722 individuals from 21 high-income, middle-income, and low-income countries (PURE): a prospective cohort study. *The Lancet* **395**, 795–808 (2020).

123. Zhang, Y. *et al.* Association of drinking pattern with risk of coronary heart disease incidence in the middle-aged and older Chinese men: results from the Dongfeng-Tongji cohort. *PLOS ONE* **12**, e0178070 (2017).

124. Zhou, X., Li, C., Xu, W., Hong, X. & Chen, J. Relation of alcohol consumption to angiographically proved coronary artery disease in chinese men. *American Journal of Cardiology* **106**, 1101–1103 (2010).
