# Supplementary material for: A burden of proof study on alcohol consumption and ischemic heart disease
Source: Nat Commun. 2024 May 14;15:4082. doi: 10.1038/s41467-024-47632-7 (PMC11094064; doi:10.1038/s41467-024-47632-7)
Supplement: Supplementary file 3 — Reporting Summary [file 41467_2024_47632_MOESM3_ESM.pdf]

nature portfolio

Corresponding author(s): Sinclair Carr

Last updated by author(s): Mar 19, 2024

Reporting Summary

Nature Portfolio wishes to improve the reproducibility of the work that we publish. This form provides structure for consistency and transparency in reporting. For further information on Nature Portfolio policies, see our Editorial Policies and the Editorial Policy Checklist.

Statistics

For all statistical analyses, confirm that the following items are present in the figure legend, table legend, main text, or Methods section.

n/a	Confirmed	
☐	☒	The exact sample size (*n*) for each experimental group/condition, given as a discrete number and unit of measurement
☐	☒	A statement on whether measurements were taken from distinct samples or whether the same sample was measured repeatedly
☐	☒	The statistical test(s) used AND whether they are one- or two-sided<br>*Only common tests should be described solely by name; describe more complex techniques in the Methods section.*
☐	☒	A description of all covariates tested
☐	☒	A description of any assumptions or corrections, such as tests of normality and adjustment for multiple comparisons
☐	☒	A full description of the statistical parameters including central tendency (e.g. means) or other basic estimates (e.g. regression coefficient) AND variation (e.g. standard deviation) or associated estimates of uncertainty (e.g. confidence intervals)
☐	☒	For null hypothesis testing, the test statistic (e.g. *F*, *t*, *r*) with confidence intervals, effect sizes, degrees of freedom and *P* value noted<br>*Give P values as exact values whenever suitable.*
☐	☒	For Bayesian analysis, information on the choice of priors and Markov chain Monte Carlo settings
☒	☐	For hierarchical and complex designs, identification of the appropriate level for tests and full reporting of outcomes
☐	☒	Estimates of effect sizes (e.g. Cohen's *d*, Pearson's *r*), indicating how they were calculated

Our web collection on statistics for biologists contains articles on many of the points above.

Software and code

Policy information about availability of computer code

Data collection	Relevant data from published cohort, case-control, and Mendelian randomization studies were extracted by a reviewer using a data collection form (see Supplementary Information section 3 Table S4 for a template). No primary data collection was carried out for this analysis.
Data analysis	Analyses were carried out using R version 4.0.5 and Python version 3.10.9. All code used for these analyses is publicly available online (https://github.com/ihmeuw-msca/burden-of-proof).

For manuscripts utilizing custom algorithms or software that are central to the research but not yet described in published literature, software must be made available to editors and reviewers. We strongly encourage code deposition in a community repository (e.g. GitHub). See the Nature Portfolio guidelines for submitting code & software for further information.

Data

Policy information about availability of data

All manuscripts must include a data availability statement. This statement should provide the following information, where applicable:

- Accession codes, unique identifiers, or web links for publicly available datasets
- A description of any restrictions on data availability
- For clinical datasets or third party data, please ensure that the statement adheres to our policy

The findings from this study were produced using data extracted from published literature. The relevant studies were identified through a systematic literature review and can all be accessed online as referenced in the current paper27–30,32,40–158. Further details on the relevant studies can be found on the GHDx

website (https://ghdx.healthdata.org/record/ihme-data/gbd-alcohol-ihd-bop-risk-outcome-scores). Study characteristics of all relevant studies included in the analyses are also provided in Supplementary Information section 4 (Tables S5 and S6). The template of the data collection form is provided in Supplementary Information section 3 (Table S4). The source data include processed data from these studies that underlie our estimates. Source data have been provided with this paper.

Research involving human participants, their data, or biological material

Policy information about studies with human participants or human data. See also policy information about sex, gender (identity/presentation), and sexual orientation and race, ethnicity and racism.

Reporting on sex and gender	No primary data collection was carried out for this analysis, so the study does not involve human research participants. Our estimates based on data from cohort and case-control studies were disaggregated by sex. This analysis only included such studies that reported effect sizes for both females and males to allow direct comparison of risk across different exposure levels; however, we did not collect information about the method each study used to determine sex. We did not disaggregate findings based on Mendelian randomization studies due to insufficient data.
Reporting on race, ethnicity, or other socially relevant groupings	No primary data collection was carried out for this analysis, so the study does not involve human research participants. We extracted the reported ancestry of the study sample investigated in Mendelian randomization studies but did not collect information about the methods each study used to determine ancestry. We did not disaggregate findings by race, ethnicity, or other socially relevant groupings.
Population characteristics	No primary data collection was carried out for this analysis, so the study does not involve human research participants. The analysis evaluated the association between alcohol consumption and ischemic heart disease in adults (18 years and older).
Recruitment	No primary data collection was carried out for this analysis, so we did not recruit participants.
Ethics oversight	This study was approved by the University of Washington IRB Committee (study #9060).

Note that full information on the approval of the study protocol must also be provided in the manuscript.

Field-specific reporting

Please select the one below that is the best fit for your research. If you are not sure, read the appropriate sections before making your selection.

☒ Life sciences ☐ Behavioural & social sciences ☐ Ecological, evolutionary & environmental sciences

For a reference copy of the document with all sections, see nature.com/documents/nr-reporting-summary-flat.pdf

Life sciences study design

All studies must disclose on these points even when the disclosure is negative.

Sample size	No sample size calculation was performed for this meta-analysis; all available datasets meeting the inclusion criteria are included. As reported in the main text results sections, the number of data points for each main analysis on alcohol consumption and ischemic heart disease is as follows: in all conventional observational studies (cohort and case-control studies combined): 7,059,652 participants and 243,357 events; in cohort studies: 6,998,738 participants and 226,465 events; in case-control studies: 60,914 participants and 16,892 cases; in Mendelian randomization studies: 559,708 participants and 22,134 cases.
Data exclusions	As described in the Methods section "Conducting the systematic review", reports were excluded based on the following exclusion criteria: were an aggregate study: meta-analysis or pooled cohort; utilized a study design not designated for inclusion in this analysis: not a cohort study, case-control study, or Mendelian randomization study; were a duplicate study: the underlying sample of the paper had also been analyzed elsewhere (we always considered the analysis with the longest follow-up for cohort studies or the most recently published analysis for Mendelian randomization studies); did not report on the exposure of interest: reported on combined exposure of alcohol and drug use or reported alcohol consumption in a non-continuous way; did not report on the outcome of interest: reported an outcome that was not ischemic heart disease or a composite outcome that included but was not limited to ischemic heart disease, or outcomes lacked specificity, such as cardiovascular disease or all-cause mortality; were not in English; and were animal studies.
Replication	This is a meta-analysis of existing observational studies. After running the statistical models to obtain final results, we ran the models again to create the tables and figures. The code and data used are publicly available, and the analyses could theoretically be replicated.
Randomization	This is a meta-analysis of existing observational studies and thus, there were no experimental groups.
Blinding	This is a meta-analysis of existing observational studies and thus, blinding was not relevant.

Reporting for specific materials, systems and methods

We require information from authors about some types of materials, experimental systems and methods used in many studies. Here, indicate whether each material, system or method listed is relevant to your study. If you are not sure if a list item applies to your research, read the appropriate section before selecting a response.

Materials & experimental systems

n/a	Involved in the study
☒	☐ Antibodies
☒	☐ Eukaryotic cell lines
☒	☐ Palaeontology and archaeology
☒	☐ Animals and other organisms
☒	☐ Clinical data
☒	☐ Dual use research of concern
☒	☐ Plants

Methods

n/a	Involved in the study
☒	☐ ChIP-seq
☒	☐ Flow cytometry
☒	☐ MRI-based neuroimaging

Plants

Seed stocks

Report on the source of all seed stocks or other plant material used. If applicable, state the seed stock centre and catalogue number. If plant specimens were collected from the field, describe the collection location, date and sampling procedures.

Novel plant genotypes

Describe the methods by which all novel plant genotypes were produced. This includes those generated by transgenic approaches, gene editing, chemical/radiation-based mutagenesis and hybridization. For transgenic lines, describe the transformation method, the number of independent lines analyzed and the generation upon which experiments were performed. For gene-edited lines, describe the editor used, the endogenous sequence targeted for editing, the targeting guide RNA sequence (if applicable) and how the editor was applied.

Authentication

Describe any authentication procedures for each seed stock used or novel genotype generated. Describe any experiments used to assess the effect of a mutation and, where applicable, how potential secondary effects (e.g. second site T-DNA insertions, mosiacism, off-target gene editing) were examined.
